# Trinuclear cylinder-like potential anticancer and antibacterial Cu(i), Ag(i) and Au(i) nano-sized cationic complexes with tris-NHC ligands: cationic M_3_ metal cluster displaying positive or negative cooperativity in triad [L_2_(R)_6_→M_3_]^3+^ complexes?†

**DOI:** 10.1039/d4ra08514k

**Published:** 2025-02-28

**Authors:** Khadijeh Naeimi, Mehdi Bayat, Ehsan Alavi Pour

**Affiliations:** a Department of Inorganic Chemistry, Faculty of Chemistry and Petroleum Sciences, Bu-Ali Sina University Hamedan Iran; b School of Chemistry, College of Science, University of Tehran Tehran , Iran bayatm@ut.ac.ir mehdi806@gmail.com

## Abstract

N-Heterocyclic carbenes (NHCs) are a class of organic molecules containing a divalent carbon atom, known as a carbene, within a heterocyclic (ring) structure where nitrogen atoms (N) form part of the ring. These molecules have garnered significant attention in coordination chemistry due to their unique bonding properties, particularly as strong σ-donor ligands that facilitate the formation of stable complexes. A theoretical study was conducted to investigate the structural and bonding characteristics of M←C bonds in trinuclear, nano-sized Cu(i), Ag(i), and Au(i) cations with two tris-NHC ligands, which exhibit promising anti-cancer and antibacterial potential. The study employed natural bond orbital (NBO) techniques, energy decomposition analysis (EDA), and extended transition-state natural orbital for chemical valence (ETS-NOCV) methods to analyze the bonding interactions. The cooperativity values between bonds were also examined, revealing positive values indicative of anti-cooperativity within the complexes. The results further demonstrated that the M←C interactions are predominantly electrostatic in nature. These findings highlight the unique structural and electronic properties of the complexes, suggesting their potential as candidates for anti-cancer and antibacterial applications.

## Abstract 2

N-Heterocyclic carbenes (NHCs) are a class of organic molecules containing a divalent carbon atom, known as a carbene, within a heterocyclic (ring) structure where nitrogen atoms (N) form part of the ring.

## Graphical abstract

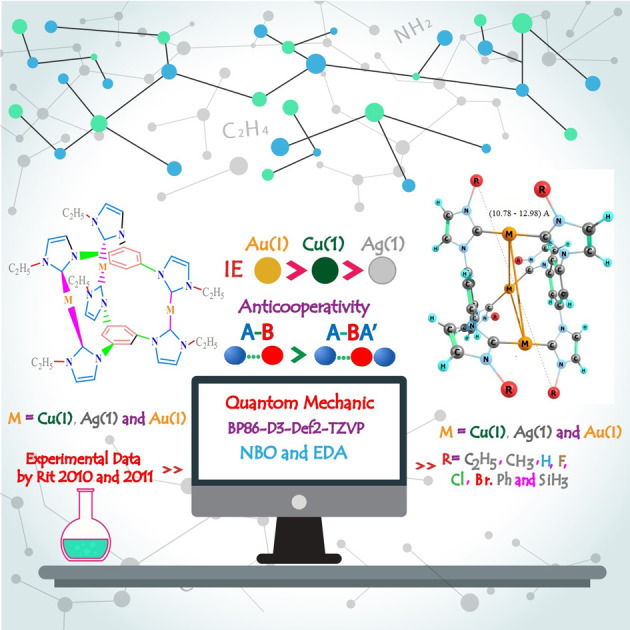

## Introduction

**Fig. 1:**
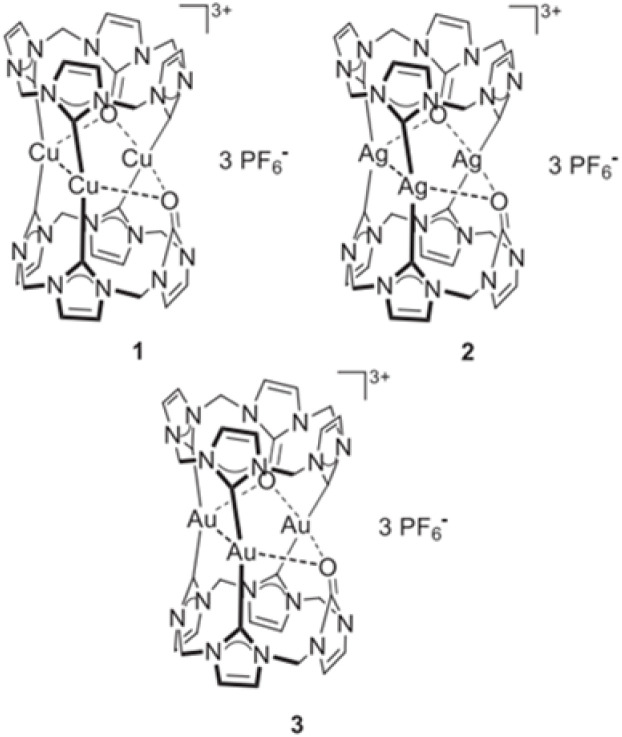
The structure of the pharmaceutical complexes studied by Jacob.

N-Heterocyclic carbenes (NHCs) have emerged as versatile ligands in both organometallic and inorganic chemistry. Owing to their strong σ-donating properties, NHCs form more stable bonds with metals than phosphines,^1^ with the lone pair of electrons on the carbene carbon stabilized by adjacent nitrogen atoms through an inductive effect.^2^ Additionally, metal–NHC complexes have attracted significant attention due to their wide range of catalytic^3,4^ and pharmacological^5–7^ properties. They are extensively used as ligands in inorganic and organometallic chemistry for two primary reasons: first, their strong metal coordination, and second, their resistance to moisture and air.^2,8^ As a result, NHCs are preferred as substitutes for phosphine ligands. Modifications at the (N_1_) position of the NHC ligand significantly impact its reactivity and binding affinity. Transition metal–NHC complexes have been employed in medicinal chemistry due to their remarkable biological properties.^9^ N-heterocyclic carbene–metal complexes have gained significance in fields like organometallic chemistry, catalysis and bioorganic/bioinorganic chemistry. Among these, coinage metal–NHCs are particularly noteworthy due to their varied biological properties and applications.^10,11^ The NHC copper complexes have the potential to generate reactive oxygen species (ROS), which can cause DNA strand breaks and contribute to the observed cytotoxicity.^12^ Metallic silver was recognized by the Chaldeans as early as 4000 B.C.E., making it the third metal used by ancient civilizations, following gold and copper.^13^ Although most organometallic pharmaceutical research has focused on platinum and gold, the medicinal applications of silver are well-documented.^14^ Multinuclear silver N-heterocyclic carbene (Ag-NHC) complexes have garnered interest in medicinal chemistry due to their unique properties and potential applications. These complexes represent a promising area in medicinal chemistry, with potential applications in antimicrobial and anticancer therapies.^15–19^ Gold has long been essential in human history. Likely the first metal to be discovered, it has been utilized in medicinal treatments since ancient times.^20^ Multinuclear gold-N-heterocyclic carbene (Au-NHC) complexes are an evolving area of research in medicinal chemistry, showing promise for various therapeutic applications. These complexes are emerging as a promising class of compounds in medicinal chemistry. They hold potential for applications in both cancer therapy and antimicrobial treatments.^21–26^ In 2021, Jacob reported the synthesis and identification of the first large cyclic trinuclear, tri-carbene complexes Cu(i), Ag(i), and Au(i). The ability of these complexes to inhibit the growth of bacteria (*S. aureus*, *E. coli*) and cancer cells (HeLa, MCF7) was investigated. The copper and silver complexes demonstrated antiproliferative activity in both cell lines, ranging from 3.03 μM to 25.01 μM (ref. 27) (see Fig. 1).^27^ Compared to mononuclear complexes, multinuclear complexes have more active sites that can coordinate with substituents possessing biological properties, and as a result, they may exhibit stronger biological activities.^28^

**Fig. 2:**
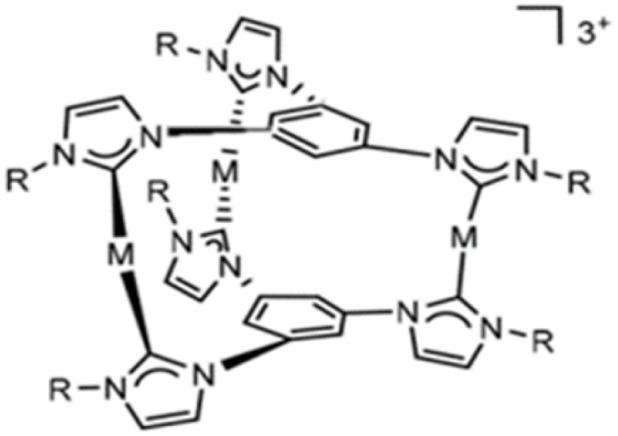
Schematic representation of the [L_2_(R)_6_→M_3_]^3+^ nano-sized cationic complexes.

Recent studies have investigated the structure and nature of metal–NHC bonds in some pharmaceutical coinage metal ion complexes.^29–33^ Cooperativity in bonds refers to the phenomenon where the formation, breaking, or alteration of one bond influences the behavior of nearby bonds. This interaction can enhance or inhibit subsequent bonding events, depending on the system. Cooperativity is widely observed in physical, chemical, and biological systems, and it is particularly important in stabilizing molecular structures and driving complex reactions. Herein, a comparative theoretical investigation into the nature of the metal–NHC bonds of some cylindrical trinuclear clusters of group 11 in coordination with two symmetrical tri-N-heterocyclic carbene (NHC) ligand was reported. The effect of substituents, C_2_H_5_, CH_3_, H, F, Cl, Br, Ph, and SiH_3_ on six NHC rings of cationic three-metal clusters of copper (1), silver (1) and gold (1) are also investigated. It is worth noting that the X-ray crystal structures of three metal clusters, copper, silver and gold, substituted with C_2_H_5_ were synthesized by Rit in 2010 (ref. 34) and 2011.^35^ Our research group has used the crystal structure of these clusters as input files for the calculations in this study Fig. 2.

### Computational details

The [L_2_(C_2_H_5_)_6_→Au_3_]^3+^ complex is analysed using eight density functional methods: Cam-B3LYP, M05-2X, M06-2X, B3LYP, PBE,^35^ BLYP, BP86 and M06, in conjunction with D3 dispersion corrections. Additionally, the structural results obtained were compared with the corresponding experimental values derived from X-ray crystallography.^34,35^ The results obtained from the RMS methodological findings indicate that PBE-D3,^36^ the most relevant functional among the methods mentioned, shows the strongest correlation between quantitative and experimental structural data in Table 1.

**Table 1:** RMS calculation of [L_2_(C_2_H_5_)_6_→Au_3_]^3+^ complex

Method	Au–C (Å)	Au–C (Å)	Au–C (Å)	RMS
B3LYP-D3	2.06	2.06	2.06	0.09
BLYP-D3	2.06	2.06	2.06	0.09
BP86-D3	2.04	2.04	2.04	0.06
CAM-B3LYP-D3	2.05	2.05	2.05	0.08
M05-2X-D3	2.05	2.05	2.05	0.08
M06-D3	2.06	2.06	2.06	0.09
M06-2X-D3	2.05	2.05	2.05	0.08
PBE-D3	2.04	2.04	2.04	0.06
Exp	2.00	2.01	2.00	—

In this article, the cationic parts of the complexes with the general formula [L_2_(R)_6_→M_3_]^3+^ (where M = Cu(i), Ag(i), Au(i); R = C_2_H_5_, CH_3_, H, F, Cl, Br, Ph, and SiH_3_) were investigated. All calculations (optimized structures and single points) were performed at the PBE-D3/def2-TZVP^37,38^ level of theory using Gaussian 09 software.^39^ All structures were optimized and their energies were obtained in gas phase using PBE-D3/def2-TZVP level of theory. The vibrational frequency analysis indicates that the optimized structures at stationary points, obtained at the same theoretical level, correspond to local minima, as evidenced by the absence of imaginary frequencies. NBO analysis calculations were performed to investigate the nature of the

C_(tris-NHC)_→M_3_ bonds using the Gaussian 09 at the BP86/def2-TZVP theoretical level.^40,41^ Energy decomposition analysis (EDA) was conducted to assess the nature of the C_(tris-NHC)_→M_3_ bonds at the BP86-D3/TZ2P level of theory, utilizing the ADF 2013.01 software package.

## Result and discussion

### Structural studies

**Fig. 3:**
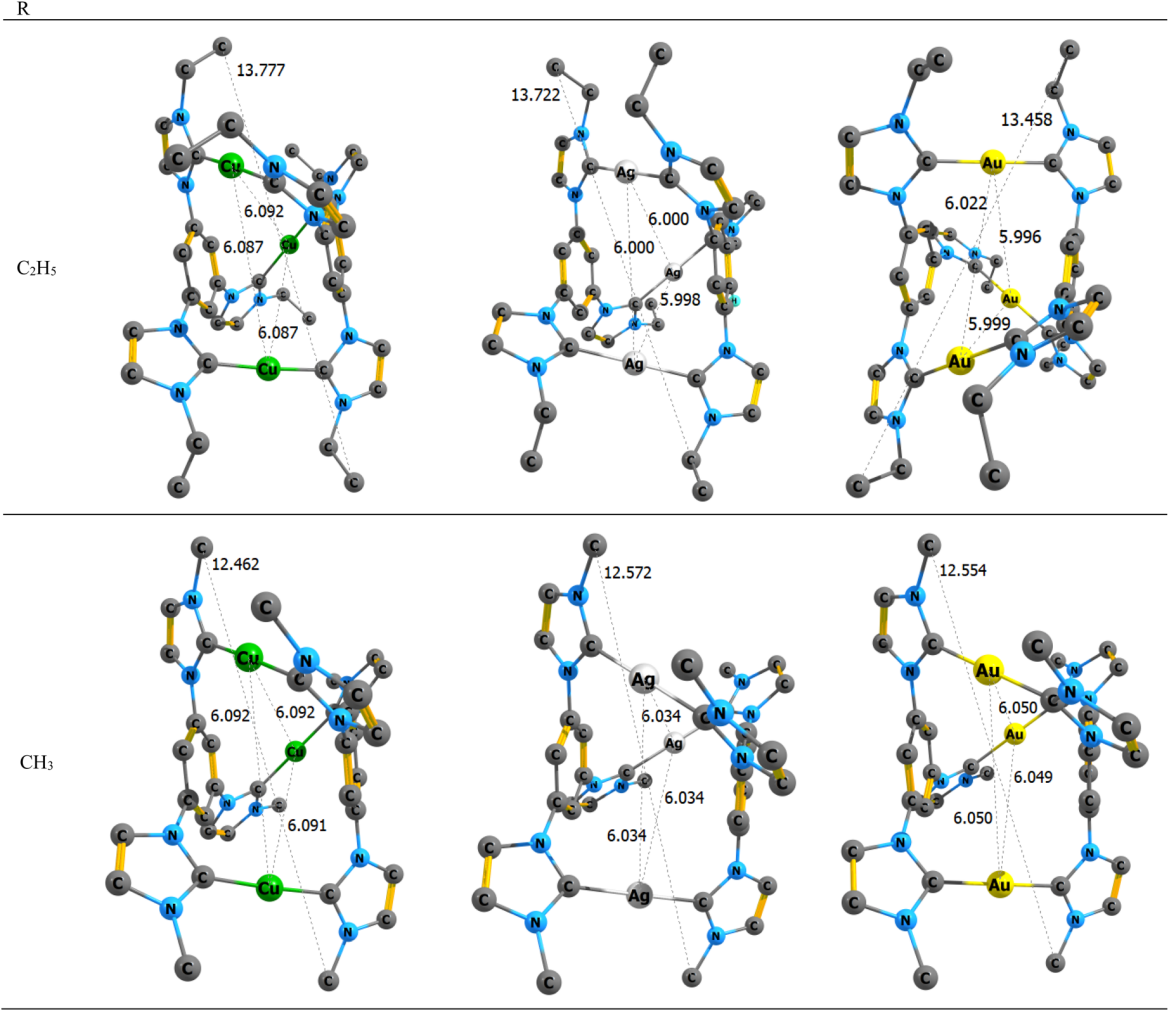
The optimized structures and length of [L_2_(R)_6_→M_3_]^3^ complexes; M = Cu(i), Ag(i), Au(i); R = C_2_H_5_ and CH_3_. All hydrogen atoms were removed to enhance clarity.

The mentioned complexes optimized on PBE-D3/def2TZVP level of theory in the gas phase. The optimized structures and length of the corresponding of [L_2_(R)_6_→M_3_]^3+^ complexes; M = Cu(i), Ag(i), Au(i); R = C_2_H_5_ and CH_3_ are shown in Fig. 3. The optimized structure of the other related complexes has been reported in the Tables S3 and S4.†

To measure the length of complexes, [L_2_(R)_6_→M_3_]^3+^; M = Cu(i), Ag(i), Au(i); R = H, F, Cl, Br, CH_3_ and SiH_3_, the distance between two atoms H, F, Cl, Br, C, and Si located on reciprocal R's was calculated. Similarly, for complexes [L_2_(R)_6_→M_3_]^3+^; M = Cu(i), Ag(i), Au(i); R = C_2_H_5_ and Ph, the distance between two distant carbon atoms located on R was measured.

**Fig. 4:**
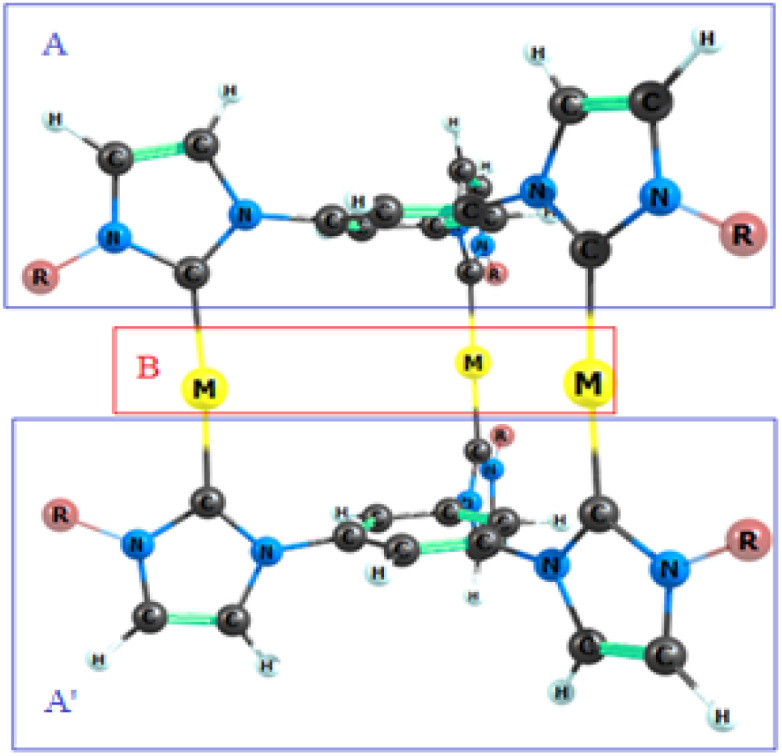
Fragments analysis of [L_2_(R)_6_→M_3_]^3+^ complexes investigated here.

**Fig. 5:**
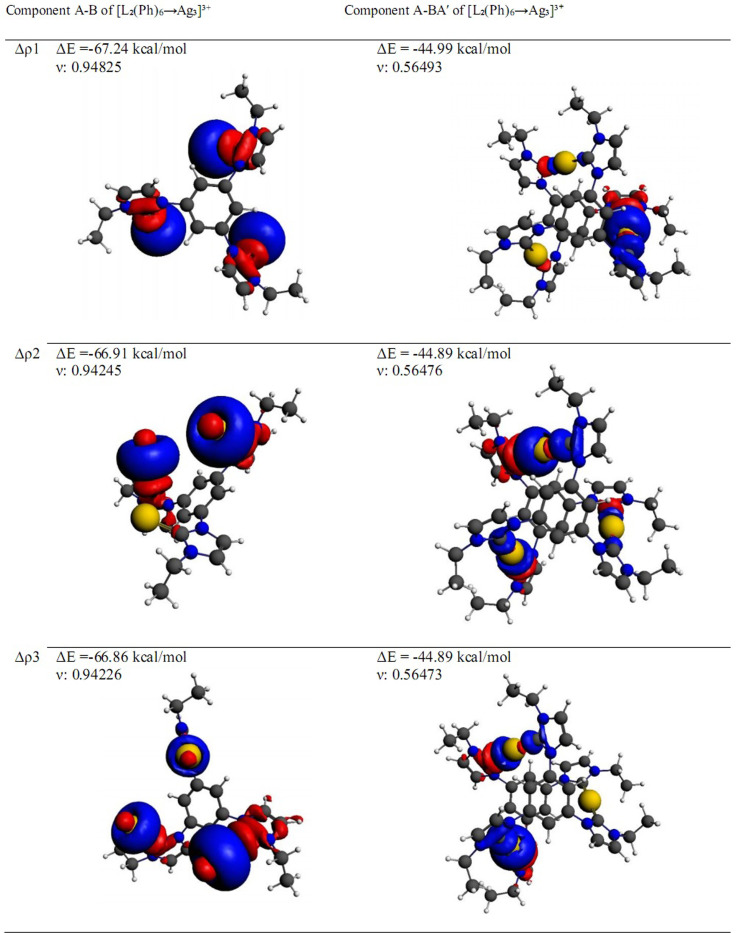
Contour plots of NOCV deformation densities Δ*ρ* and associated energies Δ*E*(*ρ*), computed at the BP86-D3/TZ2P level of theory. The corresponding deformation electron densities were depicted by the direction of charge transitioning from red to blue. The eigenvalues (*ν*) give the size of the charge migration.

Structural data confirmed that this series of complexes has a nanostructure sized (see Fig. 3–5). All coordination details of the Au(i); R = C_2_H_5_, CH_3_, H, F, Cl, Br, Ph, and SiH_3_, were 1.90 Å for information. Optimized complexes mentioned are provided in the supporting copper complexes and 2.08 Å for silver complexes. Also, C_(tris-NHC)_→M_3_ bond lengths for the gold complexes are approximately 2.03 Å. According to the results, a reverse V-shaped trend is observed. With the change in substituents, no significant changes are observed. Due to the symmetrical nature of the studied complexes, the bond length of each of the six C_(tris-NHC)_→M_3_ bonds in each complex remains the same. Therefore, only one bond length for the C_(tris-NHC)_→M_3_ bond is reported. The bond lengths of the C_(tris-NHC)_→M_3_ bonds in the mentioned complexes are listed in the ESI, Table S1.†

### Interaction energy

The interaction energies between the examined M_3_^3+^ ions and the ligand fragments in the optimized structures of the nano-sized complexes were calculated using the PBE/def2-TZVP level of theory. The results are shown in Table 2. To calculate the interaction energy in the complexes [L_2_(R)_6_→M_3_]^3+^, three fragments A, A′, and B were defined as follows Fig. 4. In this study, the interaction energy between fragments A and B, as well as A and A′B, was investigated. Single point (SP) energy of all fragments, (A, B, A′, AB and A′B) were calculated using the PBE/def2-TZVP level of theory. Due to the symmetry of the complex, the interaction energy was analysed for only one side of the complex. The interaction energy was measured according to the following equations.^42^ The total interaction energy was calculated using two formulas, eqn (1) and eqn (2). The total interaction energies obtained from both formulas are same.1
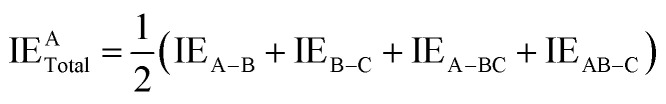

2 IE^B^_Total_ = *E*_ABC_ − (*E*^ABC^_A_ + *E*^ABC^_B_ + *E*^ABC^_C_)

**Table 2:** Interaction energy (IE) (kcal mol^−1^) of [L_2_(R)_6_→M_3_]^3+^ complexes

M	R	IE^ABA′^_A−B_	IE^ABA′^_B−A′_	IE^ABA′^_A−BA′_	IE^ABA'^_AB−A′_	IE_total(__eqn (1)__)_	IE_total(__eqn (2)__)_
Cu	C_2_H_5_	−360.60	−360.60	−272.42	−272.42	−633.02	−633.02
	CH_3_	−353.73	−353.73	−270.31	−270.31	−624.04	−624.04
	H	−341.57	−341.56	−267.69	−267.68	−609.25	−609.25
	F	−311.61	−311.62	−251.54	−251.55	−563.16	−563.16
	Cl	−330.02	−330.02	−259.21	−259.21	−589.23	−589.23
	Br	−336.10	−336.10	−261.11	−261.11	−598.11	−598.11
	SiH_3_	−358.89	−358.89	−271.66	−271.66	−630.55	−630.55
	Ph	−373.52	−373.09	−277.89	−277.46	−650.98	−650.98
Ag	C_2_H_5_	−318.43	−318.43	−244.87	−244.87	−563.30	−563.30
	CH_3_	−308.74	−308.74	−240.28	−240.27	−549.01	−549.01
	H	−305.49	−305.49	−247.25	−247.25	−552.74	−552.74
	F	−268.23	−268.23	−222.27	−222.27	−490.50	−490.50
	Cl	−289.92	−289.93	−232.30	−232.32	−522.24	−522.24
	Br	−293.81	−293.80	−232.19	−232.18	−525.99	−525.99
	SiH_3_	−312.10	−312.10	−242.03	−242.02	−554.12	−554.12
	Ph	−328.82	−328.82	−246.73	−246.73	−575.55	−575.55
Au	C_2_H_5_	−439.04	−438.66	−304.27	−303.89	−742.93	−742.93
	CH_3_	−429.20	−429.21	−301.16	−301.17	−730.37	−730.37
	H	−415.94	−415.95	−300.52	−300.53	−716.48	−716.48
	F	−380.23	−380.23	−284.79	−284.79	−665.02	−665.02
	Cl	−402.31	−402.32	−291.90	−291.90	−694.21	−694.21
	Br	−411.16	−411.16	−293.81	−293.81	−704.97	−704.97
	SiH_3_	−434.76	−434.79	−303.19	−303.22	−737.98	−737.98
	Ph	−449.84	−449.30	−307.12	−306.57	−756.41	−756.41

Results indicated that the interaction energy values for Cu(i), Ag(i) Au(i); in [L_2_(R)_6_→M_3_]^3+^ complexes were in the ranges of (−563.16 to −650.98) kcal mol^−1^, (−490.50 to −575.55) kcal mol^−1^ and (−665.02 to −756.41) kcal mol^−1^ respectively. Assuming R is constant, the trend of total interaction energy for group 11 metals is V-shaped, IE Au(1) > IE Cu(1) > IE Ag(1). For example, the total interaction energy for the complexes [L_2_(C_2_H_5_)_6_→Au_3_]^3+^, [L_2_(RC_2_H_5_)_6_→Ag_3_]^3+^ and [L_2_(C_2_H_5_)_6_→Cu_3_]^3+^ were −742.93 kcal mol^−1^, −563.30 kcal mol^−1^ and −633.02 kcal mol^−1^, respectively. Changing the substituent on the NHC rings alters the interaction energy. The total interaction energies for electron-donating substituents, such as Ph, C_2_H_5_, and CH_3_, are higher than those for electron-withdrawing substituents such as F, Cl, and Br. This trend was observed across all three metal clusters investigated. Additionally, the highest and lowest total interaction energies were associated with the complexes [L_2_(Ph)_6_→Au_3_]^3+^, which has an interaction energy of −756.41 kcal mol^−1^, and [L_2_(F)_6_→Ag_3_]^3+^, which has an interaction energy of −490.50 kcal mol^−1^.

### Cooperativity

In 1957 Frank and Wen^43^ qualitatively described the presence of cooperative effects among hydrogen bonds as a nonadditive enhancement of interactions through the formation of additional bonds. Significant attention has been focused on cooperativity within a triad formed by noncovalent interactions among three connected monomers^44^ Hankins^45^ and colleagues proposed a characteristic feature of the cooperative effect in a triadic configuration of H_2_O molecules, treating it as a noncyclic A–B–C system based on the concept of “pairwise nonadditivity” represented by the following equation: eqn (3)

3 *E*_coop_ = SE_ABC_ − SE_AB_ − SE_BC_ – SE_(AC, T)_

SE_ABC_ denotes the stabilization energy of the triad, while SE_AB_ and SE_BC_ represent the stabilization energies of the isolated dyads within their respective minima configurations. The term SE_(AC, T)_ also signifies the stabilization energy of molecules A and C in the triad configuration. However, while several theoretical studies^46–51^ have calculated the cooperative energy using eqn (3) and other studies have employed eqn (4), which does not take SE_(AC, T)_ into account.^52–56^

4 *E*_coop_ = SE_ABC_ − SE_AB_ − SE_BC_

Cooperativity refers to the phenomenon where multiple interactions within a system influence each other, causing the system to behave differently than would be expected from the individual interactions acting alone. This coupling can result in either positive cooperativity (where one interaction enhances another) or negative cooperativity (where one interaction reduces another). It is a fundamental aspect of systems chemistry that produces collective properties not found in the individual components.^57^ One example of cooperativity is hemoglobin, where the binding of oxygen to one site increases the affinity of the other sites for oxygen.^58^ In 2024, Sanei Movafagh and co-workers introduced a new equation eqn (5) for calculating cooperativity based on bond interaction energy in three-component systems.^55^5
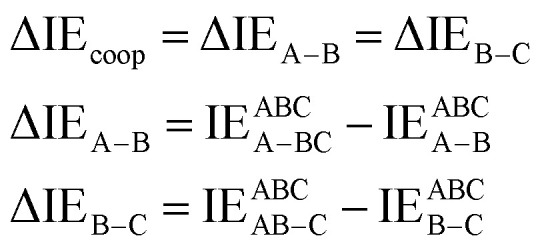


The calculated values of the cooperativity energy based on eqn (5) for the nano-sized complexes [L_2_(R)_6_→M_3_]^3+^; M = Cu(i), Ag(i), Au(i) and R = C_2_H_5_, CH_3_, H, F, Cl, Br, Ph, and SiH_3_) are summarized in Table 3. Based on the results, the cooperativity values for all complexes are positive, indicating the presence of anti-cooperativity. The trend of cooperativity for group 11 metals, similar to the interaction energy, exhibits a V-shaped pattern and follows the order, Ag(i) < Cu(i) < Au(i). For example, cooperativity energy for the complexes [L_2_(F)_6_→Cu_3_]^3+^, [L_2_(F)_6_→Ag_3_]^3+^ and [L_2_(F)_6_→Au_3_]^3+^ were 73.88 kcal mol^−1^, 58.24 kcal mol^−1^ and 115.42 kcal mol^−1^, respectively.

**Table 3:** Cooperativity energy (kcal mol^−1^) of [L_2_(R)_6_→M_3_]^3+^ complexes

M	R	IE^ABA′^_A−B_	IE^ABA′^_A−BA′_	*E*_coop_
Cu	C_2_H_5_	−360.60	−272.42	88.18
	CH_3_	−353.73	−270.31	83.42
	H	−341.57	−267.69	73.88
	F	−311.61	−251.54	60.07
	Cl	−330.02	−259.21	70.81
	Br	−336.1	−261.11	74.99
	SiH_3_	−358.89	−271.66	87.23
	Ph	−373.52	−277.89	95.63
Ag	C_2_H_5_	−318.43	−244.87	73.56
	CH_3_	−308.74	−240.28	68.46
	H	−305.49	−247.25	58.24
	F	−268.23	−222.27	45.96
	Cl	−289.92	−232.30	57.62
	Br	−293.81	−232.19	61.62
	SiH_3_	−312.1	−242.03	70.07
	Ph	−328.82	−246.73	82.09
Au	C_2_H_5_	−439.04	−304.27	134.77
	CH_3_	−429.2	−301.16	128.04
	H	−415.94	−300.52	115.42
	F	−380.23	−284.79	95.44
	Cl	−402.31	−291.9	110.41
	Br	−411.16	−293.81	117.35
	SiH_3_	−434.76	−303.19	131.57
	Ph	−449.84	−307.12	142.72

### NBO analysis

In this study, we used Gaussian 09 software for NBO analysis. NBO analysis was utilized to quantify various bond parameters and to characterize the metal–ligand interactions. Based on NBO calculation at the PBE-D3/def2-TZVP level of theory, the nature of the C_(tris-NHC)_→M_3_ bonds in the mentioned nano-sized complexes were analysed.

### Natural charge and charge transfer

The natural charge on M_3_ atoms of the complexes [L_2_(R)_6_→M_3_]^3+^; M = Cu(i), Ag(i), Au(i); R = C_2_H_5_, CH_3_, H, F, Cl, Br, Ph, and SiH_3_ at the PBE-D3/def2-TZVP level of theory were collected. The results were shown in Table 4. In the complexes [L_2_(R)_6_→M_3_]^3+^, the formal charges of the M_3_ and L_2_(R_6_) fragments are +3 and 0, respectively. The results indicate that charge transfer occurs from the L_2_(R)_6_ fragment to the M_3_. While the R substituent is constant, a decreasing trend in the natural charge was observed by changing the M from Cu(i) to Au(i). For example, the natural charges of the M_3_ metal cluster in the complexes [L_2_(Br)_6_→Cu_3_]^3+^, [L_2_(Br)_6_→Ag_3_]^3+^ and [L_2_(Br)_6_→Au_3_]^3+^ were found to be 1.17*e*, 1.04*e*, and 0.65*e*, respectively. Additionally, the natural charge of the M_3_ metal cluster is generally higher in the presence of electron-withdrawing substituents compared to electron-donating substituents. For example, the natural charges of complexes [L_2_(F)_6_→Ag_3_]^3+^ and [L_2_(C_2_H_5_)_6_→Ag_3_]^3+^ were 1.21 e and 1.04 respectively. The highest and lowest natural charges of the mentioned complexes correspond to the [L_2_(F)_6_→Cu_3_]^3+^ and [L_2_(C_2_H_5_)_6_→Au_3_]^3+^, with values of 1.25 e and 0.61 e, respectively. The amount of charge transfer from L_2_(R_6_) fragment to M_3_ was also calculated. The results indicate an increasing trend in charge transfer when changing the M_3_ from Cu(i) to Au(i) while keeping the same R substituent. For instance, the charge transfer amounts of [L_2_(Br)_6_→Cu_3_]^3+^, [L_2_(Br)_6_→Ag_3_]^3+^, and [L_2_(Br)_6_→Au_3_]^3+^ complexes are −1.83*e*, −1.96*e* and −2.35*e*, respectively.

**Table 4:** Natural charge and the amount of charge transfer L_2_(R)_6_→M_3_ for [L_2_(R)_6_→M_3_]^3+^ complexes

M	R	Natural charge		Charge transfer
		M_3_	L_2_(R)_6_	L_2_(R)_6_→Cu_3_
Cu	C_2_H_5_	1.09	1.91	−1.91
	CH_3_	1.09	1.91	−1.91
	H	1.14	1.86	−1.86
	F	1.25	1.75	−1.75
	Cl	1.20	1.80	−1.80
	Br	1.17	1.83	−1.83
	SiH_3_	1.15	1.85	−1.85
	Ph	1.16	1.84	−1.84
Ag	C_2_H_5_	1.05	1.95	−1.95
	CH_3_	1.05	1.95	−1.95
	H	1.11	1.89	−1.89
	F	1.21	1.79	−1.79
	Cl	1.10	1.90	−1.90
	Br	1.04	1.96	−1.96
	SiH_3_	1.09	1.91	−1.91
	Ph	1.09	1.91	−1.91
Au	C_2_H_5_	0.61	2.39	−2.39
	CH_3_	0.66	2.34	−2.34
	H	0.75	2.25	−2.25
	F	0.82	2.18	−2.18
	Cl	0.71	2.29	−2.29
	Br	0.65	2.35	−2.35
	SiH_3_	0.68	2.32	−2.32
	Ph	0.67	2.33	−2.33

The results confirm that changing the metal from Cu(i) to Au(i) leads to a reduction in the natural charge on the M_3_. Also the complexes with electron-donating substituents exhibit greater amounts of charge transfer compared to those with electron-withdrawing substituents. Specifically, the highest and lowest charge transfer values correspond to [L_2_(C_2_H_5_)_6_→Au_3_]^3+^ and [L_2_(F)_6_→Cu_3_]^3+^ complexes which have charge transfers about −2.39*e* and −1.75*e*, respectively.

### Wiberg index

Using the Wiberg bond index (WBI) method, the chemical bond orders of C_(tris-NHC)_→M_3_ in [L_2_(R)_6_→M_3_]^3+^ complexes; M = Cu(i), Ag(i), Au(i) and R = C_2_H_5_, CH_3_, H, F, Cl, Br, Ph and SiH_3_ are investigated. The results are summarized in Table S2 in the ESI.† Due to the symmetrical nature of the studied complexes, the bond order for each of the six C_(tris-NHC)_→M_3_ bonds are same. Therefore, only one bond order for the C_(tris-NHC)_→M_3_ bond is reported. By changing the M_3_ from Cu(i) to Au(i) while keeping the same R substituents, a well-known V-shaped trend emerges for the Wiberg Bond Index (WBI) values of the C(_tris-NHC_)→M_3_ bonds. This trend follows the order: Ag(i) < Cu(i) < Au(i), which correlates well with the interaction energies. The results indicate that varying the substituents (R) does not affect the Wiberg index for the corresponding complexes.

### Donor–acceptor and natural hybrid orbital (NHO) analysis

The results of the natural hybrid orbital (NHO) analysis for the M and C atoms in the C_(tris-NHC)_→M_3_ bond within the [L_2_(R)_6_→M_3_]^3+^ complexes; M = Cu(i), Ag(i), Au(i); R = C_2_H_5_, CH_3_, H, F, Cl, Br, Ph and SiH_3_ are reported in Table 5. Apart from that, the occupancy of C atom in the latter bonds was ≃73% in the presence of Au(i). For silver and copper, no occupancy of the C atoms in the latter bonds was observed. The modification of the R substituent does not significantly affect the occupancy values of carbon atoms in the C(_tris-NHC_)→M_3_ bond. The calculated donor–acceptor interactions for the investigated complexes are given in Table S3.† The results of indicate that the σ* and Lp* orbitals of M metal ion in the C_(tris-NHC)_→M_3_ bonds are filled with the lone-pair electrons from the carbon atoms. The most significant and strong donor–acceptor interactions for Cu(i) and Ag(i) are observed as C_NHC_ → Cu(i) and C_NHC_ → Ag(i) transitions, which occur from the lone pair (LP) to the lone pair antibonding orbital (LP*). In the case of Au(i), the strongest interaction is from C_NHC_ → Au(i)–C_NHC_, transitioning from the lone pair (LP) to the sigma antibonding orbital (σ*). These interactions highlight the varying nature of bonding in the metal complexes depending on the metal involved.

**Table 5:** Natural hybrid orbital (NHO) analysis of [L_2_(R)_6_→M_3_]^3+^ nano-sized complexes

Occupancy	C_2_H_5_		CH_3_		H		F		Cl		Br		SiH_3_		Ph	
	Au	C	Au	C	Au	C	Au	C	Au	C	Au	C	Au	C	Au	C
Occupancy Au–C	1.96140		1.96240		1.96259		1.95893		1.96252		1.96212		1.95799		1.96057	
%	26.98	73.02	27.17	72.83	27.03	72.97	26.16	73.84	26.42	73.58	26.51	73.49	27.08	72.92	26.54	73.46
% S	75.37	38.64	75.14	38.62	75.02	38.38	75.80	39.41	75.56	39.28	75.43	39.06	75.39	38.53	75.54	39.61
% p	4.37	61.33	4.34	61.35	3.80	61.59	3.90	60.57	4.13	60.69	4.24	60.91	4.32	61.44	4.62	60.36
% d	20.22	0.01	20.48	0.01	21.14	0.01	20.26	0.01	20.28	0.01	20.28	0.01	20.24	0.02	19.79	0.01
% f	0.04	0.01	0.03	0.02	0.04	0.02	0.04	0.02	0.04	0.02	0.04	0.02	0.05	0.02	0.05	0.01

### Energy decomposition analysis (EDA)

Energy decomposition analysis (EDA) is considered fundamental tool for obtaining information about the driving forces in molecular structure and for quantitatively interpreting chemical bonds. EDA calculations were performed based on the DFT method using the BP86-D3 functional and the TZ2P basis set on the relevant nano-sized complexes, utilizing ADF 2013 software. Four parameters are examined in the EDA calculations. The first parameter, Δ*E*_pauli_, corresponds to the repulsive interactions between fragments, based on the fact that two electrons with similar spins cannot occupy the same region in space, which typically results in positive values. The second parameter, Δ*E*_elstat_, determines the electrostatic interaction energy between the fragments, calculated using the frozen electron density distribution of the fragments in the molecular structure. The third parameter, Δ*E*_orb_, includes covalent attraction in the M-L bonds. The fourth parameter, Δ*E*_dis_, represents the change in energy due to dispersion interactions in a molecular system, arising from the attractive forces between temporary dipoles that form in molecules. These calculations were conducted to analyze the nature of the C_(tris-NHC)_→M_3_ bond in the complexes [L_2_(R)_6_→M_3_]^3+^; (M = Cu(i), Ag(i), Au(i) and R = C_2_H_5_, CH_3_, H, F, Cl, Br, Ph and SiH_3_). The total interaction energy was calculated using the following equation eqn (6)

6 Δ*E*_int_ = Δ*E*_elstat_ + Δ*E*_orb_ + Δ*E*_Pauli_ +Δ*E*_disp_

Three fragments A, A′ and B have been previously defined for the complexes [L_2_(R)_6_→M_3_]^3+^. In this study, the interaction energies for the fragments A–B and A–BA′ in the complexes, [L_2_(R)_6_→M_3_]^3+^; M = Cu(i), Ag(i), Au(i) and R = C_2_H_5_, CH_3_, H, F, Cl, Br, Ph and SiH_3_, were calculated using the ADF 2013 software at the BP86-D3, TZ2P theoretical level. Optimized output files were used to create the input files for ADF 2013. The results of the energy decomposition analysis (EDA) for the mentioned complexes are presented in Table 6. Assuming that the M_3_ remains unchanged, the interaction energy increases as the substituents change from electron-withdrawing to electron-donating. For example, the interaction energy of the [L_2_(F)_6_→Au_3_]^3+^ and [L_2_(Ph)_6_→Au_3_]^3+^ complexes, for the fragments A–B and A–BA′ were (−461.36, −387.17) and (−371.51, −291.15) in kcal mol^−1^, respectively. However, if the substituent R remains unchanged, the interaction energy exhibits a V-shaped trend, following the order Ag(i) < Cu(i) < Au(i). For instance, the interaction energy of, the [L_2_(C_2_H_5_)_6_→Au_3_]^3+^, [L_2_(C_2_H_5_)_6_→Ag_3_]^3+^ and [L_2_(C_2_H_5_)_6_→Cu_3_]^3+^ complexes for the fragments A–B and A–BA′ were (−460.90, −313.57), (−317.88, −248.38) and (−394.96, −290.59) in kcal mol^−1^, respectively. On the other hand, the highest and lowest interaction energies for A–B fragments in the complexes investigated here correspond to the [L_2_(Ph)_6_→Au_3_]^3+^ and [L_2_(F)_6_→Ag_3_]^3+^ complexes and those for A–BA′ fragments are (−461.26, −317.51) and (−267.55, −225.16) in kcal mol^−1^, respectively. The computational results align with the interaction energies calculated using Gaussian, at the PBE-D3/def2-TZVP theoretical level. The EDA results indicated that among the three energy decomposition terms in the mentioned complexes. Δ*E*_elstat_ is the most significant energy term. The values of Δ*E*_elstat_ for the A–B fragment in the respective complexes of Cu(i), Ag(i) and Au(i) were observed in the ranges of (61.23–65.08) %, (63.78–67.72) %, and (61.53–64.33) %, respectively and for the fragment A–BA′ in ranges of (64.92–69.25) %, (67.71–71.93) % and (67.03–70.17) %, respectively. On the other hand the value of Δ*E*_orb_ in [L_2_(R)_6_→Cu_3_]^3+^, [L_2_(R)_6_→Ag_3_]^3+^ and [L_2_(R)_6_→Au_3_]^3+^ complexes for fragment A–B were (32.87–36.48)%, (30.05–33.14)% and (33.88–36.45)%, respectively, and for the fragment A–BA′ were (27.25–30.53)%, (23.81–28.38)% and (27.23–30.06)% respectively. According to the obtained results, the highest and lowest values of the Δ*E*_elstat_ in the studied complexes for fragment A–B correspond to complexes [L_2_(H)_6_→Ag_3_]^3+^, (67.72%) and [L_2_(Ph)_6_→Cu_3_]^3+^ (61.23%), respectively and for fragment A–BA and those correspond to complexes [L_2_(Ph)_6_→Ag_3_]^3+^, (71.93%) and [L_2_(Ph)_6_→Cu_3_]^3+^, (64.92%), respectively. The results indicated that the highest and lowest values of Δ*E*_orb_ for the A–B fragment in the aforesaid complexes correspond to complex [L_2_(Br)_6_→Cu_3_]^3+^ with)36.48% (and complex [L_2_(H)_6_→Ag_3_]^3^ with) 30.05% (, respectively. For the A–BA′ fragment, the highest and lowest values of Δ*E*_orb_ correspond to complex [L_2_(F)_6_→Cu_3_]^3+^ with (30.53%) and complex [L_2_(Ph)_6_→Ag_3_]^3+^ with (23.81%), respectively. The results of the energy decomposition analysis showed that in all studied complexes, the ΔE_elstat_ accounted for the largest share, indicating that the interaction between the fragments is predominantly electrostatic.

**Table 6:** Energy decomposition analysis (EDA), (kcal mol^−1^) of [L_2_(R)_6_→M_3_]^3+^ nano-sized complexes

M	R	Fragment	Δ*E*_Pauli_	Δ*E*_elstat_	Δ*E*_orb_	Δ*E*_Dis_	Δ*E*_int_
	C_2_H_5_	A–B	332.9	−462.6(63.6)%	−246.9(33.9)%	−18.4(2.6)%	−395.0
	C_2_H_5_	A–BA′	319.3	−413.7(67.8)%	−167.6(27.5)%	−28.6(4.7)%	−290.6
	CH_3_	A–B	328.3	−458.7(64.9)%	−239.1(33.9)%	−8.3(1.1)%	−378.1
	CH_3_	A–BA′	315.4	−412.0(68.3)%	−165.8(27.5)%	−25.8(4.2)%	−288.2
	H	A–B	326.6	−454.7(65.1)%	−229.6(32.9)%	−14.4(2.0)%	−372.1
	H	A–BA′	305.9	−408.2(69.3)%	−160.6(27.2)%	−20.6(3.5)%	−283.6
Cu	F	A–B	300.6	−396.2(61.8)%	−231.5(36.1)%	−13.7(2.1)%	−340.7
	F	A–BA′	275.2	−357.5(65.9)%	−165.5(30.5)%	−19.1(3.6)%	−266.9
	Cl	A–B	312.8	−416.8(61.9)%	−241.8(35.9)%	−14.9(2.2)%	−360.8
	Cl	A–BA′	290.3	−375.2(66.3)%	−168.5(29.8)%	−22.4(3.9)%	−275.6
	Br	A–B	314.4	−419.3(61.3)%	−249.2(36.5)%	−15.4(2.2)%	−369.5
	Br	A–BA′	294.0	−377.2(65.9)%	−171.3(29.9)%	−24.00(4.2)%	−278.5
	SiH_3_	A–B	334.3	−459.4(63.1)%	−249.0(34.2)%	−19.7(2.7)%	−393.7
	SiH_3_	A–BA′	322.9	−413.4(67.7)%	−169.7(27.8)%	−27.5(4.5)%	−287.8
	Ph	A–B	330.2	−451.9(61.2)%	−266.0(36.1)%	−20.1(2.7)%	−407.8
	Ph	A–BA′	326.1	−404.6(64.9)%	−177.1(28.4)%	−41.5(6.7)%	−297.2
	C_2_H_5_	A–B	366.8	−454.30(66.4)%	−212.6(31.0)%	−17.8(2.6)%	−317.9
	C_2_H_5_	A–BA′	349.2	−419.6(70.2)%	−153.6(25.7)%	−24.4(4.1)%	−248.4
	CH_3_	A–B	363.4	−449.0(66.8)%	−206.8(30.8)%	−16.4(2.4)%	−308.7
	CH_3_	A–BA′	345.4	−416.6(70.6)%	−151.7(25.7)%	−21.8(3.7)%	−244.8
	H	A–B	353.3	−439.4(67.7)%	−195.0(30.1)%	−14.4(2.2)%	−295.6
	H	A–BA′	332.4	−408.1(71.2)%	−146.9(25.6)%	−18.3(3.2)%	−240.9
Ag	F	A–B	328.1	−385.4(64.7)%	−195.3(32.8)%	−115.0(2.5)%	−267.6
	F	A–BA′	304.6	−360.8(68.1)%	−150.4(28.4)%	−18.6(3.5)%	−225.2
	Cl	A–B	344.7	−413.8 (65.1)%	−203.6(32.0)%	−18.40(2.9)%	−291.1
	Cl	A–BA′	320.7	−379.2(68.2)%	−153.5(27.6)%	−23.2(4.2)%	−235.4
	Br	A–B	344.4	−408.9(63.8)%	−212.5(33.1)%	−19.7(3.1)%	−296.8
	Br	A–BA′	324.1	−381.2(67.7)%	−156.4(27.8)%	−25.5(4.5)%	−238.9
	SiH_3_	A–B	368.7	−450.2(66.0)%	−212.9(31.2)%	−19.2(2.8)%	−313.5
	SiH_3_	A–BA′	351.8	−418.4(69.7)%	−154.9(25.8)%	−26.7(4.5)%	−248.3
	Ph	A–B	360.2	−442.8(64.1)%	−228.2(33.0)%	−20.1(2.9)%	−330.8
	Ph	A–BA′	655.5	−634.5(71.9)%	−210.1(23.8)%	−37.5(4.3)%	−226.6
	C_2_H_5_	A–B	553.3	−633.4(62.2)%	−347.9(34.2)%	−36.8(3.6)%	−460.9
	C_2_H_5_	A–BA′	526.0	−580.3(69.1)%	−228.8(27.3)%	−30.5(3.6)%	−313.6
	CH_3_	A–B	547.8	−626.5(63.5)%	−340.4(34.5)%	−20.0(2.0)%	−439.1
	CH_3_	A–BA′	519.5	−576.9(69.6)%	−226.7(27.3)%	−25.6(3.1)%	−309.8
	H	A–B	546.1	−623.8(64.3)%	−328.5(33.9)%	−17.4(1.8)%	−423.6
	H	A–BA′	509.3	−572.7(70.2)%	−222.3(27.2)%	−21.3(2.6)%	−306.9
	F	A–B	507.7	−551.4(61.5)%	−326.5(36.5)%	−17.9(2.0)%	−388.2
Au	F	A–BA′	463.5	−506.2(67.0)%	−226.9(30.1)%	−21.6(2.9)%	−291.2
	Cl	A–B	523.4	−575.8(61.5)%	−339.1(36.3)%	−20.1(2.2)%	−412.4
	Cl	A–BA′	488.4	−531.7(67.4)%	−231.1(29.3)%	−26.1(3.3)%	−300.5
	Br	A–B	549.6	−591.8(61.5)%	−344.7(35.0)%	−24.4(2.5)%	−411.3
	Br	A–BA′	491.2	−533.3(67.1)%	−233.0(29.2)%	−29.4(3.7)%	−304.5
	SiH_3_	A–B	555.1	−630.4(63.0)%	−347.9(34.8)%	−22.6(2.2)%	−445.9
	SiH_3_	A–BA′	531.4	−583.1(69.1)%	−230.9(27.3)%	−30.7(3.6)%	−313.3
	Ph	A–B	545.0	−619.2(61.5)%	−362.6(36.1)%	−24.4(2.4)%	−461.3
	Ph	A–BA′	523.0	−564.6(67.2)%	−234.4(27.9)%	−41.5(4.9)%	−317.5

### EDA-NOCV analysis

NOCV (Natural Orbital for Chemical Valence) provides insights into orbital interactions for asymmetric molecules, as the density of shape changes decomposes the chemical bond into various components (σ, π, δ). The NOCV method indicates the interactions between fragments, which, through energy decomposition calculations, relate to significant orbital interactions between the fragments. Moreover the Δ*E*_orb_ term can be further analyzed using the NOCV (Natural Orbital for Chemical Valence) extension of the EDA method. The EDA-NOCV approach offers pairwise energy contributions from each set of interacting orbitals to the total bond energy. Δ*ρ* refers to the change in electron density between the fragments L_2_(R)_6_ and M^3+^. The eigenvalues (*ν*) give the size of the charge migration. The NOCV analysis for the two fragments, A–B and A–BA′, was performed, and the results for the [L_2_(C_2_H_5_)_6_→Au_3_]^3+^ complex are presented in Fig. 5. The density of shape between the fragments M^3+^ and (L_2_(R)_6_), along with the significant energy results of the remaining complexes [L_2_(R)_6_→M_3_]^3+^; M = Cu(i), Ag(i), Au(i) and R = C_2_H_5_, CH_3_, H, F, Cl, Br, Ph and SiH_3_ for the fragments A–B and A–BA′, is summarized in Fig. S3–S5.† NOCV analysis showed that the types of interactions Δ*ρ*_1_, Δ*ρ*_2_ and Δ*ρ*_3_ present in the C_(tris-NHC)_→M_3_ bond in both fragments A–B and A–BA′ were of the σ type, arising from the stable non-bonding electron pairs on the carbon atom. It may be referred to donation from the non-bonding electron pair of carbon towards the empty d orbital of the metal ions.

## Conclusion

In this study, the effect of substituents including: C_2_H_5_, CH_3_, H, F, Cl, Br, Ph and SiH_3_ on the M–C bond in the nano-sized [L_2_(R)_6_→M_3_]^3+^; [M = Cu(i), Ag(i), Au(i)] complexes was investigated at the PBE-D3/def2-TZVP level of theory. The total interaction energies for all mentioned complexes for the fragments AB and A–BA′ were also calculated. The interaction energy values for both AB and A–BA′ fragments in group 11 metals followed a V-shaped pattern, the trend in energy change observed as (IE Au(i) > IE Cu(i) > IE Ag(i)). The cooperativity energy values for the complexes [L_2_(R)_6_→M_3_]^3+^; M = Cu(i), Ag(i), Au(i) and R = C_2_H_5_, CH_3_, H, F, Cl, Br, Ph and SiH_3_ were calculated. The results indicate that all complexes have positive cooperativity values, suggesting anti-cooperativity. The cooperativity trend for Cu(i), Ag(i), Au(i) metals showed a V-shaped pattern, with the order Ag(i) < Cu(i) < Au(i), similar to the trend observed in interaction energies. The natural charge and charge transfer amounts for the complexes [L_2_(R)_6_→M_3_]^3+^ with the mentioned substituents were calculated, showing that the charge transfer for the corresponding gold complexes is greater than that for silver, and silver is slightly greater than copper. It seems that in most cases, the complexes with electron-donating substituents have slightly more charge transfer compared to those with electron-withdrawing substituents. Energy decomposition analysis was performed for the two fragments AB and A–BA′ in the corresponding complexes. The results showed that in all studied complexes, the Δ*E*_elstat_ accounted for the largest share, indicating that the interaction between the fragments is predominantly electrostatic. The values of Δ*E*_elstat_ for fragment A–B in the respective complexes Cu(i), Ag(i) and Au(i) were observed in ranges (61.23–65.08)%, (63.78–67.72)% and (61.53–64.33)% respectively, and for the fragment A–BA′ in ranges (64.92–69.25)%, (67.71–71.93)% and (67.03–70.17)%, respectively.

## Data availability

The authors confirm that the data supporting the findings of this study are available within the article and its ESI.†

## Conflicts of interest

The authors have no conficts of interest to declare.

## Supplementary Material

RA-015-D4RA08514K-s001
