# Supplementary material for: Trinuclear cylinder-like potential anticancer and antibacterial Cu(i), Ag(i) and Au(i) nano-sized cationic complexes with tris-NHC ligands: cationic M3 metal cluster displaying positive or negative cooperativity in triad [L2(R)6→M3]3+ complexes?
Source: RSC Adv. 2025 Feb 28;15(9):6742–52. doi: 10.1039/d4ra08514k (PMC11869827; doi:10.1039/d4ra08514k)
Supplement: RA-015-D4RA08514K-s001 [file RA-015-D4RA08514K-s001.pdf]

Supplementary Information (SI) for RSC Advances.
This journal is © The Royal Society of Chemistry 2025

# Supplementary Information For:
# Trinuclear cylinder-like potential anticancer and antibacterial Cu(I), Ag(I) and Au(I) nano-sized cationic complexes with tris-NHC ligands: Cationic $M_3$ metal cluster displaying positive or negative cooperativity in triad $[L_2(R)_6 \rightarrow M_3]^{3+}$ complexes?

Khadijeh Naeimi[a], Mehdi Bayat,*[b] and Ehsan Alavipour[a]
[a] Department of Inorganic Chemistry, Faculty of Chemistry, Bu-Ali Sina University, 6517838695, Hamedan, Iran,
[b] School of Chemistry, College of Science, University of Tehran, Iran,
Email: bayatm@ut.ac.ir (mehdi806@gmail.com)

Table S1. The calculated M–C bond lengths (Å) of $[L_2(R)_6 \rightarrow M_3]^{3+}$; M=Cu(I), Ag(I), Au(I); R = $C_2H_5$, $CH_3$, H, F, Cl, Br, Ph, and $SiH_3$ at the PBE-D3/def2-TZVP level of theory.

| R | Cu(I) | Ag(I) | Au(I) |
|---|---|---|---|
| | M-C | M-C | M-C |
| $C_2H_5$ | 1.90 | 2.08 | 2.03 |
| $CH_3$ | 1.90 | 2.08 | 2.03 |
| H | 1.90 | 2.08 | 2.03 |
| F | 1.90 | 2.08 | 2.03 |
| Cl | 1.90 | 2.08 | 2.03 |
| Br | 1.90 | 2.08 | 2.03 |
| $SiH_3$ | 1.91 | 2.08 | 2.03 |
| Ph | 1.90 | 2.08 | 2.03 |

# Atomic coordinates

$[L_2(C_2H_5)_6\rightarrow Cu_3]^{3+}$

| | | | |
|---|---|---|---|
| Cu | 1.798949000 | 3.045512000 | 0.051471000 |
| Cu | -3.471650000 | -0.000054000 | 0.000001000 |
| N | 4.114083000 | 2.732506000 | -1.772538000 |
| N | 2.526980000 | 1.353906000 | -2.242575000 |
| N | -2.348237000 | 1.528227000 | -2.248344000 |
| N | -4.335615000 | 2.210325000 | -1.772009000 |
| N | -0.061507000 | -2.777076000 | -2.314022000 |
| N | 0.351033000 | -4.851141000 | -1.903422000 |
| C | 2.857956000 | 2.385174000 | -1.393506000 |
| C | 4.568107000 | 1.944916000 | -2.816331000 |
| H | 5.541964000 | 2.083833000 | -3.273324000 |
| C | 3.570401000 | 1.068703000 | -3.116764000 |
| H | 3.493116000 | 0.321372000 | -3.897926000 |
| C | 4.921519000 | 3.772980000 | -1.113461000 |
| H | 4.212661000 | 4.412334000 | -0.569085000 |
| H | 5.381957000 | 4.384029000 | -1.902736000 |
| C | 5.971679000 | 3.188368000 | -0.177939000 |
| H | 5.500781000 | 2.596780000 | 0.620068000 |
| H | 6.544456000 | 4.001616000 | 0.287051000 |
| H | 6.681719000 | 2.547005000 | -0.717808000 |
| C | 1.268540000 | 0.686270000 | -2.237589000 |
| C | 0.089711000 | 1.439017000 | -2.229088000 |
| H | 0.136885000 | 2.528113000 | -2.224081000 |
| C | -1.138644000 | 0.775319000 | -2.241197000 |
| C | -1.200876000 | -0.621740000 | -2.264960000 |
| H | -2.167480000 | -1.125245000 | -2.287680000 |
| C | -3.412052000 | 1.283980000 | -1.410353000 |
| C | -2.615933000 | 2.593070000 | -3.101728000 |
| H | -1.917896000 | 2.926517000 | -3.860808000 |
| C | -3.876087000 | 3.012890000 | -2.801709000 |
| H | -4.477160000 | 3.801395000 | -3.241635000 |
| C | -5.682661000 | 2.308582000 | -1.183244000 |
| H | -5.903668000 | 3.375413000 | -1.038421000 |
| H | -5.618324000 | 1.842819000 | -0.189339000 |
| C | -6.741926000 | 1.632659000 | -2.044612000 |
| H | -6.807086000 | 2.091054000 | -3.040703000 |
| H | -7.724957000 | 1.734219000 | -1.565773000 |
| H | -6.524931000 | 0.562379000 | -2.168375000 |
| C | 1.228797000 | -0.709174000 | -2.261740000 |
| H | 2.148475000 | -1.294107000 | -2.282720000 |
| C | -0.012126000 | -1.354073000 | -2.273256000 |
| C | 0.679678000 | -3.599030000 | -1.495880000 |
| C | -0.839651000 | -3.517511000 | -3.197448000 |
| H | -1.460506000 | -3.058779000 | -3.958201000 |
| C | -0.571965000 | -4.826166000 | -2.934626000 |
| H | -0.942708000 | -5.727987000 | -3.409806000 |
| C | 0.860866000 | -6.085799000 | -1.283114000 |
| H | 1.778338000 | -5.803985000 | -0.747942000 |
| H | 1.146267000 | -6.770128000 | -2.094624000 |
| C | -0.153060000 | -6.726920000 | -0.344681000 |
| H | -0.416628000 | -6.042990000 | 0.474644000 |
| H | 0.274314000 | -7.639653000 | 0.091148000 |
| H | -1.072607000 | -7.009771000 | -0.875077000 |
| Cu | 1.799070000 | -3.045448000 | -0.051484000 |
| N | 4.114181000 | -2.732370000 | 1.772542000 |
| N | 2.527028000 | -1.353825000 | 2.242574000 |
| N | -2.348182000 | -1.528312000 | 2.248334000 |
| N | -4.335542000 | -2.210467000 | 1.772006000 |
| N | -0.061599000 | 2.777069000 | 2.314024000 |
| N | 0.350865000 | 4.851149000 | 1.903427000 |
| C | 2.858044000 | -2.385079000 | 1.393503000 |
| C | 4.568173000 | -1.944766000 | 2.816338000 |
| H | 5.542033000 | -2.083650000 | 3.273335000 |
| C | 3.570436000 | -1.068588000 | 3.116768000 |
| H | 3.493121000 | -0.321261000 | 3.897931000 |
| C | 4.921656000 | -3.772815000 | 1.113466000 |
| H | 4.212822000 | -4.412192000 | 0.569086000 |
| H | 5.382111000 | -4.383850000 | 1.902742000 |
| C | 5.971800000 | -3.188164000 | 0.177951000 |
| H | 5.500885000 | -2.596591000 | -0.620057000 |
| H | 6.544607000 | -4.001391000 | -0.287039000 |
| H | 6.681814000 | -2.546778000 | 0.717824000 |
| C | 1.268566000 | -0.686232000 | 2.237584000 |
| C | 0.089762000 | -1.439019000 | 2.229079000 |
| H | 0.136973000 | -2.528113000 | 2.224070000 |
| C | -1.138615000 | -0.775363000 | 2.241190000 |
| C | -1.200895000 | 0.621694000 | 2.264958000 |
| H | -2.167516000 | 1.125166000 | 2.287678000 |
| C | -3.412005000 | -1.284098000 | 1.410345000 |
| C | -2.615840000 | -2.593172000 | 3.101708000 |
| H | -1.917788000 | -2.926604000 | 3.860783000 |
| C | -3.875985000 | -3.013024000 | 2.801699000 |
| H | -4.477035000 | -3.801545000 | 3.241628000 |
| C | -5.682592000 | -2.308754000 | 1.183254000 |
| H | -5.903575000 | -3.375589000 | 1.038430000 |
| H | -5.618277000 | -1.842985000 | 0.189351000 |
| C | -6.741865000 | -1.632860000 | 2.044636000 |
| H | -6.807004000 | -2.091260000 | 3.040725000 |
| H | -7.724898000 | -1.734442000 | 1.565806000 |
| H | -6.524895000 | -0.562575000 | 2.168401000 |
| C | 1.228776000 | 0.709210000 | 2.261738000 |
| H | 2.148434000 | 1.294175000 | 2.282722000 |
| C | -0.012169000 | 1.354067000 | 2.273254000 |
| C | 0.679552000 | 3.599051000 | 1.495880000 |
| C | -0.839764000 | 3.517474000 | 3.197457000 |
| H | -1.460599000 | 3.058718000 | 3.958212000 |
| C | -0.572127000 | 4.826140000 | 2.934636000 |
| H | -0.942901000 | 5.727946000 | 3.409819000 |
| C | 0.860649000 | 6.085827000 | 1.283118000 |
| H | 1.778128000 | 5.804048000 | 0.747940000 |
| H | 1.146030000 | 6.770165000 | 2.094628000 |
| C | -0.153306000 | 6.726912000 | 0.344693000 |
| H | -0.416855000 | 6.042974000 | -0.474631000 |
| H | 0.274031000 | 7.639662000 | -0.091137000 |
| H | -1.072860000 | 7.009729000 | 0.875095000 |

$[L_2(CH_3)_6\rightarrow Cu_3]^{3+}$

| | | | |
|---|---|---|---|
| Cu | 2.002895000 | -2.891005000 | 0.000239000 |
| Cu | -3.505260000 | -0.289199000 | 0.000879000 |
| Cu | 1.501607000 | 3.180290000 | -0.001142000 |
| C | 3.000701000 | -2.134309000 | 1.442889000 |
| C | 4.655009000 | -1.553774000 | 2.884018000 |
| H | 5.633037000 | -1.611941000 | 3.349706000 |
| C | 3.589296000 | -0.754977000 | 3.164434000 |
| H | 3.449345000 | -0.002394000 | 3.931729000 |
| C | 5.151115000 | -3.389282000 | 1.244076000 |
| H | 6.079174000 | -2.922894000 | 0.890758000 |
| H | 4.626731000 | -3.845644000 | 0.397188000 |
| H | 5.390094000 | -4.161886000 | 1.986117000 |
| C | 1.276682000 | -0.554660000 | 2.255356000 |
| C | 0.158461000 | -1.394731000 | 2.256404000 |
| H | 0.287870000 | -2.477085000 | 2.272147000 |
| C | -1.117062000 | -0.826474000 | 2.255782000 |
| C | -1.285539000 | 0.561955000 | 2.256527000 |
| H | -2.287613000 | 0.991039000 | 2.272214000 |
| C | -0.155629000 | 1.382421000 | 2.255542000 |
| C | 1.131022000 | 0.834139000 | 2.256118000 |
| H | 2.003528000 | 1.487598000 | 2.271182000 |
| C | -3.347523000 | -1.529786000 | 1.444841000 |
| C | -2.446935000 | -2.728032000 | 3.167009000 |
| H | -1.724967000 | -2.982564000 | 3.934257000 |
| C | -3.671920000 | -3.251318000 | 2.887679000 |
| H | -4.211437000 | -4.068531000 | 3.354457000 |
| C | -5.510687000 | -2.763449000 | 1.249054000 |
| H | -5.642896000 | -2.084140000 | 0.399614000 |
| H | -5.573156000 | -3.801591000 | 0.899798000 |
| H | -6.298427000 | -2.579633000 | 1.990906000 |
| C | 0.349473000 | 3.665558000 | 1.443186000 |
| C | -1.136290000 | 3.485277000 | 3.167338000 |
| H | -1.716816000 | 2.987490000 | 3.935383000 |
| C | -0.977244000 | 4.807716000 | 2.887480000 |
| H | -1.414639000 | 5.683696000 | 3.354530000 |
| C | 0.362065000 | 6.155571000 | 1.246163000 |
| H | -0.506221000 | 6.727100000 | 0.895346000 |
| H | 1.017390000 | 5.929986000 | 0.397553000 |
| H | 0.913773000 | 6.747657000 | 1.987580000 |

| | | | |
|---|---|---|---|
| C | 0.943996000 | -3.558541000 | -1.442773000 |
| C | -0.179180000 | -4.903520000 | -2.885037000 |
| H | -0.467433000 | -5.839582000 | -3.351400000 |
| C | -0.553464000 | -3.625225000 | -3.164914000 |
| H | -1.208701000 | -3.229803000 | -3.932378000 |
| C | 1.365767000 | -6.012543000 | -1.245474000 |
| H | 0.603743000 | -6.720185000 | -0.895925000 |
| H | 1.973968000 | -5.682325000 | -0.396039000 |
| H | 2.008513000 | -6.504549000 | -1.986740000 |
| C | 0.070289000 | -1.389645000 | -2.254716000 |
| C | -1.178767000 | -0.765349000 | -2.255613000 |
| H | -2.097088000 | -1.352662000 | -2.271024000 |
| C | -1.240107000 | 0.631948000 | -2.255061000 |
| C | -0.074934000 | 1.401505000 | -2.256424000 |
| H | -0.124401000 | 2.490426000 | -2.272511000 |
| C | 1.165772000 | 0.755942000 | -2.255689000 |
| C | 1.249685000 | -0.637911000 | -2.255822000 |
| H | 2.217501000 | -1.139517000 | -2.271156000 |
| C | -3.555306000 | 0.960238000 | -1.443181000 |
| C | -2.864267000 | 2.289454000 | -3.166054000 |
| H | -2.194201000 | 2.658852000 | -3.933681000 |
| C | -4.158463000 | 2.604584000 | -2.886395000 |
| H | -4.824964000 | 3.322055000 | -3.353088000 |
| C | -5.891387000 | 1.822052000 | -1.246469000 |
| H | -5.910588000 | 1.127978000 | -0.398900000 |
| H | -6.121865000 | 2.835201000 | -0.894263000 |
| H | -6.639168000 | 1.514448000 | -1.988665000 |
| C | 2.607987000 | 2.597110000 | -1.445068000 |
| C | 3.413258000 | 1.332944000 | -3.167284000 |
| H | 3.397926000 | 0.567478000 | -3.934437000 |
| C | 4.333572000 | 2.296038000 | -2.888073000 |
| H | 5.288228000 | 2.514029000 | -3.354865000 |
| C | 4.522974000 | 4.188689000 | -1.248905000 |
| H | 3.932439000 | 4.551816000 | -0.400441000 |
| H | 5.516158000 | 3.881887000 | -0.898055000 |
| H | 4.629339000 | 4.990440000 | -1.990891000 |
| N | 4.277050000 | -2.378365000 | 1.838079000 |
| N | 2.581906000 | -1.125976000 | 2.280758000 |
| N | -2.264525000 | -1.671059000 | 2.282164000 |
| N | -4.197327000 | -2.512414000 | 1.841369000 |
| N | -0.313329000 | 2.798526000 | 2.281654000 |
| N | -0.076006000 | 4.892922000 | 1.839957000 |
| N | 0.725278000 | -4.839277000 | -1.838776000 |
| N | 0.146819000 | -2.812380000 | -2.280460000 |
| N | -2.510493000 | 1.276997000 | -2.281135000 |
| N | -4.555063000 | 1.789713000 | -1.839745000 |
| N | 2.359698000 | 1.533451000 | -2.282303000 |
| N | 3.826463000 | 3.047518000 | -1.841703000 |

$[L_2(H)_6\rightarrow Cu_3]^{3+}$

| | | | |
|---|---|---|---|
| Cu | 2.702507000 | -2.222446000 | -0.001203000 |
| Cu | -3.276030000 | -1.229225000 | -0.000748000 |
| Cu | 0.573863000 | 3.451403000 | 0.002006000 |
| C | 3.441972000 | -1.273698000 | 1.480545000 |
| C | 4.896970000 | -0.344528000 | 2.974083000 |
| H | 5.856196000 | -0.170836000 | 3.449339000 |
| C | 3.658867000 | 0.141125000 | 3.264470000 |
| H | 3.325186000 | 0.802463000 | 4.055525000 |
| H | 5.489361000 | -1.700463000 | 1.448349000 |
| C | 1.373408000 | -0.216246000 | 2.328457000 |
| C | 0.505123000 | -1.312104000 | 2.328848000 |
| H | 0.904157000 | -2.326615000 | 2.341795000 |
| C | -0.872367000 | -1.083880000 | 2.327358000 |
| C | -1.387268000 | 0.216008000 | 2.329219000 |
| H | -2.465377000 | 0.377689000 | 2.342420000 |
| C | -0.500891000 | 1.294853000 | 2.328831000 |
| C | 0.882297000 | 1.090805000 | 2.330546000 |
| H | 1.561406000 | 1.943587000 | 2.344791000 |
| C | -2.823126000 | -2.345601000 | 1.479446000 |
| C | -1.705049000 | -3.242760000 | 3.261618000 |
| H | -0.964916000 | -3.285300000 | 4.052105000 |
| C | -2.745004000 | -4.071751000 | 2.971197000 |
| H | -3.073956000 | -4.989730000 | 3.445806000 |
| H | -4.216608000 | -3.905116000 | 1.446748000 |
| C | -0.618695000 | 3.615808000 | 1.482924000 |
| C | -1.954364000 | 3.094375000 | 3.264825000 |
| H | -2.361084000 | 2.473918000 | 4.054818000 |
| C | -2.152662000 | 4.409681000 | 2.975478000 |
| H | -2.783328000 | 5.153016000 | 3.450728000 |
| H | -1.272996000 | 5.602235000 | 1.451878000 |
| C | 1.913120000 | -3.130206000 | -1.482878000 |
| C | 1.280935000 | -4.736664000 | -2.976420000 |
| H | 1.295243000 | -5.711204000 | -3.452043000 |
| C | 0.565220000 | -3.615465000 | -3.265798000 |
| H | -0.148729000 | -3.415401000 | -4.056195000 |
| H | 2.727050000 | -5.056742000 | -1.452074000 |
| C | 0.475579000 | -1.304317000 | -2.329045000 |
| C | -0.901699000 | -1.074757000 | -2.329934000 |
| H | -1.607361000 | -1.905707000 | -2.343576000 |
| C | -1.368918000 | 0.243004000 | -2.327898000 |
| C | -0.481464000 | 1.320972000 | -2.328884000 |
| H | -0.848224000 | 2.347593000 | -2.341726000 |
| C | 0.893366000 | 1.066688000 | -2.328083000 |
| C | 1.383219000 | -0.240834000 | -2.330112000 |
| H | 2.455666000 | -0.436489000 | -2.343829000 |
| C | -3.668448000 | -0.089929000 | -1.480760000 |
| C | -3.415773000 | 1.322025000 | -3.262230000 |
| H | -2.885999000 | 1.841180000 | -4.052353000 |
| C | -4.744452000 | 1.262446000 | -2.972174000 |
| H | -5.595861000 | 1.737835000 | -3.446786000 |
| H | -5.743825000 | 0.168406000 | -1.448466000 |
| C | 1.755525000 | 3.223595000 | -1.479228000 |
| C | 2.850304000 | 2.301054000 | -3.262916000 |
| H | 3.034272000 | 1.583692000 | -4.054134000 |
| C | 3.463306000 | 3.481158000 | -2.971931000 |
| H | 4.300252000 | 3.981415000 | -3.446709000 |
| H | 3.016921000 | 4.891757000 | -1.446068000 |
| N | 4.731583000 | -1.191236000 | 1.895103000 |
| N | 2.782474000 | -0.439776000 | 2.349794000 |
| N | -1.770475000 | -2.192420000 | 2.348021000 |
| N | -3.396331000 | -3.504015000 | 1.893280000 |
| N | -1.012009000 | 2.626827000 | 2.350753000 |
| N | -1.335499000 | 4.690940000 | 1.897705000 |
| N | 2.080735000 | -4.411347000 | -1.898022000 |
| N | 0.966835000 | -2.643750000 | -2.351120000 |
| N | -2.774523000 | 0.487314000 | -2.348877000 |
| N | -4.862001000 | 0.405926000 | -1.894670000 |
| N | 1.807670000 | 2.161908000 | -2.348752000 |
| N | 2.781328000 | 4.009840000 | -1.893111000 |

$[L_2(F)_6\rightarrow Cu_3]^{3+}$

| | | | |
|---|---|---|---|
| Cu | -0.749139000 | -3.393667000 | -0.000150000 |
| Cu | -2.564677000 | 2.345595000 | 0.000238000 |
| Cu | 3.314366000 | 1.048100000 | 0.000352000 |
| C | 0.367850000 | -3.615028000 | 1.529477000 |
| C | 1.795277000 | -4.592471000 | 3.056867000 |
| H | 2.339961000 | -5.417072000 | 3.504075000 |
| C | 1.676413000 | -3.271740000 | 3.383993000 |
| H | 2.100949000 | -2.717524000 | 4.213719000 |
| F | 0.856417000 | -5.910777000 | 1.300801000 |
| C | 0.400369000 | -1.329740000 | 2.460488000 |
| C | -0.963595000 | -1.022663000 | 2.461790000 |
| H | -1.706402000 | -1.820961000 | 2.464611000 |
| C | -1.351635000 | 0.318126000 | 2.460810000 |
| C | -0.403711000 | 1.345848000 | 2.461880000 |
| H | -0.723681000 | 2.388289000 | 2.464907000 |
| C | 0.951461000 | 1.011489000 | 2.460525000 |
| C | 1.367510000 | -0.323287000 | 2.461483000 |
| H | 2.430273000 | -0.567409000 | 2.463933000 |
| C | -3.314623000 | 1.488768000 | 1.529956000 |
| C | -3.671401000 | 0.183802000 | 3.384435000 |
| H | -3.403608000 | -0.460934000 | 4.214150000 |
| C | -4.874692000 | 0.741112000 | 3.057386000 |
| H | -5.861142000 | 0.681586000 | 3.504620000 |
| F | -5.547133000 | 2.213383000 | 1.301412000 |
| C | 2.947039000 | 2.126091000 | 1.529975000 |
| C | 1.995089000 | 3.087436000 | 3.384428000 |
| H | 1.302716000 | 3.177883000 | 4.214047000 |

| | | | |
|---|---|---|---|
| C | 3.079509000 | 3.850788000 | 3.057630000 |
| H | 3.521216000 | 4.734738000 | 3.505041000 |
| F | 4.691046000 | 3.697023000 | 1.301925000 |
| C | -1.855346000 | -3.124289000 | -1.529953000 |
| C | -3.561105000 | -3.410331000 | -3.057825000 |
| H | -4.402098000 | -3.929165000 | -3.505251000 |
| C | -2.897380000 | -2.262278000 | -3.384792000 |
| H | -3.049001000 | -1.580921000 | -4.214603000 |
| F | -3.264597000 | -5.001345000 | -1.301690000 |
| C | -0.922886000 | -1.037563000 | -2.460710000 |
| C | -1.376676000 | 0.282420000 | -2.461887000 |
| H | -2.443478000 | 0.508215000 | -2.464932000 |
| C | -0.437527000 | 1.318176000 | -2.460524000 |
| C | 0.932502000 | 1.051211000 | -2.461202000 |
| H | 1.661442000 | 1.862183000 | -2.463519000 |
| C | 1.359881000 | -0.279974000 | -2.460156000 |
| C | 0.443648000 | -1.332963000 | -2.461305000 |
| H | 0.781564000 | -2.369718000 | -2.463737000 |
| C | -1.778480000 | 3.168987000 | -1.529599000 |
| C | -0.510611000 | 3.640714000 | -3.384135000 |
| H | 0.155505000 | 3.431522000 | -4.213810000 |
| C | -1.173112000 | 4.789468000 | -3.057150000 |
| H | -1.201857000 | 5.777273000 | -3.504436000 |
| F | -2.699529000 | 5.327932000 | -1.301243000 |
| C | 3.633569000 | -0.044416000 | -1.529765000 |
| C | 3.407390000 | -1.377945000 | -3.384463000 |
| H | 2.892861000 | -1.849938000 | -4.214108000 |
| C | 4.733573000 | -1.378913000 | -3.057824000 |
| H | 5.603203000 | -1.848007000 | -3.505417000 |
| F | 5.963758000 | -0.326842000 | -1.301991000 |
| N | 0.993384000 | -4.730048000 | 1.949696000 |
| N | 0.808403000 | -2.698796000 | 2.458305000 |
| N | -2.741293000 | 0.649180000 | 2.458730000 |
| N | -4.593003000 | 1.504449000 | 1.950248000 |
| N | 1.933086000 | 2.049361000 | 2.458585000 |
| N | 3.599837000 | 3.225254000 | 1.950494000 |
| N | -2.891865000 | -3.872583000 | -1.950469000 |
| N | -1.869146000 | -2.107775000 | -2.458801000 |
| N | -0.891169000 | 2.672791000 | -2.458438000 |
| N | -1.908258000 | 4.440827000 | -1.949998000 |
| N | 2.759812000 | -0.564510000 | -2.458405000 |
| N | 4.799664000 | -0.568187000 | -1.950516000 |

$[L_2(Cl)_6 \rightarrow Cu_3]^{3+}$

| | | | |
|---|---|---|---|
| Cu | 1.601181000 | -3.085293000 | 0.000006000 |
| Cu | -3.472140000 | 0.156016000 | -0.000048000 |
| Cu | 1.871362000 | 2.928472000 | -0.000092000 |
| C | 2.641433000 | -2.509938000 | 1.491834000 |
| C | 4.368380000 | -2.266583000 | 2.986582000 |
| H | 5.323249000 | -2.506786000 | 3.440603000 |
| C | 3.421793000 | -1.339418000 | 3.300024000 |
| H | 3.387087000 | -0.618367000 | 4.109078000 |
| Cl | 4.695943000 | -4.210534000 | 1.121198000 |
| C | 1.175115000 | -0.740245000 | 2.387497000 |
| C | -0.055908000 | -1.403248000 | 2.388614000 |
| H | -0.091673000 | -2.492947000 | 2.393642000 |
| C | -1.229109000 | -0.646691000 | 2.388181000 |
| C | -1.187784000 | 0.750907000 | 2.389438000 |
| H | -2.113559000 | 1.326799000 | 2.395150000 |
| C | 0.054034000 | 1.388590000 | 2.388135000 |
| C | 1.243735000 | 0.654047000 | 2.388575000 |
| H | 2.205329000 | 1.167925000 | 2.393602000 |
| C | -3.494659000 | -1.031916000 | 1.492297000 |
| C | -2.871619000 | -2.292371000 | 3.301104000 |
| H | -2.230037000 | -2.622558000 | 4.110448000 |
| C | -4.147852000 | -2.648512000 | 2.987557000 |
| H | -4.833506000 | -3.355046000 | 3.441755000 |
| Cl | -5.994695000 | -1.960712000 | 1.121519000 |
| C | 0.853587000 | 3.542747000 | 1.491708000 |
| C | -0.549840000 | 3.634341000 | 3.300217000 |
| H | -1.156778000 | 3.244227000 | 4.109615000 |
| C | -0.220001000 | 4.917492000 | 2.986121000 |
| H | -0.489054000 | 5.864775000 | 3.439851000 |
| Cl | 1.299620000 | 6.172057000 | 1.119890000 |
| C | 0.531565000 | -3.605146000 | -1.491451000 |
| C | -0.661325000 | -4.877488000 | -2.986095000 |
| H | -1.014248000 | -5.796646000 | -3.440190000 |
| C | -0.874882000 | -3.569800000 | -3.299575000 |
| H | -1.444446000 | -3.126525000 | -4.108767000 |
| Cl | 0.740340000 | -6.264035000 | -1.120967000 |
| C | -0.071483000 | -1.387616000 | -2.387252000 |
| C | -1.250816000 | -0.640662000 | -2.388664000 |
| H | -2.224716000 | -1.130819000 | -2.394286000 |
| C | -1.166099000 | 0.754970000 | -2.387896000 |
| C | 0.070441000 | 1.402849000 | -2.389071000 |
| H | 0.132893000 | 2.491347000 | -2.394998000 |
| C | 1.236731000 | 0.631673000 | -2.387715000 |
| C | 1.179516000 | -0.763158000 | -2.388272000 |
| H | 2.090968000 | -1.361495000 | -2.393369000 |
| C | -3.387938000 | 1.341604000 | -1.492123000 |
| C | -2.654385000 | 2.541558000 | -3.300620000 |
| H | -1.985811000 | 2.813076000 | -4.109921000 |
| C | -3.893630000 | 3.010486000 | -2.987076000 |
| H | -4.513235000 | 3.775646000 | -3.441200000 |
| Cl | -5.794853000 | 2.490447000 | -1.121381000 |
| C | 2.855872000 | 2.262462000 | -1.492010000 |
| C | 3.528172000 | 1.026991000 | -3.300393000 |
| H | 3.428904000 | 0.312221000 | -4.109671000 |
| C | 4.554011000 | 1.865615000 | -2.986876000 |
| H | 5.526462000 | 2.019512000 | -3.441037000 |
| Cl | 5.054392000 | 3.772327000 | -1.121402000 |
| N | 3.864176000 | -2.952255000 | 1.893244000 |
| N | 2.380587000 | -1.504625000 | 2.391300000 |
| N | -2.493855000 | -1.308375000 | 2.392138000 |
| N | -4.489256000 | -1.869427000 | 1.893829000 |
| N | 0.113433000 | 2.814744000 | 2.391640000 |
| N | 0.625652000 | 4.823053000 | 1.892622000 |
| N | 0.189621000 | -4.859692000 | -1.892774000 |
| N | -0.140539000 | -2.813334000 | -2.390906000 |
| N | -2.366294000 | 1.527588000 | -2.391749000 |
| N | -4.303505000 | 2.264914000 | -1.893505000 |
| N | 2.506007000 | 1.284674000 | -2.391571000 |
| N | 4.113326000 | 2.593510000 | -1.893376000 |

$[L_2(Br)_6 \rightarrow Cu_3]^{3+}$

| | | | |
|---|---|---|---|
| Cu | 0.326897000 | -3.461141000 | -0.000313000 |
| Cu | -3.161669000 | 1.447548000 | -0.000166000 |
| Cu | 2.834238000 | 2.013733000 | 0.000060000 |
| C | 1.525388000 | -3.313044000 | 1.476418000 |
| C | 3.234140000 | -3.711998000 | 2.952084000 |
| H | 4.039622000 | -4.279358000 | 3.404557000 |
| C | 2.698832000 | -2.499803000 | 3.266643000 |
| H | 2.940094000 | -1.812782000 | 4.070084000 |
| Br | 2.832295000 | -5.795857000 | 1.026309000 |
| C | 0.820526000 | -1.121698000 | 2.365960000 |
| C | -0.568115000 | -1.285285000 | 2.366355000 |
| H | -1.002005000 | -2.285460000 | 2.371463000 |
| C | -1.381127000 | -0.150404000 | 2.365027000 |
| C | -0.828489000 | 1.133976000 | 2.365886000 |
| H | -1.477768000 | 2.009803000 | 2.370376000 |
| C | 0.560820000 | 1.270550000 | 2.365779000 |
| C | 1.396875000 | 0.149774000 | 2.367157000 |
| H | 2.479995000 | 0.274190000 | 2.372910000 |
| C | -3.631865000 | 0.335043000 | 1.476403000 |
| C | -3.513212000 | -1.088540000 | 3.265995000 |
| H | -3.038390000 | -1.641170000 | 4.069026000 |
| C | -4.830835000 | -0.946045000 | 2.952187000 |
| H | -5.724634000 | -1.360236000 | 3.404951000 |
| Br | -6.435645000 | 0.444620000 | 1.027787000 |
| C | 2.106397000 | 2.976878000 | 1.477106000 |
| C | 0.814765000 | 3.585995000 | 3.267087000 |
| H | 0.099022000 | 3.451135000 | 4.070359000 |
| C | 1.596932000 | 4.655811000 | 2.953009000 |
| H | 1.685365000 | 5.636964000 | 3.405727000 |
| Br | 3.603241000 | 5.350067000 | 1.028041000 |
| C | -0.878085000 | -3.539023000 | -1.477069000 |
| C | -2.482202000 | -4.250095000 | -2.952834000 |
| H | -3.167457000 | -4.957932000 | -3.405406000 |

| | | | |
|---|---|---|---|
| C | -2.182727000 | -2.959223000 | -3.267321000 |
| H | -2.547922000 | -2.329347000 | -4.070820000 |
| Br | -1.698113000 | -6.222106000 | -1.026826000 |
| C | -0.595388000 | -1.254346000 | -2.366260000 |
| C | -1.399770000 | -0.113360000 | -2.366320000 |
| H | -2.487023000 | -0.194167000 | -2.370571000 |
| C | -0.788555000 | 1.144200000 | -2.365595000 |
| C | 0.601760000 | 1.270226000 | -2.367150000 |
| H | 1.075415000 | 2.252189000 | -2.372393000 |
| C | 1.385272000 | 0.112084000 | -2.366619000 |
| C | 0.799313000 | -1.154995000 | -2.367609000 |
| H | 1.412752000 | -2.056283000 | -2.373052000 |
| C | -2.626568000 | 2.530634000 | -1.476528000 |
| C | -1.471272000 | 3.371579000 | -3.265778000 |
| H | -0.742641000 | 3.373497000 | -4.068779000 |
| C | -2.439998000 | 4.275896000 | -2.951627000 |
| H | -2.710341000 | 5.223368000 | -3.403999000 |
| Br | -4.541615000 | 4.581353000 | -1.027453000 |
| C | 3.504967000 | 1.009790000 | -1.476883000 |
| C | 3.655635000 | -0.409813000 | -3.267234000 |
| H | 3.293016000 | -1.041207000 | -4.070708000 |
| C | 4.923174000 | -0.023508000 | -2.952578000 |
| H | 5.878924000 | -0.262925000 | -3.404967000 |
| Br | 6.238652000 | 1.641500000 | -1.026546000 |
| N | 2.503571000 | -4.172738000 | 1.869016000 |
| N | 1.659751000 | -2.275633000 | 2.368791000 |
| N | -2.800054000 | -0.300313000 | 2.368111000 |
| N | -4.865205000 | -0.082585000 | 1.869463000 |
| N | 1.140490000 | 2.574276000 | 2.369086000 |
| N | 2.361549000 | 4.253802000 | 1.870050000 |
| N | -1.678427000 | -4.566292000 | -1.869783000 |
| N | -1.203807000 | -2.544931000 | -2.369452000 |
| N | -1.602108000 | 2.316348000 | -2.368291000 |
| N | -3.116153000 | 3.737390000 | -1.869100000 |
| N | 2.807172000 | 0.230546000 | -2.369388000 |
| N | 4.794855000 | 0.830549000 | -1.869449000 |

$[L_2(SiH_3)_6 \rightarrow Cu_3]^{3+}$

| | | | |
|---|---|---|---|
| Cu | -2.803866000 | 2.125068000 | -0.000441000 |
| Cu | 3.242362000 | 1.365305000 | 0.000078000 |
| Cu | -0.438502000 | -3.490345000 | 0.000284000 |
| C | -3.508788000 | 1.087130000 | 1.440471000 |
| C | -4.913746000 | 0.018634000 | 2.864789000 |
| H | -5.860572000 | -0.246949000 | 3.324052000 |
| C | -3.655145000 | -0.401255000 | 3.162466000 |
| H | -3.292884000 | -1.074132000 | 3.931222000 |
| Si | -6.186441000 | 1.847864000 | 1.060967000 |
| H | -7.313276000 | 0.886038000 | 0.966507000 |
| H | -5.643032000 | 2.265730000 | -0.261067000 |
| H | -6.478362000 | 3.006555000 | 1.943042000 |
| C | -1.384709000 | 0.130048000 | 2.270965000 |
| C | -0.584365000 | 1.276719000 | 2.271886000 |
| H | -1.045291000 | 2.264529000 | 2.283337000 |
| C | 0.804504000 | 1.133584000 | 2.271125000 |
| C | 1.397422000 | -0.132837000 | 2.273091000 |
| H | 2.483347000 | -0.227441000 | 2.285654000 |
| C | 0.579094000 | -1.264103000 | 2.271913000 |
| C | -0.814123000 | -1.144298000 | 2.272775000 |
| H | -1.439137000 | -2.037353000 | 2.284928000 |
| C | 2.695737000 | 2.494349000 | 1.441323000 |
| C | 1.479584000 | 3.365364000 | 3.163053000 |
| H | 0.715532000 | 3.388117000 | 3.931617000 |
| C | 2.472698000 | 4.245294000 | 2.865669000 |
| H | 2.716105000 | 5.197993000 | 3.325078000 |
| Si | 4.693800000 | 4.432655000 | 1.062534000 |
| H | 4.785484000 | 3.751492000 | -0.258564000 |
| H | 4.423739000 | 5.889211000 | 0.965947000 |
| H | 5.842560000 | 4.107749000 | 1.946056000 |
| C | 0.812525000 | -3.581961000 | 1.441390000 |
| C | 2.174501000 | -2.964720000 | 3.163567000 |
| H | 2.576094000 | -2.314632000 | 3.932388000 |
| C | 2.440366000 | -4.264547000 | 2.865602000 |
| H | 3.143911000 | -4.951701000 | 3.324711000 |
| Si | 1.492777000 | -6.280968000 | 1.061065000 |
| H | 2.889127000 | -6.776089000 | 0.966965000 |
| H | 0.859680000 | -6.018645000 | -0.261089000 |
| H | 0.634806000 | -7.113331000 | 1.942475000 |
| C | -1.994743000 | 3.084175000 | -1.441304000 |
| C | -1.345207000 | 4.725582000 | -2.865428000 |
| H | -1.345103000 | 5.708968000 | -3.324659000 |
| C | -0.600901000 | 3.627207000 | -3.163012000 |
| H | 0.144981000 | 3.460226000 | -3.931621000 |
| Si | -3.450331000 | 5.457033000 | -1.061860000 |
| H | -2.826730000 | 6.800528000 | -0.962046000 |
| H | -3.709913000 | 4.817638000 | 0.257805000 |
| H | -4.642689000 | 5.429577000 | -1.946920000 |
| C | -0.499265000 | 1.297649000 | -2.271501000 |
| C | 0.881841000 | 1.092704000 | -2.272907000 |
| H | 1.572728000 | 1.935826000 | -2.285009000 |
| C | 1.373699000 | -0.216294000 | -2.271748000 |
| C | 0.505682000 | -1.309928000 | -2.273032000 |
| H | 0.890283000 | -2.329873000 | -2.285285000 |
| C | -0.873897000 | -1.081348000 | -2.271593000 |
| C | -1.386983000 | 0.217190000 | -2.272673000 |
| H | -2.462585000 | 0.394062000 | -2.284394000 |
| C | 3.668492000 | 0.185535000 | -1.441155000 |
| C | 3.442244000 | -1.292453000 | -3.163465000 |
| H | 2.924858000 | -1.854755000 | -3.932308000 |
| C | 4.765585000 | -1.196947000 | -2.865735000 |
| H | 5.617315000 | -1.688396000 | -3.325047000 |
| Si | 6.450901000 | 0.260616000 | -1.061393000 |
| H | 6.027651000 | 0.797526000 | 0.261587000 |
| H | 7.305966000 | -0.949400000 | -0.969126000 |
| H | 7.019051000 | 1.313056000 | -1.941976000 |
| C | -1.673431000 | -3.269482000 | -1.440693000 |
| C | -2.840573000 | -2.334456000 | -3.162736000 |
| H | -3.068949000 | -1.605217000 | -3.931525000 |
| C | -3.419702000 | -3.528113000 | -2.864731000 |
| H | -4.271439000 | -4.019856000 | -3.323716000 |
| Si | -2.999979000 | -5.716494000 | -1.060591000 |
| H | -2.321635000 | -5.619220000 | 0.261560000 |
| H | -4.475377000 | -5.851089000 | -0.966437000 |
| H | -2.374412000 | -6.734885000 | -1.942283000 |
| N | -4.813546000 | 0.922969000 | 1.811732000 |
| N | -2.803463000 | 0.268454000 | 2.287191000 |
| N | 1.633835000 | 2.292964000 | 2.287765000 |
| N | 3.206020000 | 3.706320000 | 1.812791000 |
| N | 1.168693000 | -2.561935000 | 2.288285000 |
| N | 1.607218000 | -4.629819000 | 1.812396000 |
| N | -2.189090000 | 4.384801000 | -1.812555000 |
| N | -1.015805000 | 2.626242000 | -2.287848000 |
| N | 2.782615000 | -0.433079000 | -2.288047000 |
| N | 4.892126000 | -0.296189000 | -1.812451000 |
| N | -1.766238000 | -2.193021000 | -2.287625000 |
| N | -2.702686000 | -4.088147000 | -1.811621000 |

$[L_2(Ph)_6 \rightarrow Cu_3]^{3+}$

| | | | |
|---|---|---|---|
| Cu | 3.343361000 | -1.062772000 | -0.047510000 |
| Cu | -2.627738000 | -2.301047000 | 0.056956000 |
| Cu | -0.743766000 | 3.392307000 | -0.046688000 |
| C | 3.690847000 | 0.173099000 | 1.360869000 |
| C | 4.712816000 | 1.625256000 | 2.784027000 |
| H | 5.540961000 | 2.185403000 | 3.202905000 |
| C | 3.393477000 | 1.604736000 | 3.115877000 |
| H | 2.851915000 | 2.113812000 | 3.904587000 |
| C | 1.401486000 | 0.364597000 | 2.262281000 |
| C | 1.032200000 | -0.984042000 | 2.270161000 |
| H | 1.798152000 | -1.759749000 | 2.260043000 |
| C | -0.323932000 | -1.315316000 | 2.286700000 |
| C | -1.305963000 | -0.319195000 | 2.302412000 |
| H | -2.360856000 | -0.592686000 | 2.324299000 |
| C | -0.913727000 | 1.019680000 | 2.292566000 |
| C | 0.438508000 | 1.374888000 | 2.274123000 |
| H | 0.728086000 | 2.425829000 | 2.268623000 |
| C | -1.684769000 | -3.194735000 | 1.453205000 |
| C | -0.174282000 | -3.672139000 | 3.098148000 |

| | | | |
|---|---|---|---|
| H | 0.573559000 | -3.463682000 | 3.854287000 |
| C | -0.825306000 | -4.825214000 | 2.785723000 |
| H | -0.749743000 | -5.817427000 | 3.215303000 |
| C | -1.929281000 | 3.089676000 | 1.414412000 |
| C | -2.947955000 | 2.132398000 | 3.220982000 |
| H | -3.083294000 | 1.417361000 | 4.024126000 |
| C | -3.638589000 | 3.262100000 | 2.906719000 |
| H | -4.519618000 | 3.703733000 | 3.358244000 |
| C | 2.845414000 | -2.290730000 | -1.419207000 |
| C | 2.718485000 | -4.153611000 | -2.717665000 |
| H | 3.048503000 | -5.101608000 | -3.126467000 |
| C | 1.664271000 | -3.362066000 | -3.053984000 |
| H | 0.901749000 | -3.483677000 | -3.814400000 |
| C | 0.852803000 | -1.127854000 | -2.287903000 |
| C | -0.522677000 | -1.368075000 | -2.279931000 |
| H | -0.914375000 | -2.385184000 | -2.259649000 |
| C | -1.400262000 | -0.279920000 | -2.292563000 |
| C | -0.922317000 | 1.030991000 | -2.314527000 |
| H | -1.609164000 | 1.877135000 | -2.328222000 |
| C | 0.459133000 | 1.248932000 | -2.320526000 |
| C | 1.353858000 | 0.177714000 | -2.312776000 |
| H | 2.430318000 | 0.348421000 | -2.323982000 |
| C | -3.431433000 | -1.345913000 | -1.383085000 |
| C | -3.713156000 | 0.036138000 | -3.179943000 |
| H | -3.411208000 | 0.694406000 | -3.986248000 |
| C | -4.934288000 | -0.462965000 | -2.846040000 |
| H | -5.913079000 | -0.292603000 | -3.279680000 |
| C | 0.510142000 | 3.563081000 | -1.473293000 |
| C | 1.958249000 | 3.059200000 | -3.165723000 |
| H | 2.408198000 | 2.453014000 | -3.943331000 |
| C | 2.133891000 | 4.370197000 | -2.846072000 |
| H | 2.773822000 | 5.125342000 | -3.287722000 |
| N | 4.871952000 | 0.755349000 | 1.713420000 |
| N | 2.784480000 | 0.710115000 | 2.241503000 |
| N | -0.717879000 | -2.682930000 | 2.284608000 |
| N | -1.739643000 | -4.515380000 | 1.787549000 |
| N | -1.905441000 | 2.044290000 | 2.303888000 |
| N | -3.008922000 | 3.825123000 | 1.804725000 |
| N | 3.424983000 | -3.485997000 | -1.725901000 |
| N | 1.761489000 | -2.222889000 | -2.261140000 |
| N | -2.806473000 | -0.516066000 | -2.280736000 |
| N | -4.743407000 | -1.294171000 | -1.750305000 |
| N | 0.955486000 | 2.583122000 | -2.327025000 |
| N | 1.245122000 | 4.658336000 | -1.818926000 |
| C | -0.260289000 | 7.677934000 | -0.252088000 |
| C | 0.884819000 | 8.405540000 | 0.080862000 |
| C | 2.149818000 | 7.896308000 | -0.224596000 |
| C | 2.277337000 | 6.655908000 | -0.851430000 |
| C | 1.124433000 | 5.938622000 | -1.179619000 |
| C | -0.146796000 | 6.444225000 | -0.893637000 |
| H | -1.251657000 | 8.077241000 | -0.034590000 |
| H | 0.792960000 | 9.376454000 | 0.568869000 |
| H | 3.045155000 | 8.464021000 | 0.030987000 |
| H | 3.264984000 | 6.244433000 | -1.066325000 |
| H | -1.039082000 | 5.896517000 | -1.201113000 |
| C | 6.194705000 | 0.620517000 | -0.341277000 |
| C | 7.409206000 | 0.371909000 | -0.982904000 |
| C | 8.540239000 | 0.030104000 | -0.236024000 |
| C | 8.457174000 | -0.066975000 | 1.155653000 |
| C | 7.244752000 | 0.167307000 | 1.807072000 |
| C | 6.122408000 | 0.508406000 | 1.049914000 |
| H | 5.310963000 | 0.925500000 | -0.905126000 |
| H | 7.478694000 | 0.466506000 | -2.067390000 |
| H | 9.491257000 | -0.149712000 | -0.738803000 |
| H | 9.339020000 | -0.331337000 | 1.740387000 |
| H | 7.168722000 | 0.069461000 | 2.891235000 |
| C | -4.323534000 | -7.282110000 | -0.069786000 |
| C | -2.998168000 | -7.635356000 | 0.197614000 |
| C | -2.136253000 | -6.724787000 | 0.810608000 |
| C | -2.620073000 | -5.462832000 | 1.163848000 |
| C | -3.948088000 | -5.103718000 | 0.916917000 |
| C | -4.795121000 | -6.016888000 | 0.288400000 |
| H | -4.991584000 | -7.999598000 | -0.547462000 |
| H | -2.628934000 | -8.624019000 | -0.077118000 |
| H | -1.093829000 | -6.989165000 | 0.995956000 |
| H | -4.325823000 | -4.133971000 | 1.244918000 |
| H | -5.833348000 | -5.740303000 | 0.100968000 |
| C | -4.287647000 | 7.351733000 | -0.121595000 |
| C | -4.106647000 | 7.338467000 | 1.264097000 |
| C | -3.679193000 | 6.175562000 | 1.907579000 |
| C | -3.445330000 | 5.027063000 | 1.148936000 |
| C | -3.633890000 | 5.023478000 | -0.235830000 |
| C | -4.049647000 | 6.195417000 | -0.869987000 |
| H | -4.626304000 | 8.261927000 | -0.617988000 |
| H | -4.294055000 | 8.238954000 | 1.850139000 |
| H | -3.513292000 | 6.166584000 | 2.986085000 |
| H | -3.480476000 | 4.101292000 | -0.799657000 |
| H | -4.209889000 | 6.200241000 | -1.948948000 |
| C | -7.840163000 | -3.372955000 | 0.217381000 |
| C | -6.945614000 | -2.592644000 | 0.955434000 |
| C | -5.923297000 | -1.896799000 | 0.308007000 |
| C | -5.798261000 | -2.003316000 | -1.079808000 |
| C | -6.684679000 | -2.780215000 | -1.828163000 |
| C | -7.710954000 | -3.461691000 | -1.171331000 |
| H | -8.646877000 | -3.903691000 | 0.724417000 |
| H | -7.058332000 | -2.507736000 | 2.037075000 |
| H | -5.239986000 | -1.252207000 | 0.864276000 |
| H | -6.564915000 | -2.865218000 | -2.909464000 |
| H | -8.409126000 | -4.068241000 | -1.749333000 |
| C | 5.678390000 | -3.130459000 | -0.844091000 |
| C | 4.604215000 | -3.992444000 | -1.081894000 |
| C | 4.658197000 | -5.335145000 | -0.700207000 |
| C | 5.805636000 | -5.815708000 | -0.067604000 |
| C | 6.879697000 | -4.959804000 | 0.190632000 |
| C | 6.813233000 | -3.619114000 | -0.196167000 |
| H | 5.642585000 | -2.097456000 | -1.193766000 |
| H | 3.807895000 | -5.995417000 | -0.878928000 |
| H | 5.856947000 | -6.863739000 | 0.229493000 |
| H | 7.773795000 | -5.342939000 | 0.683637000 |
| H | 7.654995000 | -2.949399000 | -0.016060000 |

$[L_2(C_2H_5)_6\rightarrow Ag_3]^{3+}$

| | | | |
|---|---|---|---|
| Ag | 0.831131000 | -3.364301000 | -0.000312000 |
| Ag | 2.498753000 | 2.401613000 | 0.000555000 |
| Ag | -3.329605000 | 0.961986000 | -0.000355000 |
| C | -0.362351000 | -3.652578000 | -1.683065000 |
| C | -1.741084000 | -4.578690000 | -3.225221000 |
| H | -2.293229000 | -5.373503000 | -3.714941000 |
| C | -1.632247000 | -3.258402000 | -3.535922000 |
| H | -2.051934000 | -2.681363000 | -4.351694000 |
| C | -0.784789000 | -6.114622000 | -1.469982000 |
| H | -1.761390000 | -6.618443000 | -1.477298000 |
| H | -0.514103000 | -5.929938000 | -0.421097000 |
| C | 0.275762000 | -6.947964000 | -2.178457000 |
| H | 1.254092000 | -6.447957000 | -2.150212000 |
| H | 0.010332000 | -7.132503000 | -3.228261000 |
| H | 0.370052000 | -7.922643000 | -1.681327000 |
| C | -0.383228000 | -1.335328000 | -2.564386000 |
| C | 0.975607000 | -1.008757000 | -2.566377000 |
| H | 1.729471000 | -1.795806000 | -2.578857000 |
| C | 1.349310000 | 0.336652000 | -2.564063000 |
| C | 0.387066000 | 1.350154000 | -2.565390000 |
| H | 0.691726000 | 2.396566000 | -2.577073000 |
| C | -0.964942000 | 1.001057000 | -2.563629000 |
| C | -1.361575000 | -0.339018000 | -2.565927000 |
| H | -2.420110000 | -0.598371000 | -2.578186000 |
| C | 3.345823000 | 1.512536000 | -1.681994000 |
| C | 3.639369000 | 0.216310000 | -3.535289000 |
| H | 3.349476000 | -0.435346000 | -4.351318000 |
| C | 4.837303000 | 0.781699000 | -3.224126000 |
| H | 5.801777000 | 0.700771000 | -3.713679000 |
| C | 5.689446000 | 2.377055000 | -1.468269000 |
| H | 5.393774000 | 2.519362000 | -0.419526000 |
| H | 6.613771000 | 1.782732000 | -1.475139000 |
| C | 5.881870000 | 3.712030000 | -2.176777000 |
| H | 6.678956000 | 4.280614000 | -1.679390000 |
| H | 4.960025000 | 4.309820000 | -2.148934000 |
| H | 6.174723000 | 3.574181000 | -3.226457000 |
| C | -2.982180000 | 2.141260000 | -1.681874000 |
| C | -2.005231000 | 3.045257000 | -3.533749000 |

| | | | |
|---|---|---|---|
| H | -1.295467000 | 3.120714000 | -4.349309000 |
| C | -3.094089000 | 3.799665000 | -3.222649000 |
| H | -3.506014000 | 4.675761000 | -3.711720000 |
| C | -4.902802000 | 3.738454000 | -1.467893000 |
| H | -4.850157000 | 4.836106000 | -1.473675000 |
| H | -4.878813000 | 3.410229000 | -0.419448000 |
| C | -6.154802000 | 3.238464000 | -2.177599000 |
| H | -6.181210000 | 3.562046000 | -3.226977000 |
| H | -7.045965000 | 3.644138000 | -1.680321000 |
| H | -6.211813000 | 2.141211000 | -2.150879000 |
| C | 2.021440000 | -3.064596000 | 1.682670000 |
| C | 3.672293000 | -3.242760000 | 3.225313000 |
| H | 4.530841000 | -3.689259000 | 3.715164000 |
| C | 2.961161000 | -2.125071000 | 3.536129000 |
| H | 3.063693000 | -1.419216000 | 4.352128000 |
| C | 3.541375000 | -5.046970000 | 1.469692000 |
| H | 4.640228000 | -5.039109000 | 1.478906000 |
| H | 3.217687000 | -5.008666000 | 0.420269000 |
| C | 2.988474000 | -6.278186000 | 2.176505000 |
| H | 3.307451000 | -6.318793000 | 3.226839000 |
| H | 3.358860000 | -7.184726000 | 1.679487000 |
| H | 1.889885000 | -6.290159000 | 2.146349000 |
| C | 0.960722000 | -1.004396000 | 2.564321000 |
| C | 1.362794000 | 0.332811000 | 2.566163000 |
| H | 2.420357000 | 0.596090000 | 2.578611000 |
| C | 0.387934000 | 1.334184000 | 2.563881000 |
| C | -0.971166000 | 1.013820000 | 2.565474000 |
| H | -1.727884000 | 1.798138000 | 2.577339000 |
| C | -1.350987000 | -0.331107000 | 2.563806000 |
| C | -0.393939000 | -1.347885000 | 2.565980000 |
| H | -0.694789000 | -2.395384000 | 2.578146000 |
| C | 1.642585000 | 3.282629000 | 1.682802000 |
| C | 0.357480000 | 3.627194000 | 3.535066000 |
| H | -0.305802000 | 3.363266000 | 4.350538000 |
| C | 0.970212000 | 4.801784000 | 3.224532000 |
| H | 0.927237000 | 5.768664000 | 3.714138000 |
| C | 2.599626000 | 5.590102000 | 1.470119000 |
| H | 2.729636000 | 5.290041000 | 0.421024000 |
| H | 2.043071000 | 6.537632000 | 1.478105000 |
| C | 3.941469000 | 5.727779000 | 2.178420000 |
| H | 3.815835000 | 6.024851000 | 3.228448000 |
| H | 4.541611000 | 6.501794000 | 1.681673000 |
| H | 4.501575000 | 4.782590000 | 2.149420000 |
| C | -3.665333000 | -0.219787000 | 1.681746000 |
| C | -3.321728000 | -1.503812000 | 3.534921000 |
| H | -2.761821000 | -1.945580000 | 4.350974000 |
| C | -4.645125000 | -1.561145000 | 3.223673000 |
| H | -5.461066000 | -2.081796000 | 3.713149000 |
| C | -6.141939000 | -0.545504000 | 1.467931000 |
| H | -5.946849000 | -0.285427000 | 0.418253000 |
| H | -6.684878000 | -1.500872000 | 1.478084000 |
| C | -6.931385000 | 0.549893000 | 2.173654000 |
| H | -7.125987000 | 0.295122000 | 3.224280000 |
| H | -7.901669000 | 0.682157000 | 1.676580000 |
| H | -6.392177000 | 1.507091000 | 2.142416000 |
| N | -0.968302000 | -4.793224000 | -2.096270000 |
| N | -0.777389000 | -2.706362000 | -2.586833000 |
| N | 2.733803000 | 0.680574000 | -2.586232000 |
| N | 4.636707000 | 1.557905000 | -2.094956000 |
| N | -1.955071000 | 2.028063000 | -2.585535000 |
| N | -3.666699000 | 3.236826000 | -2.094338000 |
| N | 3.088618000 | -3.792081000 | 2.096014000 |
| N | 1.947859000 | -2.034275000 | 2.586778000 |
| N | 0.786241000 | 2.704024000 | 2.586262000 |
| N | 1.738788000 | 4.570658000 | 2.095986000 |
| N | -2.736406000 | -0.671252000 | 2.585954000 |
| N | -4.828913000 | -0.780725000 | 2.094548000 |

$[L_2(CH_3)_6\rightarrow Ag_3]^{3+}$

| | | | |
|---|---|---|---|
| Ag | -3.444197000 | -0.523787000 | 0.001132000 |
| Ag | 2.175735000 | -2.720348000 | 0.000025000 |
| Ag | 1.268612000 | 3.245027000 | -0.000490000 |
| C | -3.616330000 | 0.696621000 | -1.681183000 |
| C | -4.388039000 | 2.164543000 | -3.229018000 |
| H | -5.123055000 | 2.786111000 | -3.729069000 |
| C | -3.078785000 | 1.937120000 | -3.518894000 |
| H | -2.455587000 | 2.301447000 | -4.327350000 |
| C | -6.016597000 | 1.381162000 | -1.492486000 |
| H | -6.314324000 | 2.398059000 | -1.207859000 |
| H | -5.975673000 | 0.749803000 | -0.598574000 |
| H | -6.750449000 | 0.969728000 | -2.197322000 |
| C | -1.294883000 | 0.507628000 | -2.535289000 |
| C | -1.098011000 | -0.876055000 | -2.536556000 |
| H | -1.952628000 | -1.552333000 | -2.552642000 |
| C | 0.206266000 | -1.374923000 | -2.535363000 |
| C | 1.306115000 | -0.512544000 | -2.537479000 |
| H | 2.319077000 | -0.914543000 | -2.554311000 |
| C | 1.086067000 | 0.866448000 | -2.536066000 |
| C | -0.210708000 | 1.387759000 | -2.537335000 |
| H | -0.369156000 | 2.465995000 | -2.554238000 |
| C | 1.203520000 | -3.479785000 | -1.681365000 |
| C | -0.140920000 | -3.634784000 | -3.518066000 |
| H | -0.768508000 | -3.277336000 | -4.326197000 |
| C | 0.316511000 | -4.882385000 | -3.228006000 |
| H | 0.145030000 | -5.829850000 | -3.727556000 |
| C | 1.809859000 | -5.900946000 | -1.491985000 |
| H | 2.336001000 | -5.549874000 | -0.597957000 |
| H | 1.077882000 | -6.667096000 | -1.207464000 |
| H | 2.533164000 | -6.330914000 | -2.196664000 |
| C | 2.411353000 | 2.782129000 | -1.682457000 |
| C | 3.216359000 | 1.695329000 | -3.519807000 |
| H | 3.219899000 | 0.973240000 | -4.328067000 |
| C | 4.068753000 | 2.714781000 | -3.229939000 |
| H | 4.974956000 | 3.039652000 | -3.729838000 |
| C | 4.205922000 | 4.516888000 | -1.493599000 |
| H | 5.235287000 | 4.265397000 | -1.209187000 |
| H | 3.639088000 | 4.797412000 | -0.599527000 |
| H | 4.217100000 | 5.358249000 | -2.198296000 |
| C | -3.243803000 | -1.740729000 | 1.682824000 |
| C | -3.542368000 | -3.372740000 | 3.229957000 |
| H | -4.058638000 | -4.185244000 | 3.729906000 |
| C | -2.359966000 | -2.766040000 | 3.519290000 |
| H | -1.655945000 | -2.928804000 | 4.327106000 |
| C | -5.331690000 | -3.108633000 | 1.495098000 |
| H | -5.314087000 | -4.168355000 | 1.211550000 |
| H | -5.480485000 | -2.494579000 | 0.600582000 |
| H | -6.154357000 | -2.932978000 | 2.200073000 |
| C | -1.083224000 | -0.869747000 | 2.535927000 |
| C | 0.213778000 | -1.387254000 | 2.537517000 |
| H | 0.383539000 | -2.463781000 | 2.554151000 |
| C | 1.296608000 | -0.503607000 | 2.536018000 |
| C | 1.096361000 | 0.878419000 | 2.537654000 |
| H | 1.943827000 | 1.563626000 | 2.554299000 |
| C | -0.210333000 | 1.374319000 | 2.535757000 |
| C | -1.307020000 | 0.509829000 | 2.537294000 |
| H | -2.324133000 | 0.901206000 | 2.553768000 |
| C | 3.130762000 | -1.938714000 | 1.681069000 |
| C | 3.577880000 | -0.661522000 | 3.517920000 |
| H | 3.367280000 | 0.029006000 | 4.326330000 |
| C | 4.694466000 | -1.381695000 | 3.227298000 |
| H | 5.656622000 | -1.422627000 | 3.726524000 |
| C | 5.359666000 | -3.061919000 | 1.490824000 |
| H | 4.900906000 | -3.499149000 | 0.597676000 |
| H | 6.267230000 | -2.515753000 | 1.204876000 |
| H | 5.621563000 | -3.861262000 | 2.195854000 |
| C | 0.114672000 | 3.680551000 | 1.681136000 |
| C | -1.214659000 | 3.428445000 | 3.518087000 |
| H | -1.707117000 | 2.900500000 | 4.326460000 |
| C | -1.149849000 | 4.755579000 | 3.227596000 |
| H | -1.595844000 | 5.609051000 | 3.726914000 |
| C | -0.028232000 | 6.172331000 | 1.490951000 |
| H | 0.579129000 | 5.993765000 | 0.597320000 |
| H | -0.955335000 | 6.685005000 | 1.205680000 |
| H | 0.533446000 | 6.798924000 | 2.195583000 |
| N | -4.691284000 | 1.404452000 | -2.111097000 |
| N | -2.621959000 | 1.030793000 | -2.567182000 |
| N | 0.416614000 | -2.785796000 | -2.567151000 |
| N | 1.127214000 | -4.764790000 | -2.110701000 |
| N | 2.202902000 | 1.753877000 | -2.568308000 |
| N | 3.562430000 | 3.358063000 | -2.112240000 |

| | | | |
|---|---|---|---|
| N | -4.058981000 | -2.736734000 | 2.112796000 |
| N | -2.194421000 | -1.764195000 | 2.568096000 |
| N | 2.626833000 | -1.018784000 | 2.567258000 |
| N | 4.401301000 | -2.146552000 | 2.109944000 |
| N | -0.429590000 | 2.783855000 | 2.567269000 |
| N | -0.341025000 | 4.884562000 | 2.110195000 |

$[L_2(H)_6 \rightarrow Ag_3]^{3+}$

| | | | |
|---|---|---|---|
| Ag | 2.264565000 | 2.634679000 | 0.000198000 |
| Ag | -3.414059000 | 0.643694000 | 0.000707000 |
| Ag | 1.149812000 | -3.278310000 | -0.001158000 |
| C | 3.172151000 | 1.861652000 | -1.712878000 |
| C | 4.741710000 | 1.324774000 | -3.285261000 |
| H | 5.714885000 | 1.364472000 | -3.786413000 |
| C | 3.601487000 | 0.632045000 | -3.596831000 |
| H | 3.375684000 | -0.035848000 | -4.434104000 |
| H | 5.109716000 | 2.668041000 | -1.669026000 |
| C | 1.311618000 | 0.483967000 | -2.609447000 |
| C | 0.238516000 | 1.391630000 | -2.610639000 |
| H | 0.429696000 | 2.473842000 | -2.623698000 |
| C | -1.075266000 | 0.895703000 | -2.608654000 |
| C | -1.324861000 | -0.487449000 | -2.610649000 |
| H | -2.357701000 | -0.862911000 | -2.623738000 |
| C | -0.238482000 | -1.377247000 | -2.609387000 |
| C | 1.084155000 | -0.901766000 | -2.611540000 |
| H | 1.925731000 | -1.608501000 | -2.625347000 |
| C | -3.198535000 | 1.817604000 | -1.711432000 |
| C | -2.348437000 | 2.805341000 | -3.594845000 |
| H | -1.657187000 | 2.944194000 | -4.432094000 |
| C | -3.518428000 | 3.446285000 | -3.282796000 |
| H | -4.039416000 | 4.269539000 | -3.783418000 |
| H | -4.865620000 | 3.092431000 | -1.666660000 |
| C | 0.025242000 | -3.677492000 | -1.713429000 |
| C | -1.255909000 | -3.434234000 | -3.596248000 |
| H | -1.722092000 | -2.904631000 | -4.433080000 |
| C | -1.225804000 | -4.768109000 | -3.284918000 |
| H | -1.678376000 | -5.630690000 | -3.785849000 |
| H | -0.245122000 | -5.758671000 | -1.669657000 |
| C | 1.363775000 | 3.415710000 | 1.713232000 |
| C | 0.597148000 | 4.886542000 | 3.285837000 |
| H | 0.490189000 | 5.854556000 | 3.787142000 |
| C | 0.083566000 | 3.655141000 | 3.597189000 |
| H | -0.542826000 | 3.331421000 | 4.434405000 |
| H | 1.869892000 | 5.452442000 | 1.669707000 |
| C | 0.281084000 | 1.369195000 | 2.609492000 |
| C | -1.054775000 | 0.936246000 | 2.611238000 |
| H | -1.879840000 | 1.662182000 | 2.624701000 |
| C | -1.326210000 | -0.442793000 | 2.609232000 |
| C | -0.283298000 | -1.383179000 | 2.610633000 |
| H | -0.499366000 | -2.460697000 | 2.623563000 |
| C | 1.046703000 | -0.928738000 | 2.608941000 |
| C | 1.339669000 | 0.444648000 | 2.610999000 |
| H | 2.380867000 | 0.796263000 | 2.624143000 |
| C | -3.639886000 | -0.528092000 | 1.712915000 |
| C | -3.206993000 | -1.757604000 | 3.596118000 |
| H | -2.613410000 | -2.138636000 | 4.433114000 |
| C | -4.530171000 | -1.928546000 | 3.284627000 |
| H | -5.314945000 | -2.505594000 | 3.785569000 |
| H | -5.656728000 | -1.108396000 | 1.669006000 |
| C | 2.277428000 | -2.889245000 | 1.711354000 |
| C | 3.125636000 | -1.901010000 | 3.595363000 |
| H | 3.158849000 | -1.197005000 | 4.432841000 |
| C | 3.935220000 | -2.961285000 | 3.283195000 |
| H | 4.827289000 | -3.352789000 | 3.783929000 |
| H | 3.788325000 | -4.345774000 | 1.666536000 |
| N | 4.445780000 | 2.052336000 | -2.145725000 |
| N | 2.653957000 | 0.977807000 | -2.629912000 |
| N | -2.174086000 | 1.811306000 | -2.628526000 |
| N | -4.000489000 | 2.825547000 | -2.143596000 |
| N | -0.481922000 | -2.786669000 | -2.629871000 |
| N | -0.446784000 | -4.875768000 | -2.146060000 |
| N | 1.360939000 | 4.703456000 | 2.146291000 |
| N | 0.567745000 | 2.770477000 | 2.630114000 |
| N | -2.683057000 | -0.895264000 | 2.629604000 |
| N | -4.753595000 | -1.174880000 | 2.145534000 |
| N | 2.116950000 | -1.877650000 | 2.628762000 |
| N | 3.394314000 | -3.530747000 | 2.143644000 |

$[L_2(F)_6 \rightarrow Ag_3]^{3+}$

| | | | |
|---|---|---|---|
| Ag | 0.152088000 | -3.437926000 | 0.000380000 |
| Ag | 2.901386000 | 1.850840000 | -0.000568000 |
| Ag | -3.053760000 | 1.587237000 | 0.000223000 |
| C | -0.975374000 | -3.492788000 | -1.747565000 |
| C | -2.448627000 | -4.276288000 | -3.338740000 |
| H | -3.092160000 | -5.016988000 | -3.801000000 |
| C | -2.102727000 | -3.001026000 | -3.682683000 |
| H | -2.387699000 | -2.407796000 | -4.544486000 |
| F | -1.831739000 | -5.674271000 | -1.502302000 |
| C | -0.595975000 | -1.254238000 | -2.720430000 |
| C | 0.797968000 | -1.156422000 | -2.722225000 |
| H | 1.413772000 | -2.056210000 | -2.722740000 |
| C | 1.383047000 | 0.110789000 | -2.720701000 |
| C | 0.601381000 | 1.269077000 | -2.722712000 |
| H | 1.072716000 | 2.252272000 | -2.723569000 |
| C | -0.788595000 | 1.142140000 | -2.720692000 |
| C | -1.400873000 | -0.113939000 | -2.722390000 |
| H | -2.488020000 | -0.197305000 | -2.722953000 |
| C | 3.511785000 | 0.901610000 | -1.748695000 |
| C | 3.648843000 | -0.320988000 | -3.683596000 |
| H | 3.277241000 | -0.864543000 | -4.545160000 |
| C | 4.926341000 | 0.017131000 | -3.340200000 |
| H | 5.889398000 | -0.169947000 | -3.802777000 |
| F | 5.829285000 | 1.250699000 | -1.504333000 |
| C | -2.537587000 | 2.590254000 | -1.748292000 |
| C | -1.548132000 | 3.320016000 | -3.683721000 |
| H | -0.891905000 | 3.269909000 | -4.545517000 |
| C | -2.479663000 | 4.257263000 | -3.340128000 |
| H | -2.799441000 | 5.184726000 | -3.802740000 |
| F | -3.998772000 | 4.422603000 | -1.503738000 |
| C | 1.280215000 | -3.392167000 | 1.748199000 |
| C | 2.817124000 | -4.042211000 | 3.339277000 |
| H | 3.523602000 | -4.723108000 | 3.801579000 |
| C | 2.359995000 | -2.802413000 | 3.683054000 |
| H | 2.591523000 | -2.186202000 | 4.544746000 |
| F | 2.325875000 | -5.489527000 | 1.503152000 |
| C | 0.704614000 | -1.195764000 | 2.720724000 |
| C | 1.405487000 | 0.011266000 | 2.722438000 |
| H | 2.495745000 | 0.024372000 | 2.722996000 |
| C | 0.684521000 | 1.208283000 | 2.720641000 |
| C | -0.711231000 | 1.211768000 | 2.722627000 |
| H | -1.267698000 | 2.149415000 | 2.723213000 |
| C | -1.387385000 | -0.011123000 | 2.720984000 |
| C | -0.692517000 | -1.221623000 | 2.722908000 |
| H | -1.226300000 | -2.172371000 | 2.723831000 |
| C | 2.298579000 | 2.804922000 | 1.747585000 |
| C | 1.248741000 | 3.445141000 | 3.682884000 |
| H | 0.599621000 | 3.337581000 | 4.544806000 |
| C | 2.093855000 | 4.460860000 | 3.338850000 |
| H | 2.330560000 | 5.413088000 | 3.801114000 |
| F | 3.592248000 | 4.759031000 | 1.502197000 |
| C | -3.577612000 | 0.588227000 | 1.748760000 |
| C | -3.606201000 | -0.641084000 | 3.684064000 |
| H | -3.188081000 | -1.149434000 | 4.545801000 |
| C | -4.908560000 | -0.417184000 | 3.340589000 |
| H | -5.851342000 | -0.688402000 | 3.803253000 |
| F | -5.916877000 | 0.731295000 | 1.504375000 |
| N | -1.742862000 | -4.505586000 | -2.181509000 |
| N | -1.208862000 | -2.546278000 | -2.715495000 |
| N | 2.808452000 | 0.225969000 | -2.716207000 |
| N | 4.772477000 | 0.743211000 | -2.183058000 |
| N | -1.601111000 | 2.318924000 | -2.716123000 |
| N | -3.031058000 | 3.761101000 | -2.182703000 |
| N | 2.134219000 | -4.333141000 | 2.182196000 |
| N | 1.429347000 | -2.428553000 | 2.715902000 |
| N | 1.389828000 | 2.452288000 | 2.715615000 |
| N | 2.686775000 | 4.014922000 | 2.181522000 |
| N | -2.817374000 | -0.022385000 | 2.716469000 |
| N | -4.819393000 | 0.319304000 | 2.183236000 |

$[L_2(Cl)_6 \rightarrow Ag_3]^{3+}$

| | | | |
|---|---|---|---|
| Ag | -2.761675000 | -2.062341000 | -0.000031000 |
| Ag | 3.166842000 | -1.360579000 | 0.000301000 |
| Ag | -0.405121000 | 3.423031000 | 0.000105000 |
| C | -3.461438000 | -1.124139000 | -1.720432000 |
| C | -4.905807000 | -0.287601000 | -3.296313000 |
| H | -5.867658000 | -0.136241000 | -3.773151000 |
| C | -3.653428000 | 0.133167000 | -3.623097000 |
| H | -3.306639000 | 0.718268000 | -4.467544000 |
| Cl | -6.043019000 | -1.778947000 | -1.334873000 |
| C | -1.375043000 | -0.191554000 | -2.656857000 |
| C | -0.527060000 | -1.302180000 | -2.658773000 |
| H | -0.940744000 | -2.310769000 | -2.663574000 |
| C | 0.853410000 | -1.095190000 | -2.657021000 |
| C | 1.391165000 | 0.194538000 | -2.658409000 |
| H | 2.471486000 | 0.340514000 | -2.662851000 |
| C | 0.521727000 | 1.286632000 | -2.656768000 |
| C | -0.864069000 | 1.107475000 | -2.658088000 |
| H | -1.530760000 | 1.969989000 | -2.662214000 |
| C | 2.704636000 | -2.435270000 | -1.720722000 |
| C | 1.712012000 | -3.230363000 | -3.623459000 |
| H | 1.031890000 | -3.222741000 | -4.467897000 |
| C | 2.703020000 | -4.104171000 | -3.296855000 |
| H | 3.053242000 | -5.012626000 | -3.773815000 |
| Cl | 4.563241000 | -4.342908000 | -1.335406000 |
| C | 0.757269000 | 3.559864000 | -1.720373000 |
| C | 1.942269000 | 3.097394000 | -3.622914000 |
| H | 2.275661000 | 2.504520000 | -4.467337000 |
| C | 2.204157000 | 4.392339000 | -3.296077000 |
| H | 2.816272000 | 5.149590000 | -3.772861000 |
| Cl | 1.481174000 | 6.122911000 | -1.334688000 |
| C | -2.060654000 | -2.999981000 | 1.720180000 |
| C | -1.668704000 | -4.622848000 | 3.295660000 |
| H | -1.796791000 | -5.588170000 | 3.772304000 |
| C | -0.909496000 | -3.541664000 | 3.622646000 |
| H | -0.249928000 | -3.375575000 | 4.467088000 |
| Cl | -3.421980000 | -5.289038000 | 1.334343000 |
| C | -0.574125000 | -1.264501000 | 2.657102000 |
| C | 0.816512000 | -1.142915000 | 2.658754000 |
| H | 1.454566000 | -2.026852000 | 2.663281000 |
| C | 1.381380000 | 0.135160000 | 2.657146000 |
| C | 0.580742000 | 1.278671000 | 2.658885000 |
| H | 1.027124000 | 2.273249000 | 2.663845000 |
| C | -0.808554000 | 1.128770000 | 2.656852000 |
| C | -1.398590000 | -0.136335000 | 2.658792000 |
| H | -2.483121000 | -0.246952000 | 2.663706000 |
| C | 3.627926000 | -0.284622000 | 1.720801000 |
| C | 3.520953000 | 0.983044000 | 3.623288000 |
| H | 3.047120000 | 1.471209000 | 4.467606000 |
| C | 4.836991000 | 0.866058000 | 3.296714000 |
| H | 5.736913000 | 1.237716000 | 3.773630000 |
| Cl | 6.291075000 | -0.319174000 | 1.335712000 |
| C | -1.567862000 | 3.284203000 | 1.720255000 |
| C | -2.612898000 | 2.557783000 | 3.622406000 |
| H | -2.798929000 | 1.903387000 | 4.466710000 |
| C | -3.169394000 | 3.756045000 | 3.295681000 |
| H | -3.941286000 | 4.349658000 | 3.772386000 |
| Cl | -2.869344000 | 5.607852000 | 1.334722000 |
| N | -4.749637000 | -1.042490000 | -2.144037000 |
| N | -2.790531000 | -0.388377000 | -2.662036000 |
| N | 1.731900000 | -2.222359000 | -2.662333000 |
| N | 3.278490000 | -3.591413000 | -2.144511000 |
| N | 1.058973000 | 2.610925000 | -2.661973000 |
| N | 1.472236000 | 4.634594000 | -2.143872000 |
| N | -2.348237000 | -4.258396000 | 2.143539000 |
| N | -1.164592000 | -2.565911000 | 2.661881000 |
| N | 2.803700000 | 0.274358000 | 2.662258000 |
| N | 4.861441000 | 0.095387000 | 2.144568000 |
| N | -1.640259000 | 2.290903000 | 2.661685000 |
| N | -2.513822000 | 4.162484000 | 2.143721000 |

$[L_2(Br)_6 \rightarrow Ag_3]^{3+}$

| | | | |
|---|---|---|---|
| Ag | -0.654711000 | 3.385855000 | -0.000178000 |
| Ag | 3.259541000 | -1.126420000 | 0.000194000 |
| Ag | -2.605215000 | -2.259658000 | -0.000158000 |
| C | -1.818225000 | 3.158122000 | 1.710001000 |
| C | -3.459104000 | 3.491319000 | 3.274393000 |
| H | -4.279103000 | 4.014467000 | 3.753090000 |
| C | -2.811531000 | 2.339257000 | 3.600953000 |
| H | -2.949720000 | 1.668358000 | 4.441477000 |
| Br | -3.341426000 | 5.510588000 | 1.245956000 |
| C | -0.894535000 | 1.062756000 | 2.640702000 |
| C | 0.478826000 | 1.320485000 | 2.641969000 |
| H | 0.846094000 | 2.346804000 | 2.646616000 |
| C | 1.366600000 | 0.243166000 | 2.640539000 |
| C | 0.903175000 | -1.075072000 | 2.642146000 |
| H | 1.608312000 | -1.906340000 | 2.646821000 |
| C | -0.473703000 | -1.305175000 | 2.640750000 |
| C | -1.383632000 | -0.244747000 | 2.642188000 |
| H | -2.456080000 | -0.439875000 | 2.646793000 |
| C | 3.643393000 | -0.004881000 | 1.710655000 |
| C | 3.430174000 | 1.265359000 | 3.601137000 |
| H | 2.917892000 | 1.720717000 | 4.441311000 |
| C | 4.751813000 | 1.249929000 | 3.275174000 |
| H | 5.614722000 | 1.698545000 | 3.754104000 |
| Br | 6.442632000 | 0.137423000 | 1.248143000 |
| C | -1.826716000 | -3.152925000 | 1.710676000 |
| C | -0.620718000 | -3.603243000 | 3.601643000 |
| H | 0.029551000 | -3.387265000 | 4.441990000 |
| C | -1.294956000 | -4.740057000 | 3.275634000 |
| H | -1.338163000 | -5.711567000 | 3.754736000 |
| Br | -3.103179000 | -5.648241000 | 1.248083000 |
| C | 0.510839000 | 3.607608000 | -1.709875000 |
| C | 1.910307000 | 4.527900000 | -3.273636000 |
| H | 2.476404000 | 5.318876000 | -3.752306000 |
| C | 1.739106000 | 3.217362000 | -3.599840000 |
| H | 2.117866000 | 2.646075000 | -4.439987000 |
| Br | 1.046792000 | 6.358676000 | -1.246825000 |
| C | 0.434924000 | 1.318851000 | -2.640099000 |
| C | 1.375602000 | 0.287402000 | -2.641686000 |
| H | 2.443621000 | 0.505516000 | -2.646369000 |
| C | 0.925842000 | -1.035554000 | -2.640527000 |
| C | -0.437743000 | -1.334509000 | -2.642256000 |
| H | -0.782797000 | -2.368517000 | -2.647328000 |
| C | -1.358594000 | -0.283552000 | -2.640596000 |
| C | -0.935678000 | 1.046848000 | -2.641922000 |
| H | -1.658596000 | 1.862712000 | -2.646756000 |
| C | 2.869547000 | -2.246107000 | -1.710147000 |
| C | 1.918684000 | -3.113430000 | -3.601345000 |
| H | 1.234928000 | -3.155333000 | -4.441822000 |
| C | 2.968036000 | -3.916998000 | -3.275160000 |
| H | 3.370434000 | -4.802338000 | -3.754213000 |
| Br | 4.984430000 | -4.085421000 | -1.247525000 |
| C | -3.379042000 | -1.361943000 | -1.710787000 |
| C | -3.654440000 | -0.103930000 | -3.601428000 |
| H | -3.348744000 | 0.509486000 | -4.441637000 |
| C | -4.875108000 | -0.610938000 | -3.275593000 |
| H | -5.842964000 | -0.516449000 | -3.754717000 |
| Br | -6.029517000 | -2.273571000 | -1.248612000 |
| N | -2.833411000 | 3.957013000 | 2.128618000 |
| N | -1.814453000 | 2.155772000 | 2.646358000 |
| N | 2.773112000 | 0.493384000 | 2.646464000 |
| N | 4.842705000 | 0.474873000 | 2.129660000 |
| N | -0.960301000 | -2.648333000 | 2.646710000 |
| N | -2.011208000 | -4.431344000 | 2.129835000 |
| N | 1.155344000 | 4.727205000 | -2.128435000 |
| N | 0.881672000 | 2.675788000 | -2.645597000 |
| N | 1.877668000 | -2.100830000 | -2.646352000 |
| N | 3.517370000 | -3.363581000 | -2.129242000 |
| N | -2.757100000 | -0.575192000 | -2.646555000 |
| N | -4.670681000 | -1.363914000 | -2.130005000 |

$[L_2(SiH_3)_6 \rightarrow Ag_3]^{3+}$

| | | | |
|---|---|---|---|
| Ag | -3.447863000 | -0.287418000 | 0.001643000 |
| Ag | 1.974291000 | -2.841346000 | -0.000899000 |
| Ag | 1.474459000 | 3.129725000 | 0.000041000 |

| | | | |
|---|---|---|---|
| C | -3.554526000 | 0.916125000 | -1.696388000 |
| C | -4.271283000 | 2.406487000 | -3.245692000 |
| H | -4.971084000 | 3.067394000 | -3.746252000 |
| C | -2.983590000 | 2.100758000 | -3.556600000 |
| H | -2.353051000 | 2.419037000 | -4.378797000 |
| Si | -6.221231000 | 1.695620000 | -1.266300000 |
| H | -7.011242000 | 2.698570000 | -2.022197000 |
| H | -5.955281000 | 2.090030000 | 0.142121000 |
| H | -6.767246000 | 0.316945000 | -1.360210000 |
| C | -1.264170000 | 0.581487000 | -2.581672000 |
| C | -1.147822000 | -0.810961000 | -2.583482000 |
| H | -2.040154000 | -1.436780000 | -2.595704000 |
| C | 0.125848000 | -1.383372000 | -2.581683000 |
| C | 1.273564000 | -0.586365000 | -2.584632000 |
| H | 2.261713000 | -1.046205000 | -2.598015000 |
| C | 1.132428000 | 0.802885000 | -2.582676000 |
| C | -0.131639000 | 1.398330000 | -2.584467000 |
| H | -0.227486000 | 2.484021000 | -2.597447000 |
| C | 0.982955000 | -3.534282000 | -1.698038000 |
| C | -0.331851000 | -3.632059000 | -3.555822000 |
| H | -0.924134000 | -3.245075000 | -4.377000000 |
| C | 0.047971000 | -4.900097000 | -3.245833000 |
| H | -0.175160000 | -5.836535000 | -3.746206000 |
| Si | 1.640835000 | -6.233376000 | -1.268027000 |
| H | 1.159735000 | -6.206022000 | 0.138289000 |
| H | 1.174519000 | -7.418319000 | -2.029508000 |
| H | 3.107537000 | -6.011866000 | -1.354457000 |
| C | 2.568039000 | 2.619541000 | -1.699084000 |
| C | 3.307118000 | 1.532054000 | -3.559415000 |
| H | 3.266780000 | 0.826715000 | -4.381461000 |
| C | 4.215917000 | 2.494543000 | -3.249638000 |
| H | 5.137665000 | 2.770118000 | -3.751149000 |
| Si | 4.577694000 | 4.537830000 | -1.269628000 |
| H | 5.833951000 | 4.729826000 | -2.035366000 |
| H | 4.800491000 | 4.104330000 | 0.134780000 |
| H | 3.650826000 | 5.696388000 | -1.349642000 |
| C | -3.350858000 | -1.492780000 | 1.698929000 |
| C | -3.808324000 | -3.083261000 | 3.246981000 |
| H | -4.388231000 | -3.851522000 | 3.747534000 |
| C | -2.588969000 | -2.568202000 | 3.557073000 |
| H | -1.913678000 | -2.777840000 | 4.378607000 |
| Si | -5.849088000 | -2.707137000 | 1.267031000 |
| H | -6.472882000 | -3.810097000 | 2.038867000 |
| H | -5.519396000 | -3.077780000 | -0.134344000 |
| H | -6.606615000 | -1.430824000 | 1.337168000 |
| C | -1.146860000 | -0.783297000 | 2.582798000 |
| C | 0.105637000 | -1.400702000 | 2.584471000 |
| H | 0.191405000 | -2.487245000 | 2.596967000 |
| C | 1.253298000 | -0.603609000 | 2.582404000 |
| C | 1.161692000 | 0.789787000 | 2.585020000 |
| H | 2.059711000 | 1.407412000 | 2.597753000 |
| C | -0.102446000 | 1.385072000 | 2.583137000 |
| C | -1.263392000 | 0.609136000 | 2.585361000 |
| H | -2.247243000 | 1.078107000 | 2.598447000 |
| C | 2.970064000 | -2.156156000 | 1.696699000 |
| C | 3.520094000 | -0.961053000 | 3.556516000 |
| H | 3.364000000 | -0.272229000 | 4.378735000 |
| C | 4.576057000 | -1.758778000 | 3.245321000 |
| H | 5.531510000 | -1.877014000 | 3.745526000 |
| Si | 5.270275000 | -3.713330000 | 1.264395000 |
| H | 5.410871000 | -3.251652000 | -0.141667000 |
| H | 6.544092000 | -3.689373000 | 2.024797000 |
| H | 4.551929000 | -5.011144000 | 1.350474000 |
| C | 0.383966000 | 3.648453000 | 1.698587000 |
| C | -0.925804000 | 3.526506000 | 3.558530000 |
| H | -1.444345000 | 3.046528000 | 4.380493000 |
| C | -0.762801000 | 4.839998000 | 3.247955000 |
| H | -1.138013000 | 5.726373000 | 3.748585000 |
| Si | 0.583994000 | 6.419379000 | 1.268577000 |
| H | 0.120605000 | 6.308071000 | -0.139559000 |
| H | -0.079308000 | 7.509902000 | 2.025016000 |
| H | 2.066629000 | 6.449397000 | 1.361468000 |
| N | -4.607236000 | 1.678909000 | -2.107226000 |
| N | -2.560786000 | 1.178568000 | -2.600589000 |
| N | 0.257071000 | -2.804843000 | -2.600794000 |
| N | 0.848179000 | -4.827290000 | -2.108864000 |
| N | 2.297854000 | 1.627234000 | -2.602589000 |
| N | 3.754595000 | 3.149741000 | -2.111119000 |
| N | -4.261753000 | -2.420236000 | 2.109843000 |
| N | -2.326258000 | -1.587538000 | 2.601831000 |
| N | 2.539530000 | -1.222840000 | 2.600916000 |
| N | 4.228885000 | -2.481342000 | 2.107039000 |
| N | -0.209108000 | 2.808592000 | 2.602430000 |
| N | 0.036499000 | 4.901086000 | 2.109667000 |

$[L_2(Ph)_6 \rightarrow Ag_3]^{3+}$

| | | | |
|---|---|---|---|
| Ag | 3.363918000 | 0.807416000 | 0.033019000 |
| Ag | -2.405451000 | 2.461833000 | -0.046343000 |
| Ag | -0.979373000 | -3.287089000 | 0.035052000 |
| C | 3.666067000 | -0.390857000 | -1.640620000 |
| C | 4.651538000 | -1.729809000 | -3.193080000 |
| H | 5.465151000 | -2.274686000 | -3.658076000 |
| C | 3.338013000 | -1.628752000 | -3.530399000 |
| H | 2.783959000 | -2.039627000 | -4.366320000 |
| C | 1.379971000 | -0.410288000 | -2.583768000 |
| C | 1.053657000 | 0.948280000 | -2.595158000 |
| H | 1.841300000 | 1.701676000 | -2.591197000 |
| C | -0.291683000 | 1.321136000 | -2.608312000 |
| C | -1.304165000 | 0.357562000 | -2.618150000 |
| H | -2.350517000 | 0.661369000 | -2.637789000 |
| C | -0.953504000 | -0.992928000 | -2.606172000 |
| C | 0.385998000 | -1.390169000 | -2.591265000 |
| H | 0.645397000 | -2.448772000 | -2.585281000 |
| C | -1.496861000 | 3.290412000 | -1.726555000 |
| C | -0.144978000 | 3.631113000 | -3.533763000 |
| H | 0.519546000 | 3.356321000 | -4.344577000 |
| C | -0.720948000 | 4.821635000 | -3.217239000 |
| H | -0.653225000 | 5.791541000 | -3.696348000 |
| C | -2.113446000 | -2.970360000 | -1.679778000 |
| C | -2.953372000 | -2.091926000 | -3.612038000 |
| H | -3.001010000 | -1.418183000 | -4.459659000 |
| C | -3.709353000 | -3.175031000 | -3.287462000 |
| H | -4.570589000 | -3.613972000 | -3.778106000 |
| C | 3.046769000 | 2.022903000 | 1.693592000 |
| C | 3.216341000 | 3.752283000 | 3.159540000 |
| H | 3.678735000 | 4.615973000 | 3.623308000 |
| C | 2.097814000 | 3.055171000 | 3.494539000 |
| H | 1.395520000 | 3.187169000 | 4.309329000 |
| C | 0.984664000 | 1.010066000 | 2.603814000 |
| C | -0.352477000 | 1.411956000 | 2.598201000 |
| H | -0.617750000 | 2.469044000 | 2.586215000 |
| C | -1.351883000 | 0.435857000 | 2.603202000 |
| C | -1.033015000 | -0.922633000 | 2.618854000 |
| H | -1.816820000 | -1.679624000 | 2.629600000 |
| C | 0.312151000 | -1.301746000 | 2.623550000 |
| C | 1.328010000 | -0.344544000 | 2.620606000 |
| H | 2.375744000 | -0.644031000 | 2.630850000 |
| C | -3.286402000 | 1.647516000 | 1.653217000 |
| C | -3.647233000 | 0.473999000 | 3.577668000 |
| H | -3.387292000 | -0.146376000 | 4.427505000 |
| C | -4.816130000 | 1.081039000 | 3.238301000 |
| H | -5.789411000 | 1.066990000 | 3.715430000 |
| C | 0.183572000 | -3.603084000 | 1.732815000 |
| C | 1.502568000 | -3.279229000 | 3.567057000 |
| H | 1.943829000 | -2.728915000 | 4.389902000 |
| C | 1.555729000 | -4.600005000 | 3.246656000 |
| H | 2.058610000 | -5.427931000 | 3.733047000 |
| N | 4.828982000 | -0.975894000 | -2.039750000 |
| N | 2.752512000 | -0.803080000 | -2.574375000 |
| N | -0.638766000 | 2.703670000 | -2.620267000 |
| N | -1.541001000 | 4.593315000 | -2.119431000 |
| N | -1.980683000 | -1.984930000 | -2.621642000 |
| N | -3.186685000 | -3.690658000 | -2.108144000 |
| N | 3.780493000 | 3.107739000 | 2.065811000 |
| N | 2.015205000 | 1.994829000 | 2.596109000 |
| N | -2.723005000 | 0.835005000 | 2.601026000 |
| N | -4.577437000 | 1.783908000 | 2.064039000 |
| N | 0.650699000 | -2.686721000 | 2.638806000 |
| N | 0.745563000 | -4.777243000 | 2.132302000 |
| C | -0.932967000 | -7.683484000 | 0.521163000 |

| | | | |
|---|---|---|---|
| C | 0.167520000 | -8.484319000 | 0.206684000 |
| C | 1.457960000 | -8.060540000 | 0.535027000 |
| C | 1.654643000 | -6.832417000 | 1.167870000 |
| C | 0.545599000 | -6.041050000 | 1.475894000 |
| C | -0.751126000 | -6.459920000 | 1.166783000 |
| H | -1.943873000 | -8.015993000 | 0.283053000 |
| H | 0.020231000 | -9.446449000 | -0.285238000 |
| H | 2.318295000 | -8.686014000 | 0.294485000 |
| H | 2.661769000 | -6.486601000 | 1.406980000 |
| H | -1.609742000 | -5.851757000 | 1.454330000 |
| C | 6.187549000 | -1.181159000 | -0.008959000 |
| C | 7.418010000 | -1.052429000 | 0.637873000 |
| C | 8.538394000 | -0.602463000 | -0.066287000 |
| C | 8.429422000 | -0.275326000 | -1.420572000 |
| C | 7.201515000 | -0.388681000 | -2.074920000 |
| C | 6.090373000 | -0.840516000 | -1.360373000 |
| H | 5.313914000 | -1.569628000 | 0.516759000 |
| H | 7.508146000 | -1.328157000 | 1.689440000 |
| H | 9.501760000 | -0.519043000 | 0.438419000 |
| H | 9.303171000 | 0.072162000 | -1.973112000 |
| H | 7.104874000 | -0.117737000 | -3.127550000 |
| C | -3.816135000 | 7.592383000 | -0.218388000 |
| C | -2.471752000 | 7.821601000 | -0.522841000 |
| C | -1.713663000 | 6.830249000 | -1.147287000 |
| C | -2.319385000 | 5.613967000 | -1.471327000 |
| C | -3.666774000 | 5.377667000 | -1.186288000 |
| C | -4.409837000 | 6.371869000 | -0.548717000 |
| H | -4.404040000 | 8.372228000 | 0.266984000 |
| H | -2.007412000 | 8.775476000 | -0.270283000 |
| H | -0.657733000 | 6.995572000 | -1.368032000 |
| H | -4.137243000 | 4.440462000 | -1.486501000 |
| H | -5.462998000 | 6.192981000 | -0.329527000 |
| C | -4.786985000 | -7.065348000 | -0.147801000 |
| C | -4.401898000 | -7.151358000 | -1.488434000 |
| C | -3.863025000 | -6.039434000 | -2.138041000 |
| C | -3.723311000 | -4.842640000 | -1.432709000 |
| C | -4.113575000 | -4.739774000 | -0.095224000 |
| C | -4.640412000 | -5.861629000 | 0.547308000 |
| H | -5.214661000 | -7.934930000 | 0.352888000 |
| H | -4.518930000 | -8.088499000 | -2.033914000 |
| H | -3.543381000 | -6.104154000 | -3.179397000 |
| H | -4.031363000 | -3.782601000 | 0.422052000 |
| H | -4.961221000 | -5.788721000 | 1.587349000 |
| C | -7.538868000 | 4.027560000 | 0.066808000 |
| C | -6.838449000 | 3.032294000 | -0.620553000 |
| C | -5.858367000 | 2.284291000 | 0.034284000 |
| C | -5.579688000 | 2.554531000 | 1.376299000 |
| C | -6.270924000 | 3.546889000 | 2.074014000 |
| C | -7.256871000 | 4.280620000 | 1.411953000 |
| H | -8.315603000 | 4.598495000 | -0.443570000 |
| H | -7.073769000 | 2.820196000 | -1.664368000 |
| H | -5.332234000 | 1.476888000 | -0.477308000 |
| H | -6.032890000 | 3.751137000 | 3.119191000 |
| H | -7.804821000 | 5.054126000 | 1.951325000 |
| C | 5.988379000 | 2.628158000 | 1.117138000 |
| C | 4.976589000 | 3.549179000 | 1.400898000 |
| C | 5.108250000 | 4.897002000 | 1.058458000 |
| C | 6.271286000 | 5.324290000 | 0.416442000 |
| C | 7.283816000 | 4.410450000 | 0.112595000 |
| C | 7.139900000 | 3.065574000 | 0.461611000 |
| H | 5.891012000 | 1.588346000 | 1.431877000 |
| H | 4.304905000 | 5.602165000 | 1.278493000 |
| H | 6.383572000 | 6.375654000 | 0.149427000 |
| H | 8.191411000 | 4.751160000 | -0.386967000 |
| H | 7.933447000 | 2.350267000 | 0.242870000 |

$[L_2(C_2H_5)_6\rightarrow Au_3]^{3+}$

| | | | |
|---|---|---|---|
| Au | 1.305779000 | 3.231588000 | 0.144121000 |
| Au | -3.452837000 | -0.411299000 | -0.035759000 |
| Au | 2.076272000 | -2.738508000 | -0.032703000 |
| N | -0.135130000 | 4.979833000 | -1.914669000 |
| N | -0.290163000 | 2.898756000 | -2.462805000 |
| N | -2.262482000 | -1.560622000 | -2.624881000 |
| N | -4.157432000 | -2.487723000 | -2.170198000 |
| N | 2.588121000 | -1.039617000 | -2.541272000 |
| N | 4.316158000 | -2.237969000 | -2.057338000 |
| N | 3.502869000 | 3.322627000 | 2.273176000 |
| N | 2.157101000 | 1.680391000 | 2.658830000 |
| N | -2.669239000 | 0.982236000 | 2.586925000 |
| N | -4.717397000 | 1.418661000 | 2.066985000 |
| N | 0.352389000 | -2.849240000 | 2.509057000 |
| N | 1.029307000 | -4.820926000 | 1.951102000 |
| C | 0.246262000 | 3.739081000 | -1.519721000 |
| C | -0.888662000 | 4.927745000 | -3.073959000 |
| H | -1.275956000 | 5.818593000 | -3.556260000 |
| C | -0.998240000 | 3.616375000 | -3.421175000 |
| H | -1.480808000 | 3.140279000 | -4.266615000 |
| C | 0.255912000 | 6.231789000 | -1.240209000 |
| H | 0.305188000 | 6.012231000 | -0.165034000 |
| H | -0.560644000 | 6.949640000 | -1.396340000 |
| C | 1.581407000 | 6.774896000 | -1.758833000 |
| H | 2.396153000 | 6.058825000 | -1.579328000 |
| H | 1.822538000 | 7.712165000 | -1.239770000 |
| H | 1.535959000 | 6.988469000 | -2.835329000 |
| C | -0.136772000 | 1.479373000 | -2.476562000 |
| C | -1.271511000 | 0.668407000 | -2.522300000 |
| H | -2.269092000 | 1.106697000 | -2.532452000 |
| C | -1.110470000 | -0.719171000 | -2.561898000 |
| C | 0.160134000 | -1.296318000 | -2.563431000 |
| H | 0.279964000 | -2.378734000 | -2.602437000 |
| C | 1.281504000 | -0.463518000 | -2.518033000 |
| C | 1.146069000 | 0.924553000 | -2.476121000 |
| H | 2.023599000 | 1.570113000 | -2.448459000 |
| C | -3.291991000 | -1.546022000 | -1.717661000 |
| C | -2.491075000 | -2.506454000 | -3.618669000 |
| H | -1.807271000 | -2.661206000 | -4.445018000 |
| C | -3.691299000 | -3.080427000 | -3.330253000 |
| H | -4.248108000 | -3.849114000 | -3.855005000 |
| C | -5.455610000 | -2.800740000 | -1.543813000 |
| H | -5.314621000 | -2.714311000 | -0.457721000 |
| H | -5.674472000 | -3.853195000 | -1.768892000 |
| C | -6.569321000 | -1.883719000 | -2.032882000 |
| H | -6.349391000 | -0.835400000 | -1.785791000 |
| H | -7.514161000 | -2.163031000 | -1.547681000 |
| H | -6.711172000 | -1.964543000 | -3.119050000 |
| C | 3.050187000 | -1.972330000 | -1.647282000 |
| C | 3.562197000 | -0.731996000 | -3.485021000 |
| H | 3.388922000 | -0.033496000 | -4.295198000 |
| C | 4.645505000 | -1.497275000 | -3.178610000 |
| H | 5.610372000 | -1.579186000 | -3.667088000 |
| C | 5.208615000 | -3.228778000 | -1.426661000 |
| H | 5.045201000 | -3.162204000 | -0.342403000 |
| H | 6.238474000 | -2.905266000 | -1.628480000 |
| C | 4.958845000 | -4.641445000 | -1.938670000 |
| H | 3.932182000 | -4.963063000 | -1.712563000 |
| H | 5.654673000 | -5.336841000 | -1.450386000 |
| H | 5.116666000 | -4.711547000 | -3.023365000 |
| C | 2.371436000 | 2.735515000 | 1.808052000 |
| C | 3.999765000 | 2.650053000 | 3.375675000 |
| H | 4.891420000 | 2.974300000 | 3.901110000 |
| C | 3.156111000 | 1.611417000 | 3.624294000 |
| H | 3.149195000 | 0.873558000 | 4.417915000 |
| C | 4.164258000 | 4.484351000 | 1.651359000 |
| H | 4.591747000 | 5.084354000 | 2.466399000 |
| H | 3.373488000 | 5.081987000 | 1.178504000 |
| C | 5.229158000 | 4.075654000 | 0.642176000 |
| H | 6.018899000 | 3.472591000 | 1.110704000 |
| H | 5.699148000 | 4.973978000 | 0.220139000 |
| H | 4.785996000 | 3.497954000 | -0.181379000 |
| C | 1.037096000 | 0.797633000 | 2.597634000 |
| C | -0.256909000 | 1.326080000 | 2.600701000 |
| H | -0.410143000 | 2.404491000 | 2.634208000 |
| C | -1.343554000 | 0.451776000 | 2.564030000 |
| C | -1.154495000 | -0.932635000 | 2.529043000 |
| H | -2.012029000 | -1.604626000 | 2.504766000 |
| C | 0.146960000 | -1.436248000 | 2.527113000 |
| C | 1.251404000 | -0.580752000 | 2.565131000 |
| H | 2.262114000 | -0.987911000 | 2.573074000 |
| C | -3.632800000 | 0.720507000 | 1.646201000 |
| C | -3.155392000 | 1.837035000 | 3.570329000 |

| | | | |
|---|---|---|---|
| H | -2.557169000 | 2.151134000 | 4.417589000 |
| C | -4.449633000 | 2.100593000 | 3.240755000 |
| H | -5.194948000 | 2.706341000 | 3.744527000 |
| C | -6.030626000 | 1.408113000 | 1.395875000 |
| H | -6.500427000 | 2.380974000 | 1.593952000 |
| H | -5.835121000 | 1.346229000 | 0.316621000 |
| C | -6.912116000 | 0.258182000 | 1.866065000 |
| H | -6.445113000 | -0.711087000 | 1.640877000 |
| H | -7.880424000 | 0.303850000 | 1.349835000 |
| H | -7.103522000 | 0.311317000 | 2.946315000 |
| C | 1.108545000 | -3.518358000 | 1.579704000 |
| C | -0.189458000 | -3.732816000 | 3.436176000 |
| H | -0.800694000 | -3.402859000 | 4.267956000 |
| C | 0.248377000 | -4.972436000 | 3.083167000 |
| H | 0.074746000 | -5.938242000 | 3.545052000 |
| C | 1.727770000 | -5.928838000 | 1.272656000 |
| H | 1.712841000 | -5.704608000 | 0.197296000 |
| H | 1.124799000 | -6.832732000 | 1.432474000 |
| C | 3.151971000 | -6.113788000 | 1.780862000 |
| H | 3.754598000 | -5.213511000 | 1.594585000 |
| H | 3.620948000 | -6.958115000 | 1.257965000 |
| H | 3.171564000 | -6.331138000 | 2.857342000 |

$[L_2(CH_3)_6 \rightarrow Au_3]^{3+}$

| | | | |
|---|---|---|---|
| Au | -1.971314000 | -2.883150000 | -0.000606000 |
| Au | -1.511105000 | 3.148561000 | 0.000427000 |
| Au | 3.482957000 | -0.265831000 | 0.000589000 |
| C | -0.977230000 | -3.556133000 | 1.646942000 |
| C | -0.024009000 | -4.892287000 | 3.206295000 |
| H | 0.201916000 | -5.825028000 | 3.711676000 |
| C | 0.350301000 | -3.616912000 | 3.495352000 |
| H | 0.946076000 | -3.217065000 | 4.307521000 |
| C | -1.432317000 | -6.013553000 | 1.460370000 |
| H | -0.652015000 | -6.744131000 | 1.213985000 |
| H | -1.945501000 | -5.703504000 | 0.544291000 |
| H | -2.154382000 | -6.468001000 | 2.150737000 |
| C | -0.123899000 | -1.384503000 | 2.501743000 |
| C | -1.274563000 | -0.591100000 | 2.503747000 |
| H | -2.261195000 | -1.053667000 | 2.515099000 |
| C | -1.137411000 | 0.797718000 | 2.501949000 |
| C | 0.125062000 | 1.397449000 | 2.503374000 |
| H | 0.217888000 | 2.483177000 | 2.514442000 |
| C | 1.259286000 | 0.584302000 | 2.501772000 |
| C | 1.147423000 | -0.808889000 | 2.503374000 |
| H | 2.041178000 | -1.432264000 | 2.514532000 |
| C | -2.591255000 | 2.622880000 | 1.647373000 |
| C | -3.308258000 | 1.502649000 | 3.494978000 |
| H | -3.260168000 | 0.786357000 | 4.306805000 |
| C | -4.225602000 | 2.464530000 | 3.206030000 |
| H | -5.146563000 | 2.734904000 | 3.711187000 |
| C | -4.491955000 | 4.245684000 | 1.460965000 |
| H | -3.966988000 | 4.535138000 | 0.544822000 |
| H | -5.514903000 | 3.935423000 | 1.214729000 |
| H | -4.524219000 | 5.098138000 | 2.151466000 |
| C | 3.567232000 | 0.931426000 | 1.648267000 |
| C | 2.954549000 | 2.111544000 | 3.496151000 |
| H | 2.309782000 | 2.427418000 | 4.307904000 |
| C | 4.246286000 | 2.425442000 | 3.207889000 |
| H | 4.940568000 | 3.087734000 | 3.713655000 |
| C | 5.922725000 | 1.767055000 | 1.462980000 |
| H | 6.164470000 | 2.808143000 | 1.215951000 |
| H | 5.911796000 | 1.166954000 | 0.547319000 |
| H | 6.677321000 | 1.370138000 | 2.154023000 |
| C | -2.958291000 | -2.199767000 | -1.648227000 |
| C | -4.548695000 | -1.795697000 | -3.207976000 |
| H | -5.499631000 | -1.923460000 | -3.713795000 |
| C | -3.496005000 | -0.983906000 | -3.496226000 |
| H | -3.339332000 | -0.283238000 | -4.307991000 |
| C | -5.083074000 | -3.515723000 | -1.463000000 |
| H | -6.046954000 | -3.053759000 | -1.216332000 |
| H | -4.608019000 | -3.882130000 | -0.547157000 |
| H | -5.244605000 | -4.352995000 | -2.153935000 |
| C | -1.244060000 | -0.616127000 | -2.501658000 |
| C | -1.168955000 | 0.777415000 | -2.503246000 |
| H | -2.074028000 | 1.384252000 | -2.514734000 |
| C | 0.088695000 | 1.387181000 | -2.500998000 |
| C | 1.258006000 | 0.625408000 | -2.502488000 |
| H | 2.236003000 | 1.105966000 | -2.513426000 |
| C | 1.157242000 | -0.768632000 | -2.501126000 |
| C | -0.087147000 | -1.400407000 | -2.503099000 |
| H | -0.160017000 | -2.487652000 | -2.514573000 |
| C | -0.426077000 | 3.663125000 | -1.646835000 |
| C | 0.895568000 | 3.521829000 | -3.495054000 |
| H | 1.424026000 | 3.036181000 | -4.307043000 |
| C | 0.718365000 | 4.839243000 | -3.206475000 |
| H | 1.082752000 | 5.726924000 | -3.712145000 |
| C | -0.503967000 | 6.161166000 | -1.460823000 |
| H | -1.060282000 | 5.932485000 | -0.546002000 |
| H | 0.378043000 | 6.764197000 | -1.212338000 |
| H | -1.146967000 | 6.720654000 | -2.152213000 |
| C | 3.385764000 | -1.461588000 | -1.647540000 |
| C | 2.602087000 | -2.534358000 | -3.496221000 |
| H | 1.917091000 | -2.748722000 | -4.308181000 |
| C | 3.831572000 | -3.039951000 | -3.208147000 |
| H | 4.417932000 | -3.799167000 | -3.714340000 |
| C | 5.588059000 | -2.643289000 | -1.462743000 |
| H | 5.668086000 | -2.048199000 | -0.547236000 |
| H | 5.669626000 | -3.708910000 | -1.215508000 |
| H | 6.393936000 | -2.365149000 | -2.153902000 |
| N | -0.827762000 | -4.833953000 | 2.080506000 |
| N | -0.246786000 | -2.806572000 | 2.535960000 |
| N | -2.307506000 | 1.615213000 | 2.536109000 |
| N | -3.772804000 | 3.132051000 | 2.080777000 |
| N | 2.552207000 | 1.189050000 | 2.536603000 |
| N | 4.598606000 | 1.700137000 | 2.082447000 |
| N | -4.203075000 | -2.524018000 | -2.082407000 |
| N | -2.524582000 | -1.246677000 | -2.536591000 |
| N | 0.182712000 | 2.811441000 | -2.535490000 |
| N | -0.085019000 | 4.903512000 | -2.080742000 |
| N | 2.343621000 | -1.562282000 | -2.536077000 |
| N | 4.289261000 | -2.376954000 | -2.082189000 |

$[L_2(H)_6 \rightarrow Au_3]^{3+}$

| | | | |
|---|---|---|---|
| Au | 2.092951000 | 2.758165000 | -0.000462000 |
| Au | -3.435481000 | 0.433537000 | 0.001615000 |
| Au | 1.342584000 | -3.191344000 | -0.001180000 |
| C | 3.010169000 | 2.085189000 | -1.686054000 |
| C | 4.594157000 | 1.710796000 | -3.277671000 |
| H | 5.546582000 | 1.837324000 | -3.780408000 |
| C | 3.516197000 | 0.941460000 | -3.591628000 |
| H | 3.336290000 | 0.280290000 | -4.431167000 |
| H | 4.868436000 | 3.039348000 | -1.636718000 |
| C | 1.257161000 | 0.585295000 | -2.593946000 |
| C | 0.121656000 | 1.399415000 | -2.595720000 |
| H | 0.222240000 | 2.484682000 | -2.601822000 |
| C | -1.137741000 | 0.798403000 | -2.592662000 |
| C | -1.275084000 | -0.592019000 | -2.594766000 |
| H | -2.265275000 | -1.047495000 | -2.600106000 |
| C | -0.124896000 | -1.382185000 | -2.593096000 |
| C | 1.147951000 | -0.805888000 | -2.596174000 |
| H | 2.037516000 | -1.435649000 | -2.602618000 |
| C | -3.312527000 | 1.565994000 | -1.683040000 |
| C | -2.576235000 | 2.577411000 | -3.588376000 |
| H | -1.914254000 | 2.752696000 | -4.428255000 |
| C | -3.781323000 | 3.126009000 | -3.273310000 |
| H | -4.367465000 | 3.887856000 | -3.775199000 |
| C | 0.298575000 | -3.649881000 | -1.684926000 |
| C | -0.947570000 | -3.516966000 | -3.588811000 |
| H | -1.431234000 | -3.030909000 | -4.427948000 |
| C | -0.819966000 | -4.835050000 | -3.274462000 |
| H | -1.187321000 | -5.723323000 | -3.776345000 |
| H | 0.195687000 | -5.736221000 | -1.634525000 |
| C | 1.198502000 | 3.461080000 | 1.685099000 |
| C | 0.411409000 | 4.886348000 | 3.276185000 |
| H | 0.276698000 | 5.837870000 | 3.778506000 |
| C | -0.039010000 | 3.641114000 | 3.590763000 |
| H | -0.627190000 | 3.290129000 | 4.430523000 |
| H | 1.616703000 | 5.507689000 | 1.634786000 |

| | | | |
|---|---|---|---|
| C | 0.226209000 | 1.369271000 | 2.593899000 |
| C | -1.084281000 | 0.889792000 | 2.596295000 |
| H | -1.930149000 | 1.577116000 | 2.602359000 |
| C | -1.296817000 | -0.491159000 | 2.593691000 |
| C | -0.226294000 | -1.386304000 | 2.595359000 |
| H | -0.398552000 | -2.462519000 | 2.600852000 |
| C | 1.075927000 | -0.879932000 | 2.593053000 |
| C | 1.315873000 | 0.494757000 | 2.595655000 |
| H | 2.334028000 | 0.883710000 | 2.601106000 |
| C | -3.595109000 | -0.694627000 | 1.685999000 |
| C | -3.130783000 | -1.857972000 | 3.590305000 |
| H | -2.532082000 | -2.192451000 | 4.429364000 |
| C | -4.434517000 | -2.090608000 | 3.276321000 |
| H | -5.190729000 | -2.683711000 | 3.778553000 |
| H | -5.576457000 | -1.356303000 | 1.636275000 |
| C | 2.400679000 | -2.767633000 | 1.682764000 |
| C | 3.176987000 | -1.786263000 | 3.587919000 |
| H | 3.167799000 | -1.101556000 | 4.427821000 |
| C | 4.029990000 | -2.798809000 | 3.272361000 |
| H | 4.921876000 | -3.157925000 | 3.773801000 |
| H | 3.964041000 | -4.152972000 | 1.630740000 |
| N | 4.256957000 | 2.388257000 | -2.122206000 |
| N | 2.553979000 | 1.186186000 | -2.613051000 |
| N | -2.306484000 | 1.621055000 | -2.610648000 |
| N | -4.198697000 | 2.494452000 | -2.118018000 |
| N | -0.253045000 | -2.805678000 | -2.611537000 |
| N | -0.063045000 | -4.881320000 | -2.120039000 |
| N | 1.154547000 | 4.743607000 | 2.120700000 |
| N | 0.455820000 | 2.779959000 | 2.612550000 |
| N | -2.633269000 | -0.997805000 | 2.612581000 |
| N | -4.683358000 | -1.374712000 | 2.121618000 |
| N | 2.182880000 | -1.784109000 | 2.610621000 |
| N | 3.533827000 | -3.370791000 | 2.117185000 |
| H | -5.068014000 | 2.698083000 | -1.631862000 |

$[L_2(F)_6 \rightarrow Au_3]^{3+}$

| | | | |
|---|---|---|---|
| Au | 3.429750000 | -0.154868000 | 0.000242000 |
| Au | -1.581039000 | 3.046833000 | 0.000058000 |
| Au | -1.849145000 | -2.892152000 | -0.000222000 |
| C | 3.402359000 | -1.266139000 | -1.697162000 |
| C | 4.064252000 | -2.785956000 | -3.295402000 |
| H | 4.749194000 | -3.485205000 | -3.762717000 |
| C | 2.821585000 | -2.334299000 | -3.635864000 |
| H | 2.206867000 | -2.564461000 | -4.498814000 |
| F | 5.510094000 | -2.299173000 | -1.458207000 |
| C | 1.200566000 | -0.695194000 | -2.668310000 |
| C | 1.217987000 | 0.702098000 | -2.670037000 |
| H | 2.164855000 | 1.242354000 | -2.666143000 |
| C | 0.002950000 | 1.387373000 | -2.668218000 |
| C | -1.215821000 | 0.703735000 | -2.670217000 |
| H | -2.157127000 | 1.253641000 | -2.666427000 |
| C | -1.201804000 | -0.691164000 | -2.668439000 |
| C | -0.000402000 | -1.404858000 | -2.670031000 |
| H | -0.005886000 | -2.495006000 | -2.665917000 |
| C | -0.604051000 | 3.579294000 | -1.696736000 |
| C | 0.612365000 | 3.611105000 | -3.634810000 |
| H | 1.119460000 | 3.194115000 | -4.497658000 |
| C | 0.382070000 | 4.912993000 | -3.293955000 |
| H | 0.645463000 | 5.855966000 | -3.760759000 |
| F | -0.763276000 | 5.921067000 | -1.456904000 |
| C | -2.797705000 | -2.312199000 | -1.697582000 |
| C | -3.431914000 | -1.274696000 | -3.636171000 |
| H | -3.323613000 | -0.627173000 | -4.499026000 |
| C | -4.444787000 | -2.124601000 | -3.295763000 |
| H | -5.392953000 | -2.367656000 | -3.763079000 |
| F | -4.746795000 | -3.620152000 | -1.458672000 |
| C | 3.502274000 | 0.954608000 | 1.697463000 |
| C | 4.297965000 | 2.408970000 | 3.295586000 |
| H | 5.042958000 | 3.043846000 | 3.762914000 |
| C | 3.019648000 | 2.071016000 | 3.635896000 |
| H | 2.427989000 | 2.355711000 | 4.498697000 |
| F | 5.694421000 | 1.793838000 | 1.458675000 |
| C | 1.257805000 | 0.584348000 | 2.668339000 |
| C | 0.125628000 | 1.399296000 | 2.669868000 |
| H | 0.218362000 | 2.485508000 | 2.665651000 |
| C | -1.135187000 | 0.796719000 | 2.668271000 |
| C | -1.274788000 | -0.591240000 | 2.670107000 |
| H | -2.261806000 | -1.054112000 | 2.666232000 |
| C | -0.122555000 | -1.381866000 | 2.668273000 |
| C | 1.149284000 | -0.808839000 | 2.670271000 |
| H | 2.043629000 | -1.432203000 | 2.666533000 |
| C | -2.578489000 | 2.554783000 | 1.697103000 |
| C | -3.303897000 | 1.578667000 | 3.635588000 |
| H | -3.254496000 | 0.924043000 | 4.498468000 |
| C | -4.236073000 | 2.516299000 | 3.294960000 |
| H | -5.158604000 | 2.843734000 | 3.762079000 |
| F | -4.401906000 | 4.032828000 | 1.457711000 |
| C | -0.924404000 | -3.510309000 | 1.696840000 |
| C | 0.283875000 | -3.651324000 | 3.635149000 |
| H | 0.826341000 | -3.281602000 | 4.498033000 |
| C | -0.062736000 | -4.927211000 | 3.294416000 |
| H | 0.114535000 | -5.890014000 | 3.761389000 |
| F | -1.294008000 | -5.828237000 | 1.457332000 |
| N | 4.354964000 | -2.111365000 | -2.134345000 |
| N | 2.438273000 | -1.412898000 | -2.664905000 |
| N | 0.005471000 | 2.818098000 | -2.664518000 |
| N | -0.348100000 | 4.827043000 | -2.133303000 |
| N | -2.442278000 | -1.404082000 | -2.665316000 |
| N | -4.006177000 | -2.713994000 | -2.134782000 |
| N | 4.526987000 | 1.710794000 | 2.134661000 |
| N | 2.555138000 | 1.187683000 | 2.665013000 |
| N | -2.306434000 | 1.618431000 | 2.664946000 |
| N | -3.745986000 | 3.063736000 | 2.134018000 |
| N | -0.248946000 | -2.806993000 | 2.664663000 |
| N | -0.782008000 | -4.775978000 | 2.133628000 |

$[L_2(Cl)_6 \rightarrow Au_3]^{3+}$

| | | | |
|---|---|---|---|
| Au | -3.418002000 | 0.395416000 | -0.000534000 |
| Au | 1.366605000 | -3.157812000 | 0.000067000 |
| Au | 2.051538000 | 2.762621000 | 0.000105000 |
| C | -3.303303000 | 1.542609000 | -1.673035000 |
| C | -3.791821000 | 3.123733000 | -3.257503000 |
| H | -4.392738000 | 3.886029000 | -3.740354000 |
| C | -2.587828000 | 2.577035000 | -3.579541000 |
| H | -1.934713000 | 2.763296000 | -4.424604000 |
| Cl | -5.645789000 | 2.825314000 | -1.300851000 |
| C | -1.137168000 | 0.794716000 | -2.605765000 |
| C | -1.272069000 | -0.596139000 | -2.607517000 |
| H | -2.261165000 | -1.054066000 | -2.608170000 |
| C | -0.119540000 | -1.382323000 | -2.605375000 |
| C | 1.152448000 | -0.803736000 | -2.607000000 |
| H | 2.043522000 | -1.431431000 | -2.607363000 |
| C | 1.257063000 | 0.587486000 | -2.605258000 |
| C | 0.119971000 | 1.399731000 | -2.607476000 |
| H | 0.217994000 | 2.485283000 | -2.608209000 |
| C | 0.315837000 | -3.632166000 | -1.672438000 |
| C | -0.937775000 | -3.529872000 | -3.578917000 |
| H | -1.425664000 | -3.057456000 | -4.424005000 |
| C | -0.809274000 | -4.845890000 | -3.256780000 |
| H | -1.169039000 | -5.747475000 | -3.739540000 |
| Cl | 0.376319000 | -6.302131000 | -1.300095000 |
| C | 2.987776000 | 2.089569000 | -1.672260000 |
| C | 3.526213000 | 0.952276000 | -3.578393000 |
| H | 3.361140000 | 0.293395000 | -4.423387000 |
| C | 4.601709000 | 1.721480000 | -3.256207000 |
| H | 5.562494000 | 1.860483000 | -3.738820000 |
| Cl | 5.269926000 | 3.476668000 | -1.299796000 |
| C | -3.568706000 | -0.747296000 | 1.672181000 |
| C | -4.405672000 | -2.174766000 | 3.256772000 |
| H | -5.164886000 | -2.779575000 | 3.739620000 |
| C | -3.108699000 | -1.917406000 | 3.578979000 |
| H | -2.515535000 | -2.247715000 | 4.424221000 |
| Cl | -6.142170000 | -1.461129000 | 1.299696000 |
| C | -1.289096000 | -0.513874000 | 2.605162000 |
| C | -0.203510000 | -1.390180000 | 2.607036000 |
| H | -0.356154000 | -2.469397000 | 2.607545000 |
| C | 1.089120000 | -0.859287000 | 2.605343000 |
| C | 1.305218000 | 0.519012000 | 2.607074000 |

| | | | |
|---|---|---|---|
| H | 2.316149000 | 0.926448000 | 2.607469000 |
| C | 0.199133000 | 1.372972000 | 2.605247000 |
| C | -1.102567000 | 0.870991000 | 2.606973000 |
| H | -1.960823000 | 1.542851000 | 2.607333000 |
| C | 2.431338000 | -2.716900000 | 1.672841000 |
| C | 3.214425000 | -1.733031000 | 3.579513000 |
| H | 3.203822000 | -1.054027000 | 4.424633000 |
| C | 4.085914000 | -2.727538000 | 3.257561000 |
| H | 4.989306000 | -3.082429000 | 3.740549000 |
| Cl | 4.336497000 | -4.588557000 | 1.300876000 |
| C | 1.136933000 | 3.464143000 | 1.672801000 |
| C | -0.106877000 | 3.650392000 | 3.579343000 |
| H | -0.689698000 | 3.301690000 | 4.424394000 |
| C | 0.318772000 | 4.902360000 | 3.257491000 |
| H | 0.174447000 | 5.862162000 | 3.740495000 |
| Cl | 1.805596000 | 6.049805000 | 1.301052000 |
| N | -4.195834000 | 2.475824000 | -2.101364000 |
| N | -2.308358000 | 1.614757000 | -2.613464000 |
| N | -0.244110000 | -2.806644000 | -2.612909000 |
| N | -0.046125000 | -4.871754000 | -2.100657000 |
| N | 2.552868000 | 1.191733000 | -2.612650000 |
| N | 4.242388000 | 2.395616000 | -2.100316000 |
| N | -4.650849000 | -1.451929000 | 2.100456000 |
| N | -2.616679000 | -1.044592000 | 2.612799000 |
| N | 2.212581000 | -1.743630000 | 2.613283000 |
| N | 3.582658000 | -3.301554000 | 2.101314000 |
| N | 0.403275000 | 2.788075000 | 2.613135000 |
| N | 1.067641000 | 4.753530000 | 2.101323000 |

$[L_2(Br)_6 \rightarrow Au_3]^{3+}$

| | | | |
|---|---|---|---|
| Au | -3.383958000 | 0.641392000 | 0.000585000 |
| Au | 1.137380000 | -3.250230000 | -0.000204000 |
| Au | 2.246795000 | 2.609294000 | 0.000032000 |
| C | -3.183625000 | 1.786901000 | -1.665365000 |
| C | -3.542386000 | 3.405247000 | -3.240398000 |
| H | -4.076785000 | 4.214057000 | -3.725583000 |
| C | -2.382454000 | 2.769936000 | -3.563279000 |
| H | -1.714446000 | 2.909499000 | -4.405741000 |
| Br | -5.560875000 | 3.284463000 | -1.212697000 |
| C | -1.075677000 | 0.878235000 | -2.593385000 |
| C | -1.314818000 | -0.498533000 | -2.594722000 |
| H | -2.335597000 | -0.880466000 | -2.594986000 |
| C | -0.224945000 | -1.369593000 | -2.593126000 |
| C | 1.086926000 | -0.888265000 | -2.595132000 |
| H | 1.928056000 | -1.581346000 | -2.595956000 |
| C | 1.296406000 | 0.491132000 | -2.593578000 |
| C | 0.223620000 | 1.386542000 | -2.595075000 |
| H | 0.403176000 | 2.461547000 | -2.595650000 |
| C | 0.044023000 | -3.649430000 | -1.665435000 |
| C | -1.210416000 | -3.447501000 | -3.561729000 |
| H | -1.666505000 | -2.938893000 | -4.403604000 |
| C | -1.179774000 | -4.769694000 | -3.238935000 |
| H | -1.613364000 | -5.637044000 | -3.723569000 |
| Br | -0.063148000 | -6.456902000 | -1.212362000 |
| C | 3.137557000 | 1.862959000 | -1.666590000 |
| C | 3.587638000 | 0.677008000 | -3.564303000 |
| H | 3.374217000 | 0.028329000 | -4.406395000 |
| C | 4.717832000 | 1.364184000 | -3.242200000 |
| H | 5.685246000 | 1.422546000 | -3.727859000 |
| Br | 5.623049000 | 3.173647000 | -1.215721000 |
| C | -3.615729000 | -0.499167000 | 1.665792000 |
| C | -4.540961000 | -1.876292000 | 3.239357000 |
| H | -5.333881000 | -2.434530000 | 3.723927000 |
| C | -3.229048000 | -1.709246000 | 3.562252000 |
| H | -2.658099000 | -2.084467000 | 4.404087000 |
| Br | -6.375818000 | -1.024057000 | 1.212726000 |
| C | -1.321283000 | -0.425099000 | 2.593703000 |
| C | -0.297772000 | -1.373257000 | 2.595040000 |
| H | -0.523641000 | -2.439492000 | 2.595186000 |
| C | 1.028151000 | -0.932059000 | 2.593745000 |
| C | 1.337461000 | 0.428394000 | 2.595487000 |
| H | 2.373753000 | 0.765968000 | 2.596167000 |
| C | 0.292404000 | 1.356036000 | 2.593818000 |
| C | -1.040495000 | 0.943792000 | 2.595527000 |
| H | -1.850933000 | 1.672511000 | 2.596128000 |
| C | 2.240488000 | -2.881286000 | 1.665518000 |
| C | 3.093543000 | -1.942526000 | 3.563225000 |
| H | 3.132250000 | -1.261066000 | 4.405582000 |
| C | 3.894665000 | -2.994721000 | 3.240121000 |
| H | 4.774333000 | -3.402459000 | 3.724992000 |
| Br | 4.076041000 | -5.008274000 | 1.212063000 |
| C | 1.376269000 | 3.379842000 | 1.666334000 |
| C | 0.135746000 | 3.650029000 | 3.563316000 |
| H | -0.474628000 | 3.343188000 | 4.405188000 |
| C | 0.648090000 | 4.869426000 | 3.240998000 |
| H | 0.562218000 | 5.835022000 | 3.726191000 |
| Br | 2.304725000 | 6.031857000 | 1.214992000 |
| N | -4.001187000 | 2.787140000 | -2.088802000 |
| N | -2.181136000 | 1.784199000 | -2.601929000 |
| N | -0.456434000 | -2.780020000 | -2.601314000 |
| N | -0.413561000 | -4.857722000 | -2.088332000 |
| N | 2.633692000 | 0.995663000 | -2.602616000 |
| N | 4.412342000 | 2.070937000 | -2.090727000 |
| N | -4.742259000 | -1.131868000 | 2.088692000 |
| N | -2.681473000 | -0.864173000 | 2.601826000 |
| N | 2.088567000 | -1.890397000 | 2.602132000 |
| N | 3.351583000 | -3.540531000 | 2.088651000 |
| N | 0.592672000 | 2.753437000 | 2.602201000 |
| N | 1.392880000 | 4.671540000 | 2.089983000 |

$[L_2(SiH_3)_6 \rightarrow Au_3]^{3+}$

| | | | |
|---|---|---|---|
| Au | -2.792980000 | 2.063076000 | -0.000076000 |
| Au | -0.390449000 | -3.450111000 | -0.000261000 |
| Au | 3.183346000 | 1.386854000 | -0.000191000 |
| C | -2.100882000 | 3.014010000 | -1.663642000 |
| C | -1.688185000 | 4.600705000 | -3.219697000 |
| H | -1.793379000 | 5.554977000 | -3.724856000 |
| C | -0.909514000 | 3.529794000 | -3.529101000 |
| H | -0.227805000 | 3.357404000 | -4.353900000 |
| Si | -3.616729000 | 5.353592000 | -1.234620000 |
| H | -3.580182000 | 6.604066000 | -2.032626000 |
| H | -3.130541000 | 5.526179000 | 0.158602000 |
| H | -4.926011000 | 4.654656000 | -1.290012000 |
| C | -0.581849000 | 1.261052000 | -2.546780000 |
| C | -1.398151000 | 0.126899000 | -2.549456000 |
| H | -2.483026000 | 0.230205000 | -2.557589000 |
| C | -0.800534000 | -1.134259000 | -2.547215000 |
| C | 0.589799000 | -1.274099000 | -2.549314000 |
| H | 1.042855000 | -2.265253000 | -2.557193000 |
| C | 1.383151000 | -0.125947000 | -2.547018000 |
| C | 0.809150000 | 1.148060000 | -2.549021000 |
| H | 1.441037000 | 2.035947000 | -2.556559000 |
| C | -1.560048000 | -3.325961000 | -1.663818000 |
| C | -2.601937000 | -2.551824000 | -3.529439000 |
| H | -2.793146000 | -1.875256000 | -4.354323000 |
| C | -3.140780000 | -3.761231000 | -3.219802000 |
| H | -3.914926000 | -4.329107000 | -3.724876000 |
| Si | -2.830364000 | -5.807552000 | -1.234322000 |
| H | -3.226891000 | -5.472025000 | 0.157581000 |
| H | -3.929204000 | -6.402107000 | -2.034877000 |
| H | -1.570071000 | -6.591648000 | -1.285761000 |
| C | 3.660853000 | 0.312247000 | -1.663873000 |
| C | 3.511592000 | -0.976441000 | -3.529972000 |
| H | 3.021301000 | -1.480241000 | -4.354920000 |
| C | 4.828391000 | -0.837938000 | -3.220529000 |
| H | 5.707337000 | -1.223899000 | -3.725881000 |
| Si | 6.444963000 | 0.454238000 | -1.234668000 |
| H | 7.509492000 | -0.202449000 | -2.033023000 |
| H | 6.351197000 | -0.053891000 | 0.158251000 |
| H | 6.494759000 | 1.937598000 | -1.289136000 |
| C | -3.498461000 | 1.122851000 | 1.663852000 |
| C | -4.893405000 | 0.262900000 | 3.220768000 |
| H | -5.836264000 | 0.083531000 | 3.726471000 |
| C | -3.640899000 | -0.166689000 | 3.529934000 |
| H | -3.275580000 | -0.767199000 | 4.354963000 |
| Si | -6.180684000 | 1.883001000 | 1.234798000 |
| H | -7.362571000 | 1.494577000 | 2.043549000 |
| H | -6.213888000 | 1.354007000 | -0.153121000 |

| | | | |
|---|---|---|---|
| H | -5.889341000 | 3.338781000 | 1.274308000 |
| C | -1.376235000 | 0.186276000 | 2.547090000 |
| C | -0.859808000 | -1.110243000 | 2.549412000 |
| H | -1.522981000 | -1.975011000 | 2.557070000 |
| C | 0.526578000 | -1.284857000 | 2.546871000 |
| C | 1.391197000 | -0.189366000 | 2.549006000 |
| H | 2.471669000 | -0.331505000 | 2.556460000 |
| C | 0.849335000 | 1.098616000 | 2.546891000 |
| C | -0.531718000 | 1.299610000 | 2.549073000 |
| H | -0.949013000 | 2.306341000 | 2.556887000 |
| C | 0.776929000 | -3.590928000 | 1.663496000 |
| C | 1.964834000 | -3.069500000 | 3.529594000 |
| H | 2.302193000 | -2.452858000 | 4.354626000 |
| C | 2.219492000 | -4.368844000 | 3.220124000 |
| H | 2.846710000 | -5.095544000 | 3.725483000 |
| Si | 1.459389000 | -6.293880000 | 1.234472000 |
| H | 1.919721000 | -6.055387000 | -0.157796000 |
| H | 2.398274000 | -7.118925000 | 2.034241000 |
| H | 0.055755000 | -6.776392000 | 1.287089000 |
| C | 2.721377000 | 2.468151000 | 1.663466000 |
| C | 1.676789000 | 3.235875000 | 3.530207000 |
| H | 0.974225000 | 3.219753000 | 4.355359000 |
| C | 2.675094000 | 4.105704000 | 3.220879000 |
| H | 2.991375000 | 5.011889000 | 3.726528000 |
| Si | 4.722724000 | 4.408984000 | 1.235427000 |
| H | 4.287850000 | 4.685925000 | -0.157913000 |
| H | 4.966524000 | 5.636042000 | 2.033381000 |
| H | 5.842573000 | 3.434970000 | 1.291187000 |
| N | -2.411389000 | 4.275432000 | -2.076411000 |
| N | -1.179458000 | 2.558206000 | -2.567865000 |
| N | -1.625356000 | -2.300239000 | -2.568277000 |
| N | -2.497675000 | -4.225153000 | -2.076512000 |
| N | 2.805308000 | -0.257209000 | -2.568358000 |
| N | 4.908474000 | -0.049582000 | -2.076852000 |
| N | -4.795240000 | 1.049564000 | 2.077354000 |
| N | -2.791767000 | 0.376452000 | 2.568262000 |
| N | 1.069680000 | -2.605795000 | 2.568084000 |
| N | 1.489403000 | -4.677101000 | 2.076505000 |
| N | 1.721912000 | 2.229308000 | 2.568115000 |
| N | 3.306600000 | 3.627693000 | 2.076926000 |

$[L_2(Ph)_6 \rightarrow Au_3]^{3+}$

| | | | |
|---|---|---|---|
| Au | 3.374093000 | -0.808230000 | -0.030157000 |
| Au | -2.411878000 | -2.467562000 | 0.041618000 |
| Au | -0.981985000 | 3.293006000 | -0.031396000 |
| C | 3.677372000 | 0.362824000 | 1.603512000 |
| C | 4.656602000 | 1.679236000 | 3.172309000 |
| H | 5.469696000 | 2.214890000 | 3.648571000 |
| C | 3.339510000 | 1.583788000 | 3.496994000 |
| H | 2.780153000 | 1.991653000 | 4.330679000 |
| C | 1.378130000 | 0.389057000 | 2.529504000 |
| C | 1.039818000 | -0.966710000 | 2.538048000 |
| H | 1.820125000 | -1.727389000 | 2.530372000 |
| C | -0.308418000 | -1.325904000 | 2.548397000 |
| C | -1.312464000 | -0.353349000 | 2.558298000 |
| H | -2.361598000 | -0.646954000 | 2.572636000 |
| C | -0.948404000 | 0.992729000 | 2.548538000 |
| C | 0.394668000 | 1.378243000 | 2.536600000 |
| H | 0.663965000 | 2.434169000 | 2.529196000 |
| C | -1.527789000 | -3.297762000 | 1.675326000 |
| C | -0.180273000 | -3.633032000 | 3.481085000 |
| H | 0.486000000 | -3.356913000 | 4.289825000 |
| C | -0.763564000 | -4.821921000 | 3.172188000 |
| H | -0.701611000 | -5.789618000 | 3.656324000 |
| C | -2.102847000 | 2.986661000 | 1.636256000 |
| C | -2.934155000 | 2.105401000 | 3.565500000 |
| H | -2.981472000 | 1.427267000 | 4.409456000 |
| C | -3.684448000 | 3.195379000 | 3.250990000 |
| H | -4.539513000 | 3.638289000 | 3.748607000 |
| C | 3.075405000 | -2.005552000 | -1.648030000 |
| C | 3.248352000 | -3.718662000 | -3.125468000 |
| H | 3.713407000 | -4.576417000 | -3.597311000 |
| C | 2.124658000 | -3.024897000 | -3.449650000 |
| H | 1.419231000 | -3.153745000 | -4.262046000 |
| C | 0.999226000 | -0.994243000 | -2.544616000 |
| C | -0.333072000 | -1.409260000 | -2.541023000 |
| H | -0.588271000 | -2.468595000 | -2.527076000 |
| C | -1.341120000 | -0.442013000 | -2.545878000 |
| C | -1.036146000 | 0.918858000 | -2.559104000 |
| H | -1.827105000 | 1.668116000 | -2.566604000 |
| C | 0.305688000 | 1.309907000 | -2.561290000 |
| C | 1.330581000 | 0.363479000 | -2.558883000 |
| H | 2.375177000 | 0.673094000 | -2.564181000 |
| C | -3.281305000 | -1.669521000 | -1.613341000 |
| C | -3.628213000 | -0.498937000 | -3.536666000 |
| H | -3.363652000 | 0.124262000 | -4.382831000 |
| C | -4.796579000 | -1.113491000 | -3.209342000 |
| H | -5.765333000 | -1.105404000 | -3.695528000 |
| C | 0.163953000 | 3.620536000 | -1.680303000 |
| C | 1.480091000 | 3.293055000 | -3.510347000 |
| H | 1.923807000 | 2.740352000 | -4.330077000 |
| C | 1.525502000 | 4.615914000 | -3.197780000 |
| H | 2.023244000 | 5.444354000 | -3.688364000 |
| N | 4.843217000 | 0.934575000 | 2.015467000 |
| N | 2.754507000 | 0.771348000 | 2.530758000 |
| N | -0.666053000 | -2.706471000 | 2.563839000 |
| N | -1.583114000 | -4.599135000 | 2.073975000 |
| N | -1.968025000 | 1.993380000 | 2.570578000 |
| N | -3.167741000 | 3.715922000 | 2.072230000 |
| N | 3.816592000 | -3.082712000 | -2.029943000 |
| N | 2.037427000 | -1.972061000 | -2.544293000 |
| N | -2.709732000 | -0.852224000 | -2.553147000 |
| N | -4.568506000 | -1.817034000 | -2.034403000 |
| N | 0.633883000 | 2.698057000 | -2.579938000 |
| N | 0.716262000 | 4.798325000 | -2.084651000 |
| C | -0.960745000 | 7.740838000 | -0.537960000 |
| C | 0.143336000 | 8.531484000 | -0.210436000 |
| C | 1.434929000 | 8.087082000 | -0.504961000 |
| C | 1.628178000 | 6.848851000 | -1.118613000 |
| C | 0.514919000 | 6.068787000 | -1.439811000 |
| C | -0.782482000 | 6.506450000 | -1.163290000 |
| H | -1.971629000 | 8.089246000 | -0.324319000 |
| H | -0.001908000 | 9.501726000 | 0.265960000 |
| H | 2.298183000 | 8.704147000 | -0.253503000 |
| H | 2.635079000 | 6.486658000 | -1.333234000 |
| H | -1.642332000 | 5.902473000 | -1.454749000 |
| C | 6.236471000 | 1.176777000 | 0.010954000 |
| C | 7.478171000 | 1.057872000 | -0.615452000 |
| C | 8.582723000 | 0.582598000 | 0.097281000 |
| C | 8.446756000 | 0.218705000 | 1.439587000 |
| C | 7.207440000 | 0.322214000 | 2.073685000 |
| C | 6.113415000 | 0.800849000 | 1.350555000 |
| H | 5.373700000 | 1.578973000 | -0.521605000 |
| H | 7.589357000 | 1.360041000 | -1.657660000 |
| H | 9.554944000 | 0.507360000 | -0.391535000 |
| H | 9.307866000 | -0.149356000 | 1.998579000 |
| H | 7.089193000 | 0.024255000 | 3.116754000 |
| C | -3.864031000 | -7.622615000 | 0.221078000 |
| C | -2.510445000 | -7.835397000 | 0.494876000 |
| C | -1.751193000 | -6.834023000 | 1.101428000 |
| C | -2.365111000 | -5.624980000 | 1.436669000 |
| C | -3.720498000 | -5.403761000 | 1.181155000 |
| C | -4.464902000 | -6.408683000 | 0.562647000 |
| H | -4.453411000 | -8.410210000 | -0.249817000 |
| H | -2.040266000 | -8.783817000 | 0.232853000 |
| H | -0.688860000 | -6.985809000 | 1.300141000 |
| H | -4.193327000 | -4.468985000 | 1.483992000 |
| H | -5.524397000 | -6.242814000 | 0.365460000 |
| C | -4.787904000 | 7.109836000 | 0.163802000 |
| C | -4.362596000 | 7.189475000 | 1.492505000 |
| C | -3.813974000 | 6.071311000 | 2.122896000 |
| C | -3.705479000 | 4.875992000 | 1.409953000 |
| C | -4.134153000 | 4.779254000 | 0.084146000 |
| C | -4.670595000 | 5.907218000 | -0.538975000 |
| H | -5.223607000 | 7.984001000 | -0.321813000 |
| H | -4.455996000 | 8.125907000 | 2.043703000 |
| H | -3.464193000 | 6.129688000 | 3.154886000 |
| H | -4.069035000 | 3.824671000 | -0.439551000 |
| H | -5.020860000 | 5.840385000 | -1.569842000 |
| C | -7.563919000 | -4.060998000 | -0.090537000 |

| | | | |
|---|---|---|---|
| C | -6.888174000 | -3.054229000 | 0.604974000 |
| C | -5.894346000 | -2.307778000 | -0.030103000 |
| C | -5.578051000 | -2.591891000 | -1.360559000 |
| C | -6.242734000 | -3.596315000 | -2.066423000 |
| C | -7.242647000 | -4.328500000 | -1.423927000 |
| H | -8.351761000 | -4.630278000 | 0.404484000 |
| H | -7.152937000 | -2.832683000 | 1.639698000 |
| H | -5.383210000 | -1.495506000 | 0.488190000 |
| H | -5.974491000 | -3.810133000 | -3.102322000 |
| H | -7.770679000 | -5.111479000 | -1.969435000 |
| C | 6.046774000 | -2.618927000 | -1.122160000 |
| C | 5.019078000 | -3.529604000 | -1.378585000 |
| C | 5.139529000 | -4.876818000 | -1.029874000 |
| C | 6.309634000 | -5.315016000 | -0.408623000 |
| C | 7.339913000 | -4.412102000 | -0.133397000 |
| C | 7.205931000 | -3.067781000 | -0.488609000 |
| H | 5.952691000 | -1.578745000 | -1.435482000 |
| H | 4.322660000 | -5.572502000 | -1.229411000 |
| H | 6.413733000 | -6.365758000 | -0.135992000 |
| H | 8.253505000 | -4.760953000 | 0.349374000 |
| H | 8.012681000 | -2.361535000 | -0.290251000 |

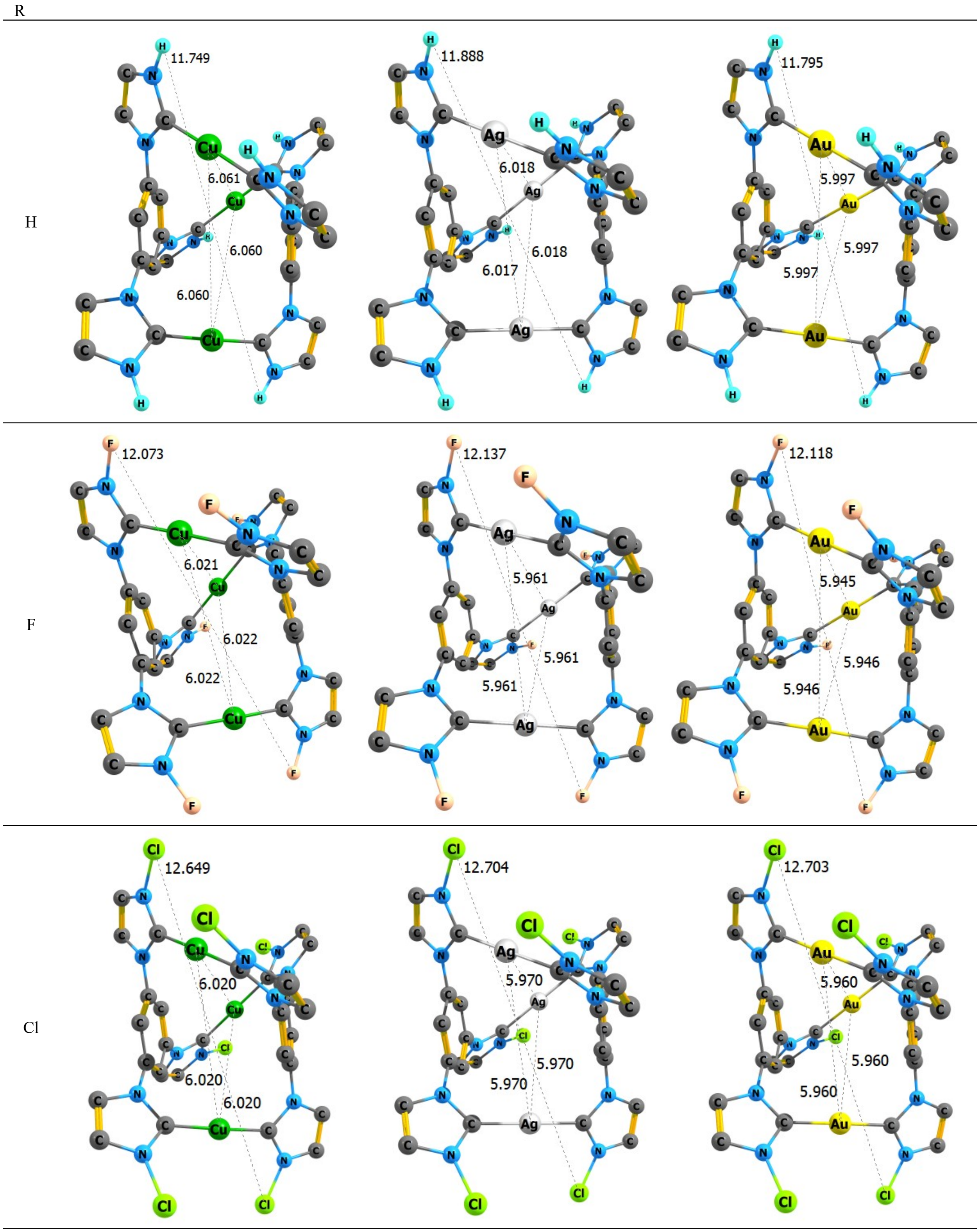


Figure S1: The optimized structures and length of $[L_2(R)_6 \rightarrow M_3]^{3+}$ complexes; M=Cu(I), Ag(I), Au(I); R = H, F and Cl. All hydrogen atoms were removed to enhance clarity.

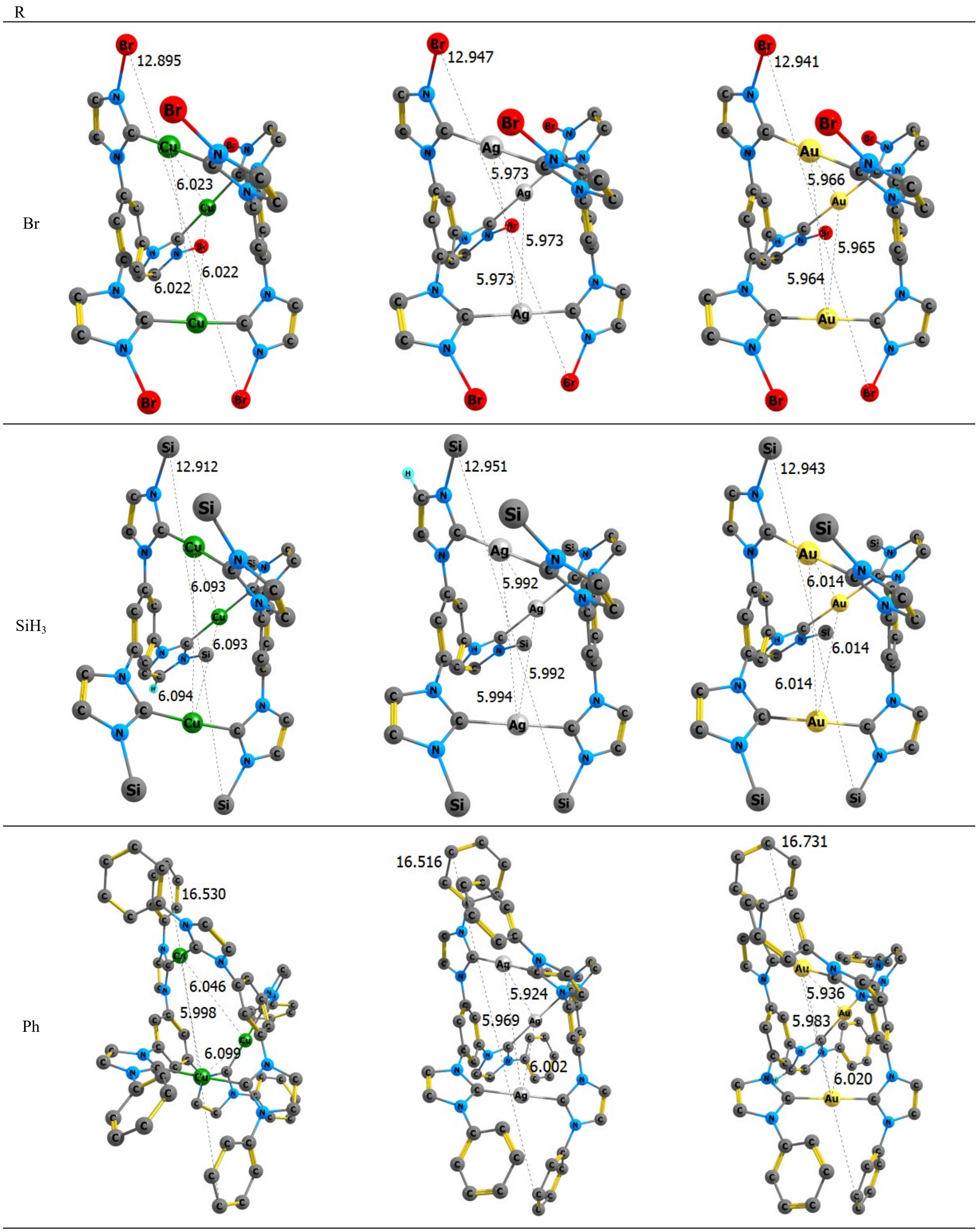


Figure S2: The optimized structures and length of $[L_2(R)_6 \rightarrow M_3]^{3+}$ complexes; M=Cu(I), Ag(I), Au(I); R = Br, $SiH_3$ and Ph. All hydrogen atoms were removed to enhance clarity.

Table S2. Wiberg Bond index of $C_{(tris\text{-}NHC)} \rightarrow M_3$ bond in $[L_2(R)_6 \rightarrow M_3]^3$ complexes

| M | Bond | Wiberg Index | | | | | | | |
|---|---|---|---|---|---|---|---|---|---|
| | | $C_2H_5$ | $CH_3$ | H | F | Cl | Br | $SiH_3$ | Ph |
| Cu | Cu-C | 0.58 | 0.58 | 0.58 | 0.56 | 0.57 | 0.57 | 0.58 | 0.58 |
| Ag | Ag-C | 0.50 | 0.52 | 0.52 | 0.51 | 0.50 | 0.50 | 0.49 | 0.50 |
| Au | Au-C | 0.61 | 0.61 | 0.61 | 0.61 | 0.61 | 0.61 | 0.61 | 0.62 |

Table S3: Most important Donor−Acceptor interactions analysis (kcal/mol) of [L2(R)6→M3]3+ nano-sized complexes.

| Donor→Accept or | Type | R | | | | | | | |
|---|---|---|---|---|---|---|---|---|---|
| | | $[L_2(R)_6 \rightarrow Cu_3]^{3+}$ | | | | | | | |
| | | $C_2H_5$ | $CH_3$ | H | F | Cl | Br | $SiH_3$ | Ph |
| $Cu \rightarrow C_{NHC}\text{-}N_4$ | LP→ σ* | -------- | 5.12 | 5.23 | 5.35 | 5.28 | 5.23 | 5.14 | |
| $C_{NHC} \rightarrow Cu$ | LP→ LP* | 156.77 | 159.51 | 158.67 | 148.29 | 150.97 | 152.23 | -------- | 154.55 |
| $C_{NHC}\text{-}N_4 \rightarrow Cu$ | σ*→ LP* | -------- | 3.20 | 3.04 | 3.23 | 3.02 | -------- | -------- | -------- |
| | | $[L_2(R)_6 \rightarrow Ag_3]^{3+}$ | | | | | | | |
| $Ag \rightarrow C_{NHC}\text{-}N_4$ | LP→ σ* | 4.11 | 4.20 | 4.18 | 4.34 | 4.27 | 4.34 | 4.17 | 4.26 |
| $C_{NHC} \rightarrow Ag$ | LP→ LP* | 158.46 | 160.33 | 157.82 | 149.18 | 154.56 | 155.36 | 160.78 | 150.35 |
| | | $L_2(R)_6 \rightarrow Au_3]^{3+}$ | | | | | | | |
| $Au \rightarrow C_{NHC}\text{-}N_4$ | σ → σ* | 4.38 | 4.20 | 4.11 | 4.46 | 4.33 | 4.31 | 4.05 | 4.18 |
| $Au \rightarrow C_{NHC}\text{-}N_4$ | LP→ σ* | 7.58 | 7.70 | 7.97 | 8.32 | 8.41 | 8.30 | 7.76 | 7.91 |
| $C_{NHC} \rightarrow Au$ | LP→ LP* | 28.48 | 32.37 | 26.98 | 26.87 | 31.33 | 33.14 | 33.17 | 33.79 |
| $C_{NHC} \rightarrow Au\text{-}C_{NHC}$ | LP→ σ* | 161.34 | 164.87 | 162.37 | 162.05 | 166.98 | 167.82 | 169.71 | 161.57 |

| | Component A-B of $[L_2(C_2H_5)_6 \rightarrow Cu_3]^{3+}$ | Component A-BA′ of $[L_2(C_2H_5)_6 \rightarrow Cu_3]^{3+}$ |
|---|---|---|
| Δρ1 | ΔE =-37.63 Kcal/Mol<br>v:0.68983<br>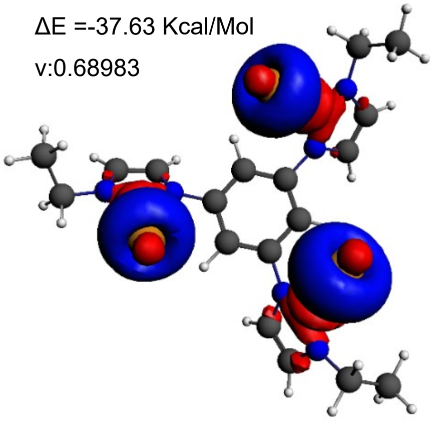 | ΔE =-27.99 Kcal/Mol<br>v: 0.42806<br>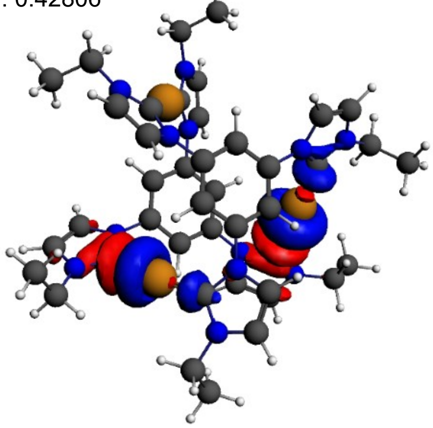 |
| Δρ2 | ΔE =-37.48 Kcal/Mol<br>v:0.68326<br>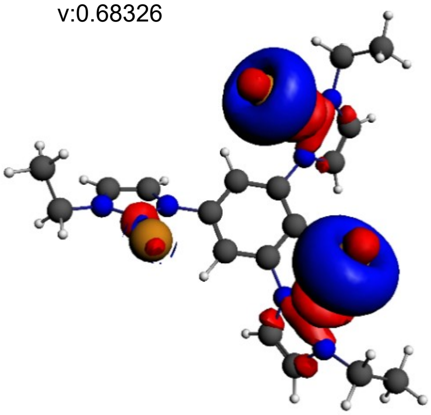 | ΔE =-27.92 Kcal/Mol<br>v:0.42773<br>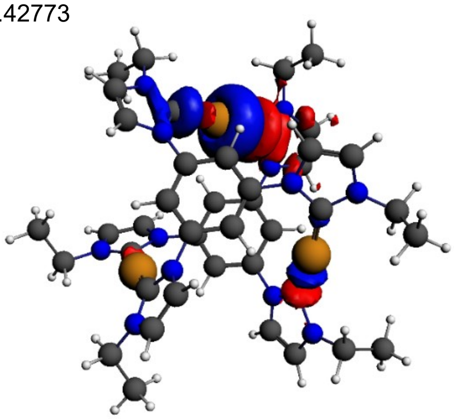 |
| Δρ3 | ΔE =-37.45 Kcal/Mol<br>v:0.68243<br>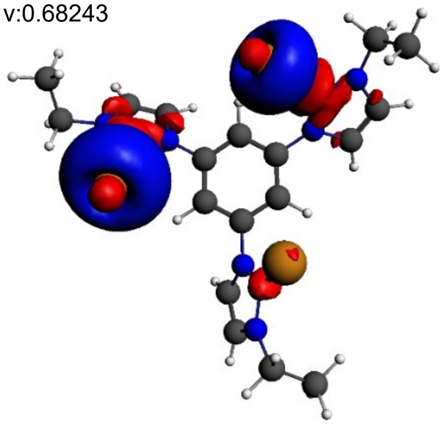 | ΔE =-27.94 Kcal/Mol<br>v:0.42710<br>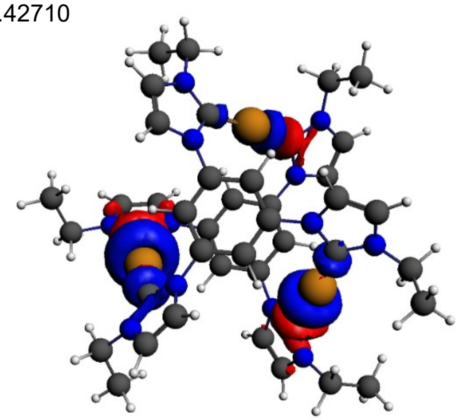 |

Component A-B of $[L_2(CH_3)_6 \rightarrow Cu_3]^{3+}$ | Component A-BA′ of $[L_2(CH_3)_6 \rightarrow Cu_3]^{3+}$

$\Delta\rho1$ ΔE =-36.94 Kcal/Mol

v:0.67887

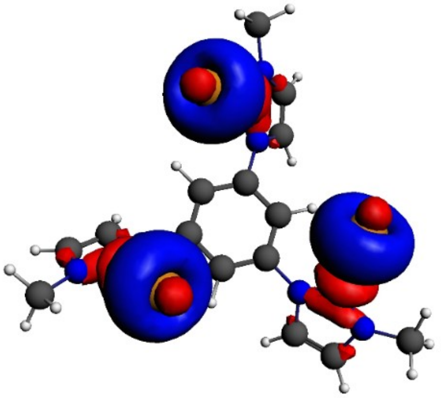

ΔE =-28.06 Kcal/Mol

v:0.42744

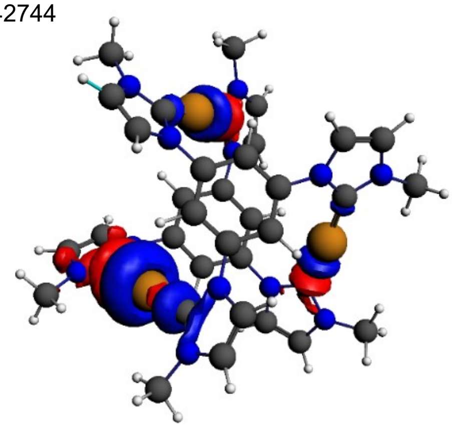

$\Delta\rho2$ ΔE =-36.80 Kcal/Mol

v: 0.67159

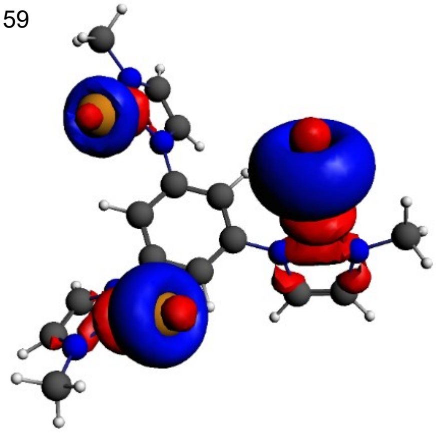

ΔE =-28.06 Kcal/Mol

v:0.42739

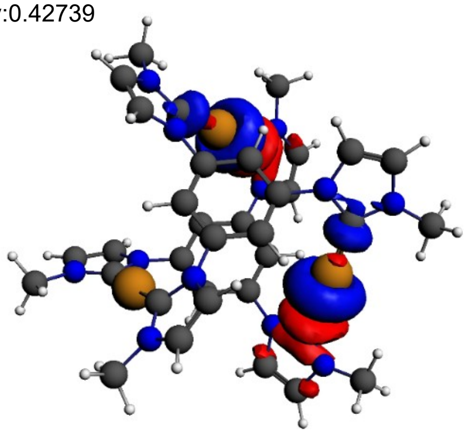

$\Delta\rho3$ ΔE =-36.79 Kcal/Mol

v:0.67144

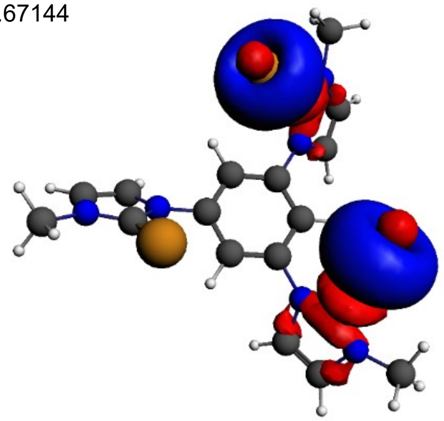

ΔE =-28.08Kcal/Mol

v:0.42684

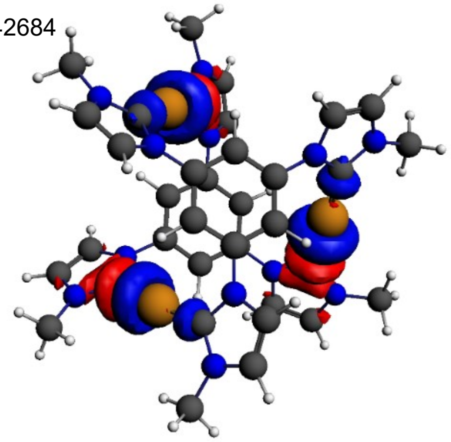

| | Component A-B of $[L_2(H)_6 \rightarrow Cu_3]^{3+}$ | Component A-BA′ of $[L_2(H)_6 \rightarrow Cu_3]^{3+}$ |
| --- | --- | --- |
| Δρ1 | ΔE =-36.76 Kcal/Mol<br>v:0.67686<br>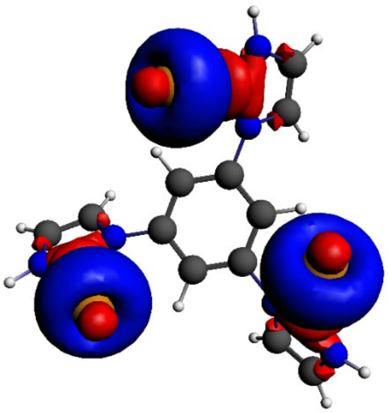 | ΔE =-28.012 Kcal/Mol<br>v:0.42367<br>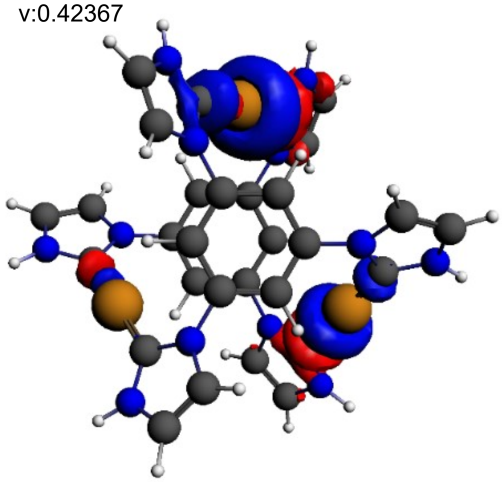 |
| Δρ2 | ΔE =-36.58 Kcal/Mol<br>v:0.67031<br>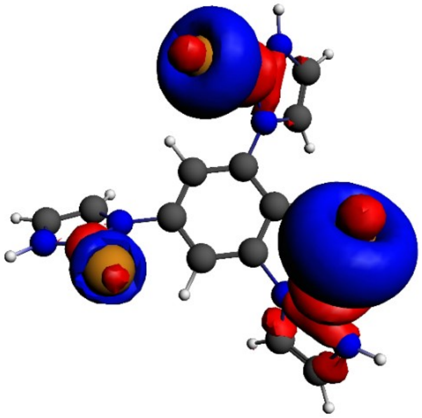 | ΔE =-28.01 Kcal/Mol<br>v:0.42366<br>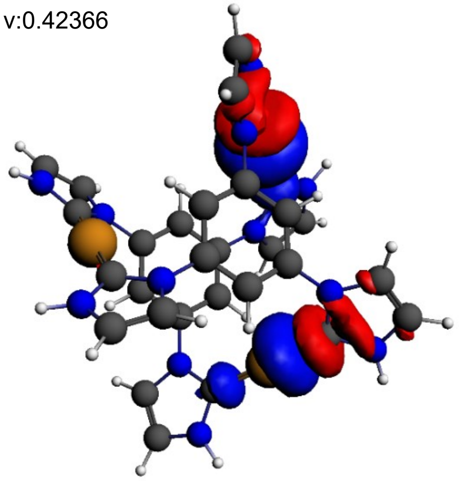 |
| Δρ3 | ΔE =-36.58 Kcal/Mol<br>v:0.67019<br>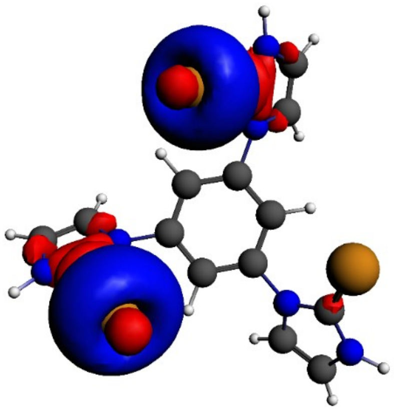 | ΔE =-28.047 Kcal/Mol<br>v:0.42343<br>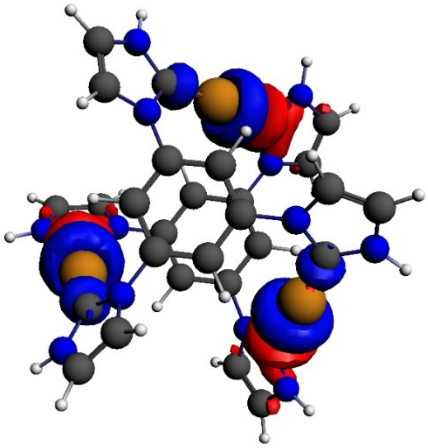 |

| | Component A-B of $[L_2(F)_6 \rightarrow Cu_3]^{3+}$ | Component A-BA′ of $[L_2(F)_6 \rightarrow Cu_3]^{3+}$ |
|---|---|---|
| Δρ1 | ΔE =-36.45 Kcal/Mol<br>v:0.66766<br>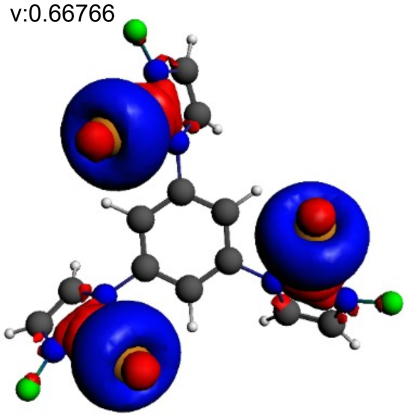 | ΔE =-29.14 Kcal/Mol<br>v:0.43816<br>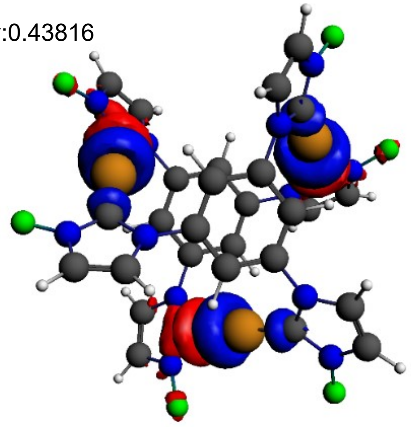 |
| Δρ2 | ΔE =-36.24 Kcal/Mol<br>v:0.66007<br>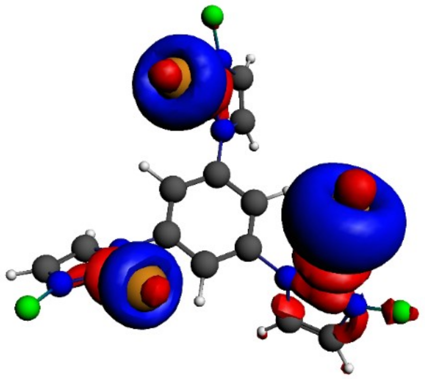 | ΔE =-28.98 Kcal/Mol<br>v: 0.43648<br>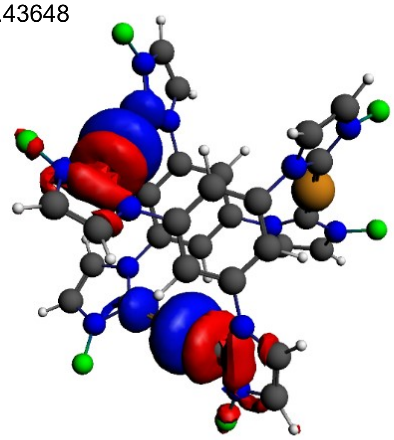 |
| Δρ3 | ΔE =-36.23 Kcal/Mol<br>v:0.66001<br>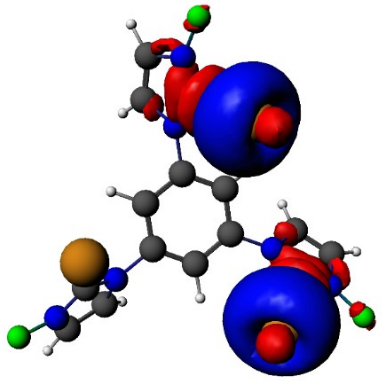 | ΔE =-28.98 Kcal/Mol<br>v:0.43642<br>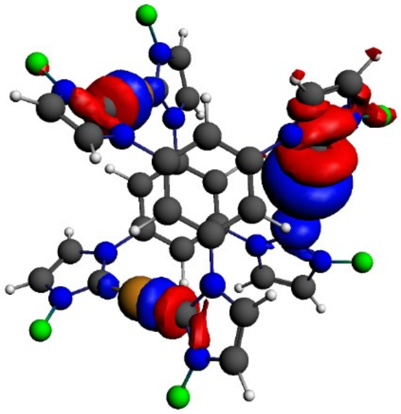 |

| | Component A-B of $[L_2(Cl)_6 \rightarrow Cu_3]^{3+}$ | Component A-BA′ of $[L_2(Cl)_6 \rightarrow Cu_3]^{3+}$ |
|---|---|---|
| Δρ1 | ΔE =-37.19 Kcal/Mol<br>v:0.68414<br>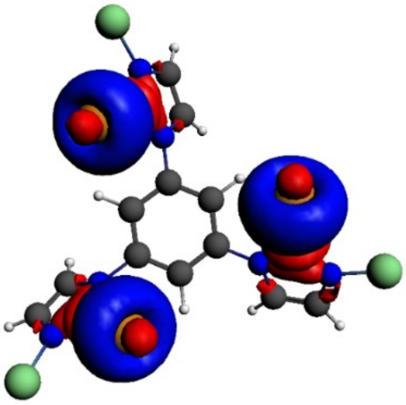 | ΔE =-28.63 Kcal/Mol<br>v:0.44059<br>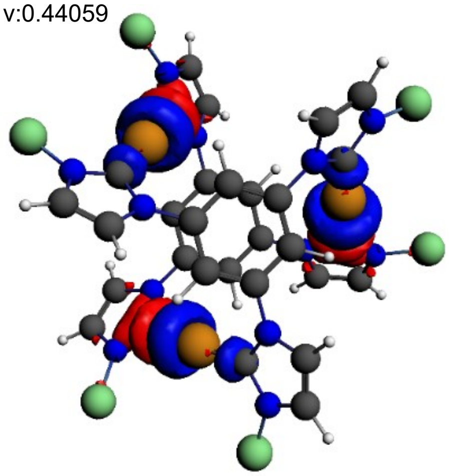 |
| Δρ2 | ΔE =-36.94 Kcal/Mol<br>v:0.67609<br>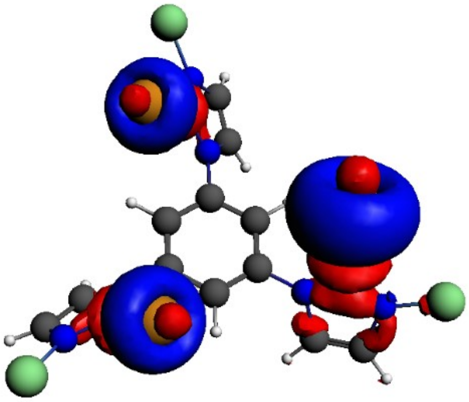 | ΔE =-28.450 Kcal/Mol<br>v:0.43916<br>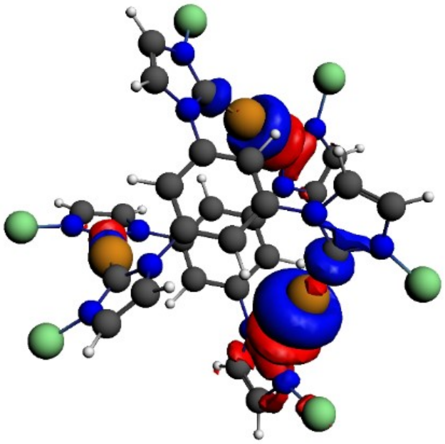 |
| Δρ3 | ΔE =-36.92 Kcal/Mol<br>v:0.67576<br>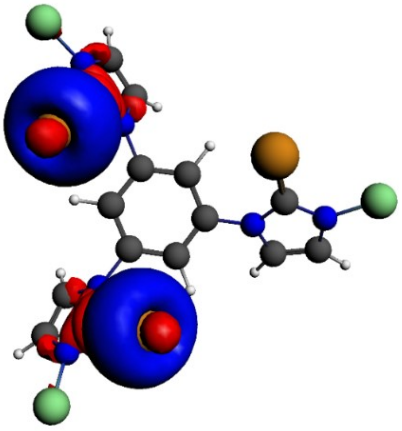 | ΔE =-28.49 Kcal/Mol<br>v:0.43916<br>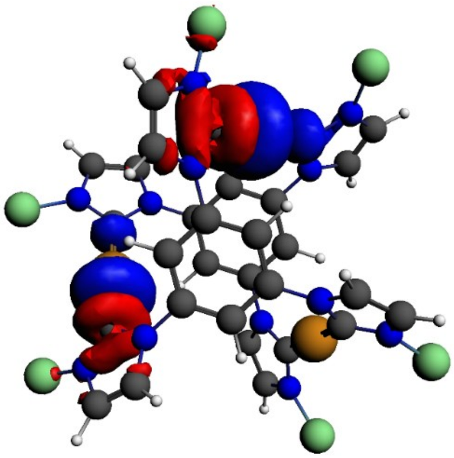 |

| | Component A-B of $[L_2(Br)_6 \rightarrow Cu_3]^{3+}$ | Component A-BA′ of $[L_2(Br)_6 \rightarrow Cu_3]^{3+}$ |
|---|---|---|
| Δρ1 | ΔE =-38.45 Kcal/Mol<br>v:0.69939<br>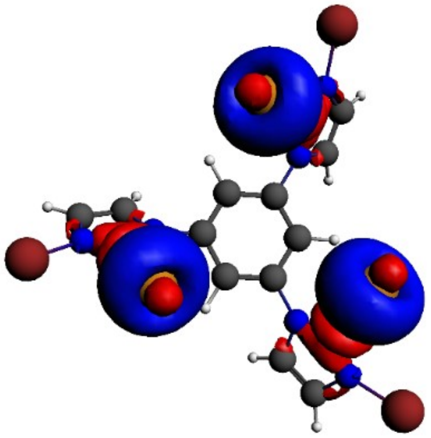 | ΔE =-29.24 Kcal/Mol<br>v:0.45606<br>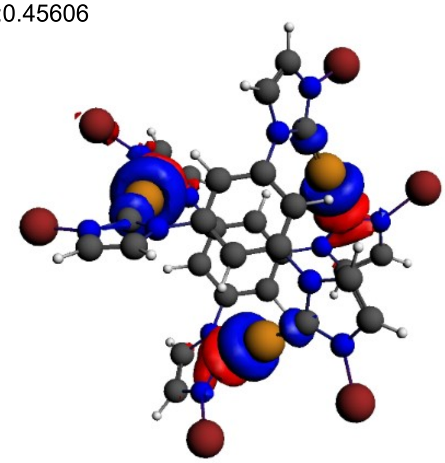 |
| Δρ2 | ΔE =-38.11 Kcal/Mol<br>v:0.69041<br>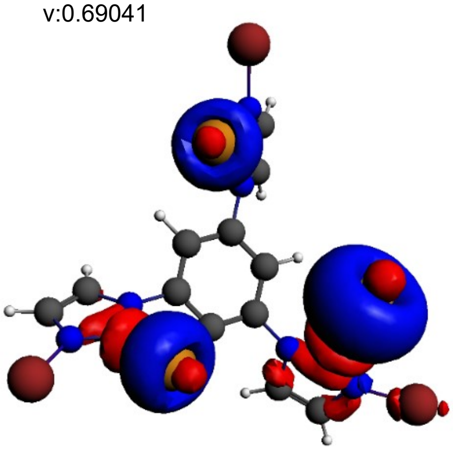 | ΔE =-29.03 Kcal/Mol<br>v:0.45358<br>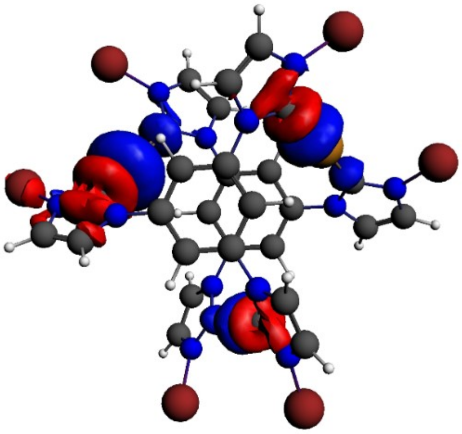 |
| Δρ3 | ΔE =-38.09 Kcal/Mol<br>v:0.69025<br>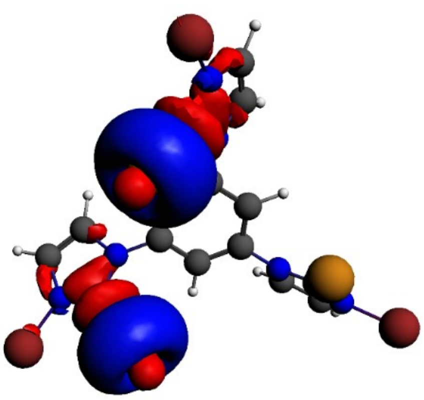 | ΔE =-29.01 Kcal/Mol<br>v:0.45323<br>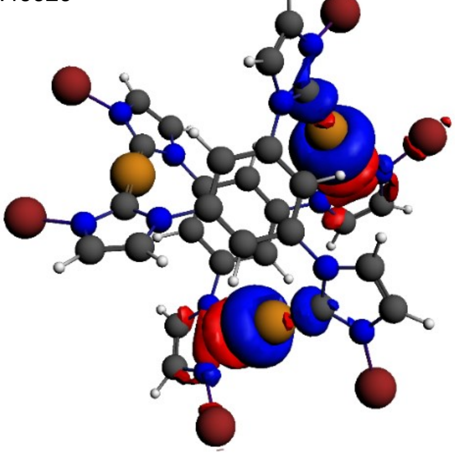 |

| | Component A-B of $[L_2(SiH_3)_6 \rightarrow Cu_3]^{3+}$ | Component A-BA′ of $[L_2(SiH_3)_6 \rightarrow Cu_3]^{3+}$ |
|---|---|---|
| Δρ1 | ΔE =-37.34 Kcal/Mol<br>v:0.68435<br>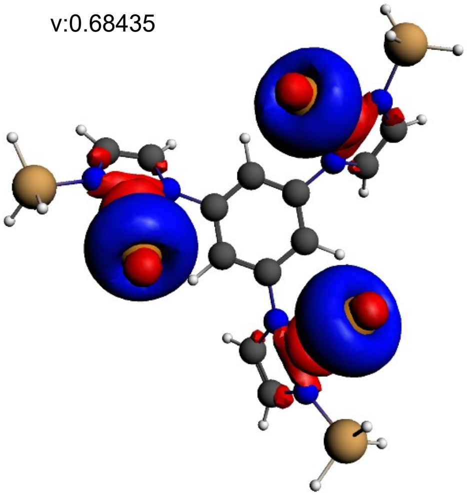 | ΔE =-27.92 Kcal/Mol<br>0.42795:v<br>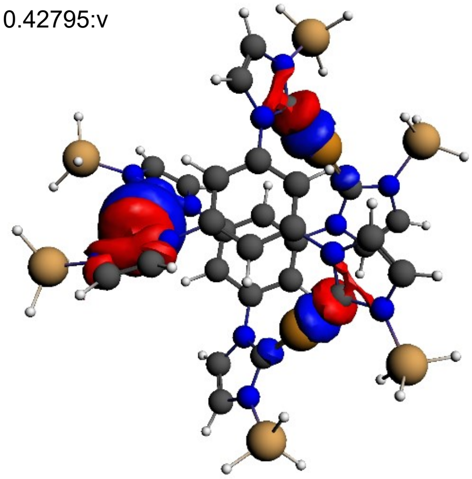 |
| Δρ2 | ΔE =-37.19 Kcal/Mol<br>v:0.67709<br>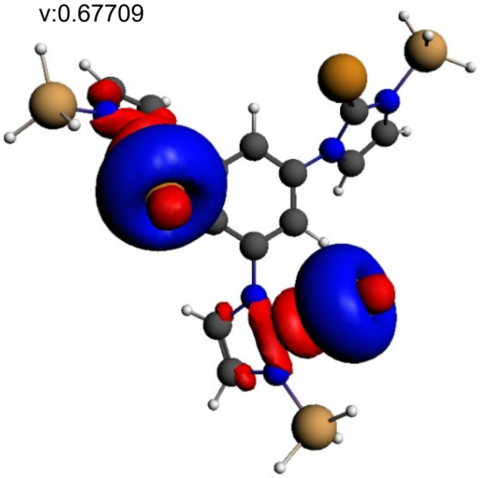 | ΔE =-27.93 Kcal/Mol<br>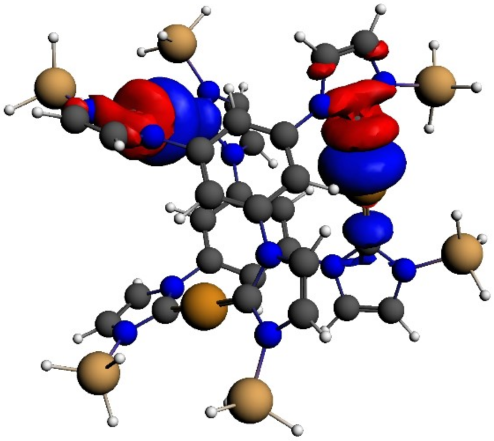 |
| Δρ3 | ΔE =-37.18 Kcal/Mol<br>v:0.67689<br>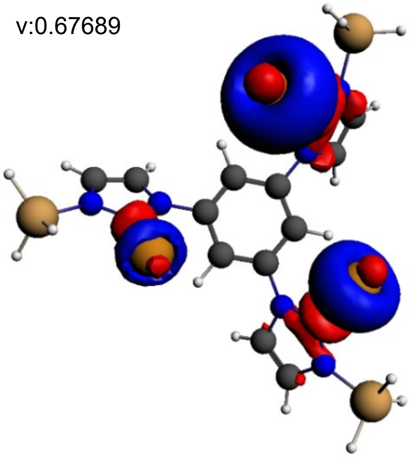 | ΔE =-27.92 Kcal/Mol<br>v:0.42712<br>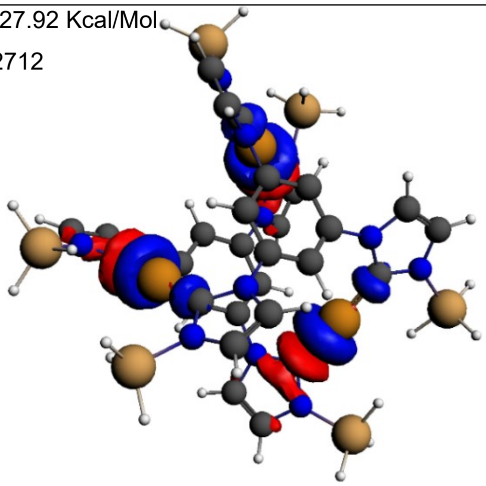 |

| | Component A-B of $[L_2(SiH_3)_6\rightarrow Cu_3]^{3+}$ | Component A-BA′ of $[L_2(SiH_3)_6\rightarrow Cu_3]^{3+}$ |
|---|---|---|
| $\Delta\rho 1$ | $\Delta E$ =-37.02 Kcal/Mol<br>v:0.68827<br>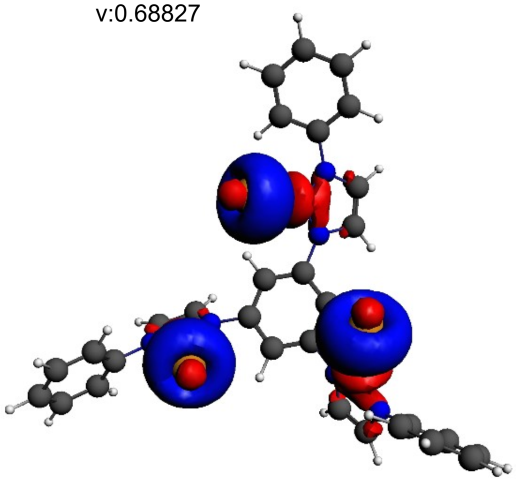 | $\Delta E$ =-27.25 Kcal/Mol<br>v:0.42988<br>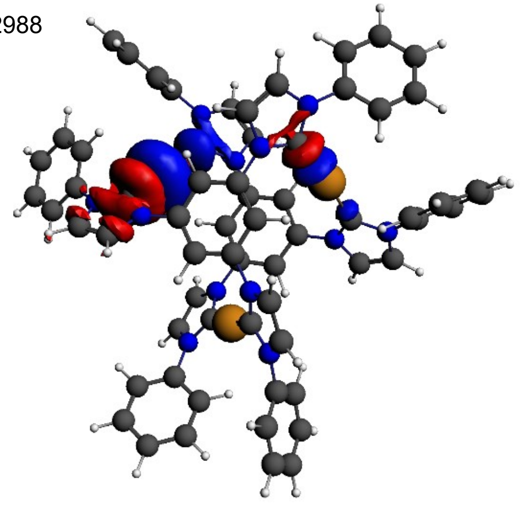 |
| $\Delta\rho 2$ | $\Delta E$ =-36.85 Kcal/Mol<br>v: 0.68066<br>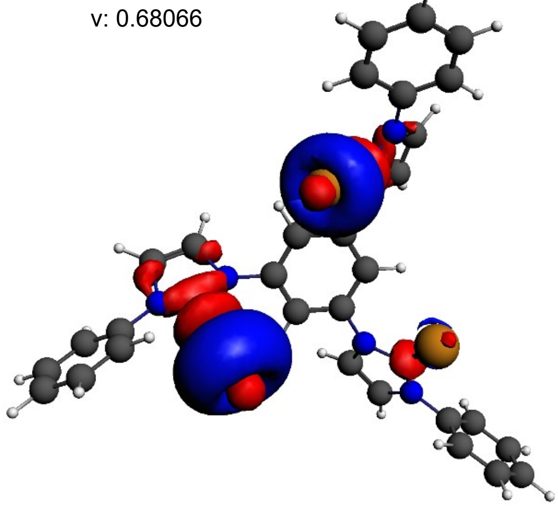 | $\Delta E$ =-27.18 Kcal/Mol<br>v:0.42864<br>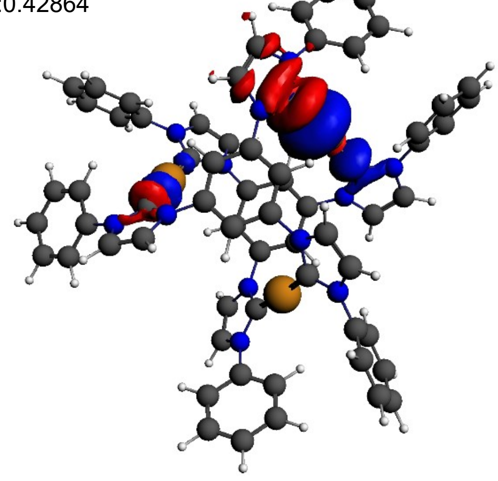 |
| $\Delta\rho 3$ | $\Delta E$ =-36.84 Kcal/Mol<br>v:0.67895<br>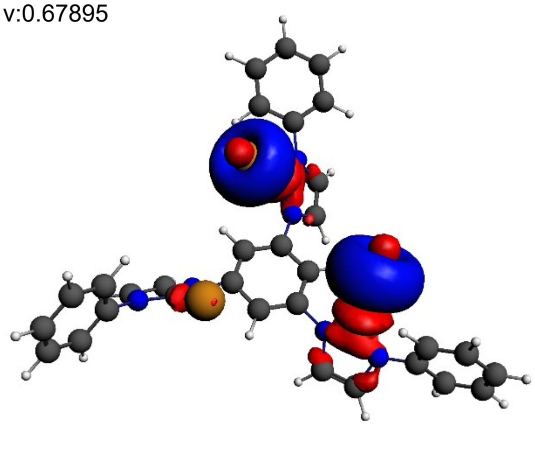 | $\Delta E$ =-27.08 Kcal/Mol<br>v:0.42476<br>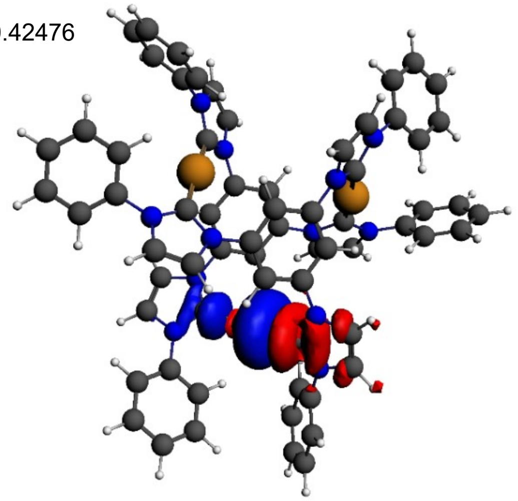 |

Figure S3: The density of shape change between the Fragments $M^{3+}$ and $L_2(R)_6$, along with the significant energy results of $[L_2(R)_6\rightarrow Cu_3]^{3+}$; R=$C_2H_5$, $CH_3$, H, F, Cl, Br, $SiH_3$ and Ph complexes for A-B and A-BA′ components.

| | Component A-B of $[L_2(C_2H_5)_6 \rightarrow Ag_3]^{3+}$ | Component A-BA′ of $[L_2(C_2H_5)_6 \rightarrow Ag_3]^{3+}$ |
|---|---|---|
| $\Delta\rho_1$ / $\Delta\rho_1$ | ΔE = -35.03 Kcal/mol, v: 0.63762 / ΔE = -35.35 Kcal/Mol, v: 0.64246 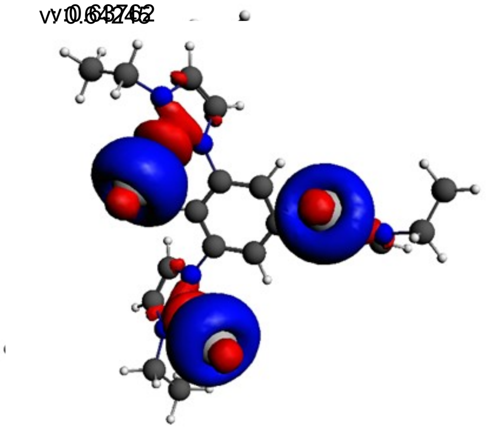 | ΔE = -26.70 Kcal/Mol, v: 0.45694 / ΔE = -26.64 Kcal/Mol, v: 0.46147 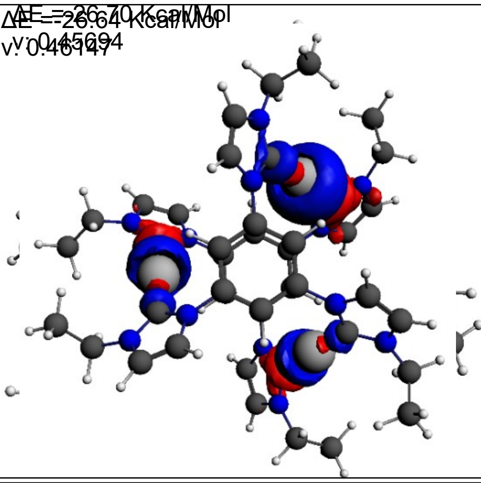 |
| $\Delta\rho_2$ / $\Delta\rho_2$ | ΔE = -34.66 Kcal/Mol, v: 0.62670 / ΔE = -34.96 Kcal/Mol, v: 0.63441 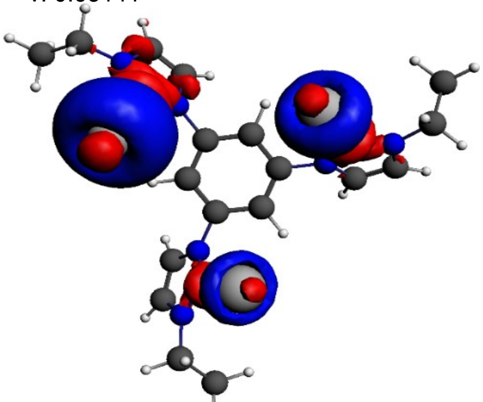 | ΔE = -26.66 Kcal/Mol, v: 0.45673 / ΔE = -26.60 Kcal/Mol, v: 0.45692 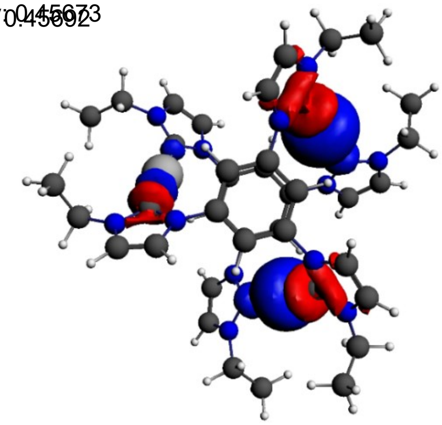 |
| $\Delta\rho_3$ / $\Delta\rho_3$ | ΔE = -34.36 Kcal/Mol, v: 0.62664 / ΔE = -34.95 Kcal/Mol, v: 0.63422 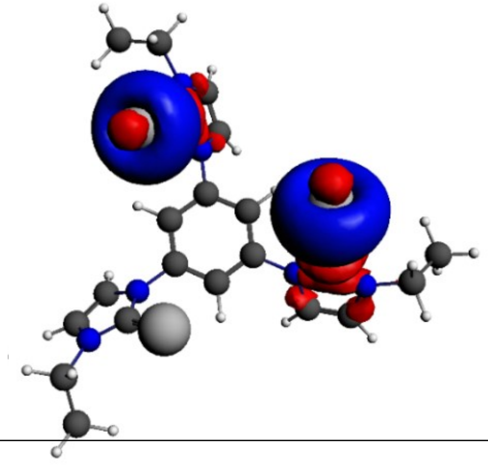 | ΔE = -26.65 Kcal/Mol, v: 0.45668 / ΔE = -26.59 Kcal/Mol, v: 0.45683 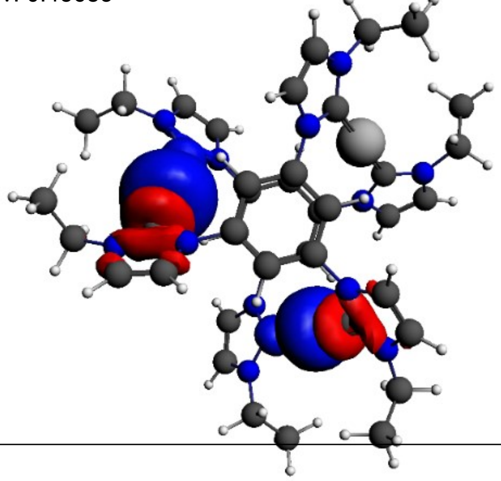 |

Component A-B of $[L_2(CH_3)_6{\rightarrow}Ag_3]^{3+}$

Component A-BA′ of $[L_2(CH_3)_6{\rightarrow}Ag_3]^{3+}$

| | | |
|---|---|---|
| $\Delta\rho 1$ | $\Delta E$ =-34.27 Kcal/mol<br>v:0.62884<br>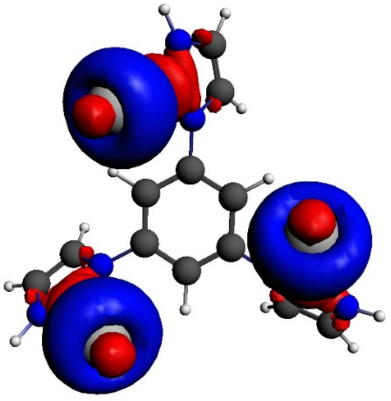 | $\Delta E$ =-26.65 Kcal/mol<br>v: 0.45694<br>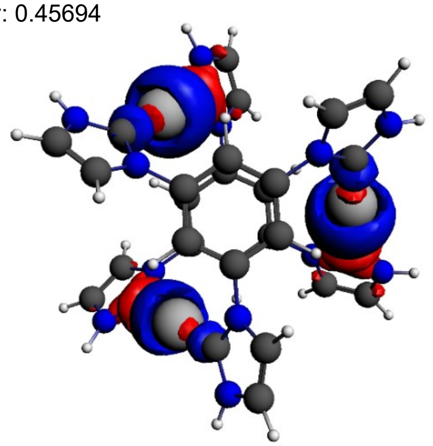 |
| $\Delta\rho 2$ | $\Delta E$ =-33.88 Kcal/mol<br>v: 0.61783<br>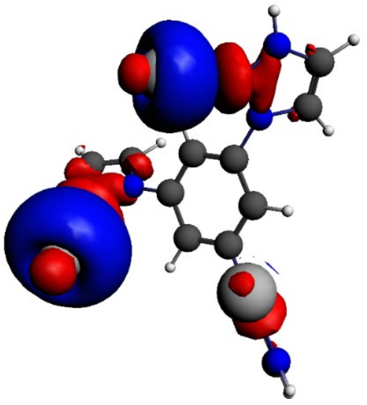 | $\Delta E$ =-26.59 Kcal/mol<br>v: 0.45636<br>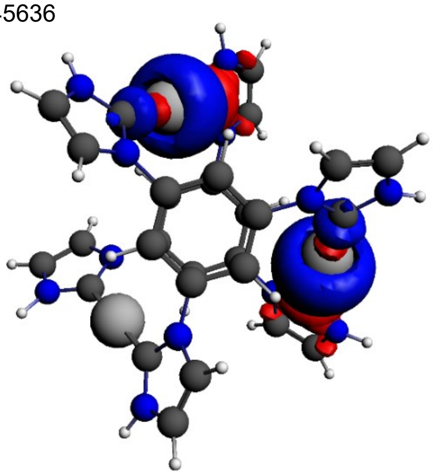 |
| | Component A-B of $[L_2(H)_6\rightarrow Ag_3]^{3+}$ | Component A-BA′ of $[L_2(H)_6\rightarrow Ag_3]^{3+}$ |

Δρ3 ΔE =-33.88 Kcal/mol
v: 0.61780

ΔE =-26.59 Kcal/mol
v: 0.45627

Δρ1 ΔE =-33.52 Kcal/mol
v: 0.61757

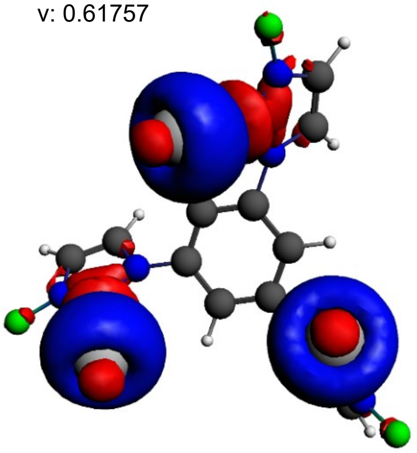

ΔE =-26.86 Kcal/mol
v:0.46219

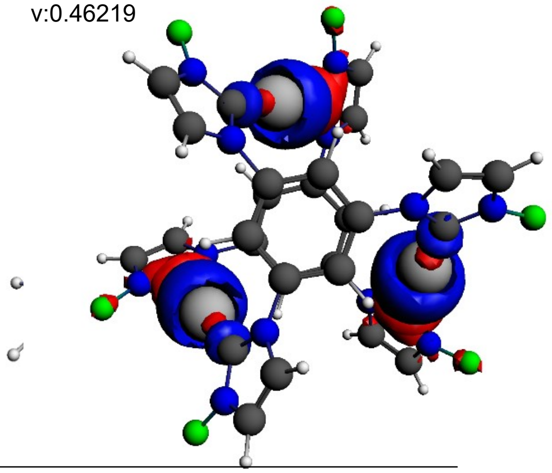

Δρ2 ΔE =-33.10 Kcal/mol
v: 0.60528

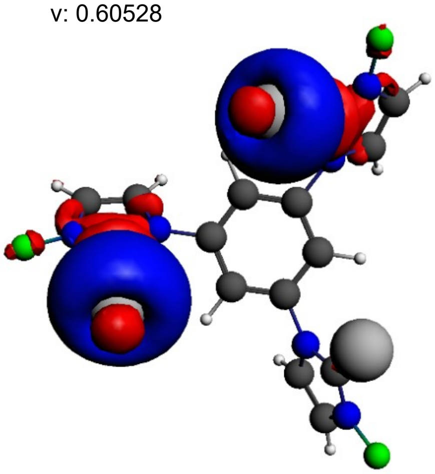

ΔE =-26.72 Kcal/mol
v: 0.45937

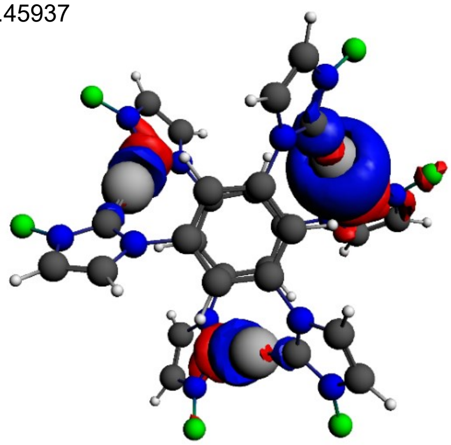

Δρ3 ΔE =-33.10 Kcal/mol
v: 0.60518

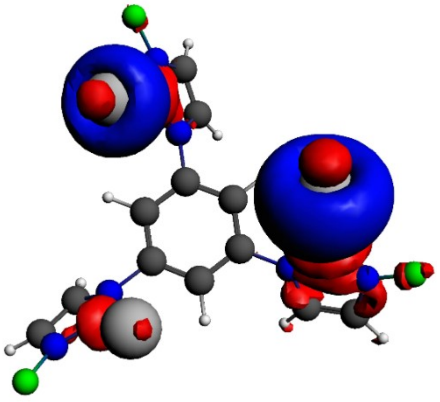

ΔE =-26.70 Kcal/mol
v: 0.45924

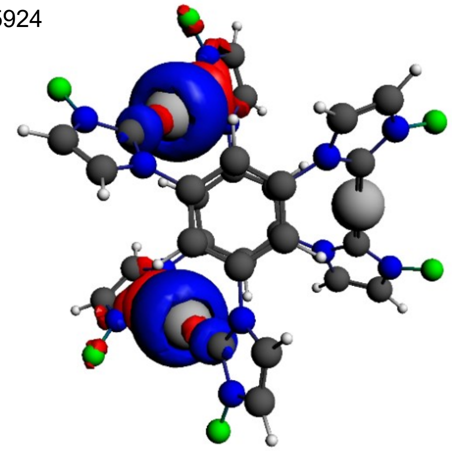

Component A-B of $[L_2(F)_6 \rightarrow Ag_3]^{3+}$

Component A-BA′ of $[L_2(F)_6 \rightarrow Ag_3]^{3+}$

Component A-B of $[L_2(Cl)_6 \rightarrow Ag_3]^{3+}$

Component A-BA′ of $[L_2(Cl)_6 \rightarrow Ag_3]^{3+}$

Δρ1 ΔE =-33.83 Kcal/mol
Δρ1 ΔE =-35.56 Kcal/mol
v: 0.62783
v: 0.65137

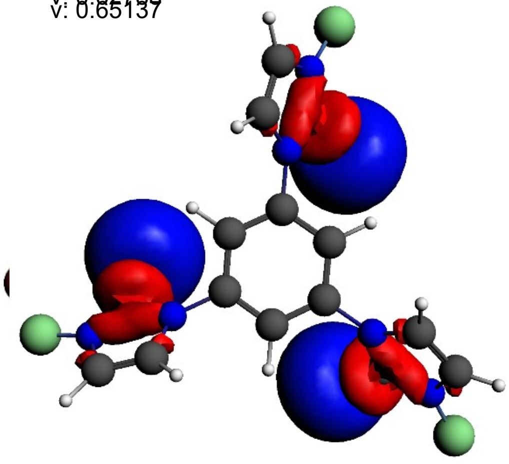

ΔE =-26.41 Kcal/mol
ΔE =-27.34 Kcal/mol
v: 0.46514
v:0.48300

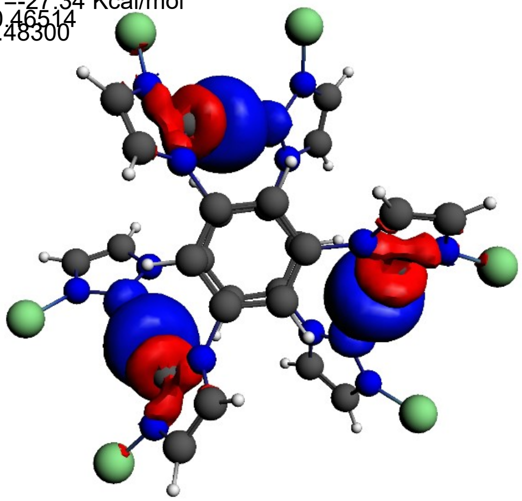

Δρ2 ΔE =-33.45 Kcal/mol
Δρ2 ΔE =-35.02 Kcal/mol
v: 0.61613
v: 0.63743

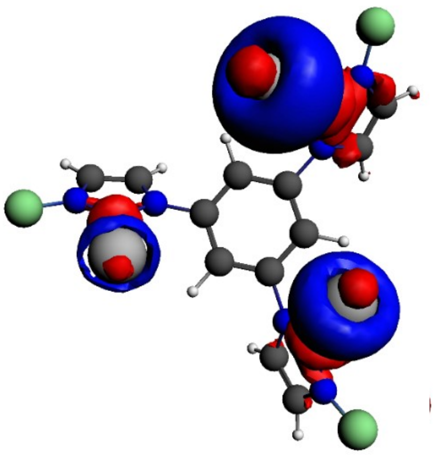

ΔE =-26.30 Kcal/mol
ΔE =-27.15 Kcal/mol
v: 0.46136
v: 0.47963

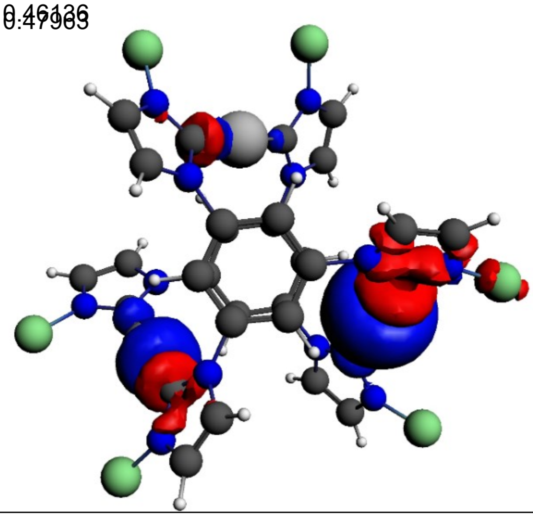

Δρ3 ΔE =-35.01 Kcal/mol
Δρ3 ΔE =-33.43 Kcal/mol
v: 0.63735
v: 0.61604

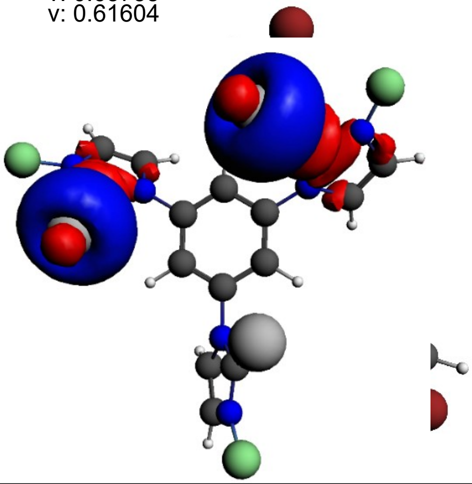

ΔE =-27.13 Kcal/mol
ΔE =-26.30 Kcal/mol
v: 0.47927
v: 0.46126

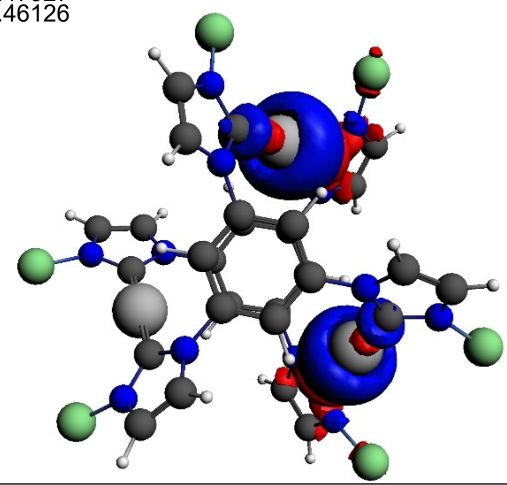

Component A-B of $[L_2(Br)_6 \rightarrow Ag_3]^{3+}$

Component A-BA′ of $[L_2(Br)_6 \rightarrow Ag_3]^{3+}$

| | Component A-B of $[L_2(SiH_3)_6 \rightarrow Ag_3]^{3+}$ | Component A-BA′ of $[L_2(SiH_3)_6 \rightarrow Ag_3]^{3+}$ |
|---|---|---|
| Δρ1 | ΔE =-35.40 Kcal/mol<br>v:0.64248<br>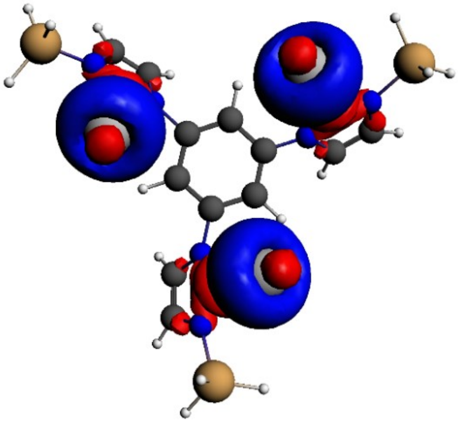 | ΔE =-26.93 Kcal/mol<br>v: 0.45968 |
| Δρ2 | ΔE =-34.99 Kcal/mol<br>v: 0.63036<br>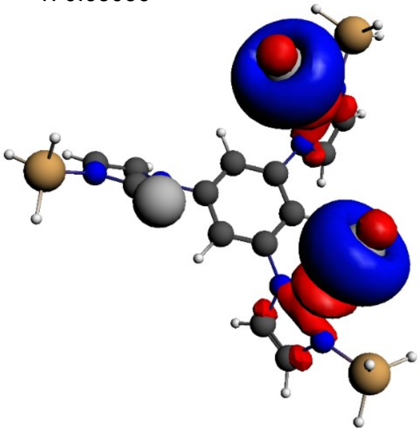 | ΔE =-26.89 Kcal/mol<br>v: 0.45902<br>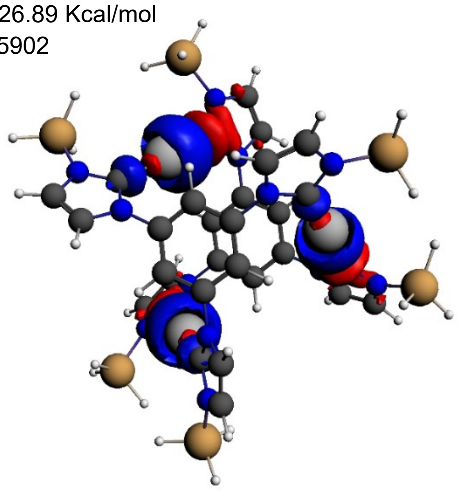 |
| Δρ3 | ΔE =-34.99 Kcal/mol<br>v: 0.63028<br>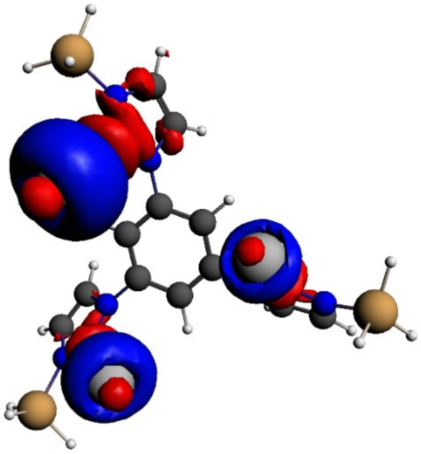 | ΔE =-26.88 Kcal/mol<br>v: 0.45895<br>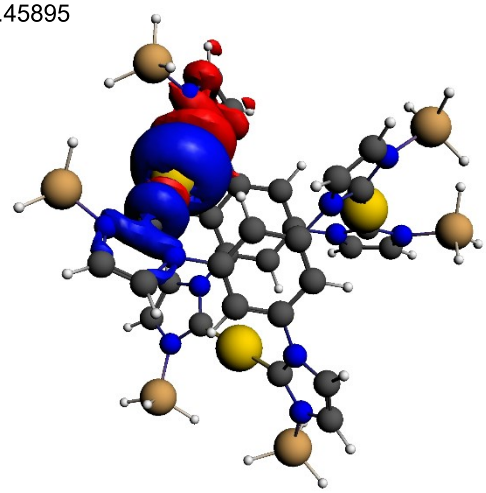 |

Δρ1 ΔE =-34.68 Kcal/mol
v: 0.64094

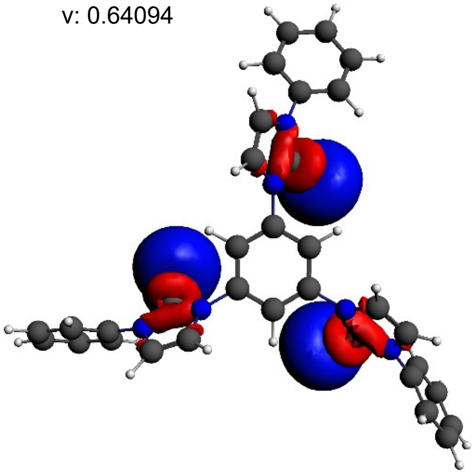

ΔE =-28.83 Kcal/mol
v: 0.47759

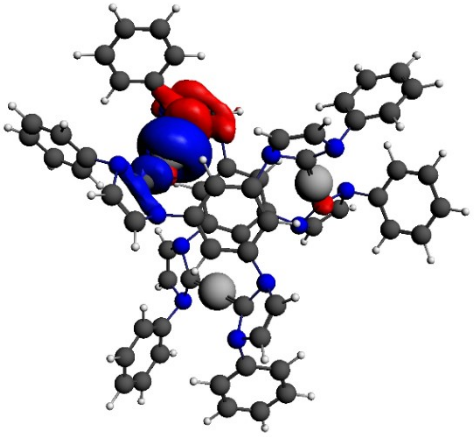

Δρ2 ΔE =-34.27 Kcal/mol
v: 0.62762

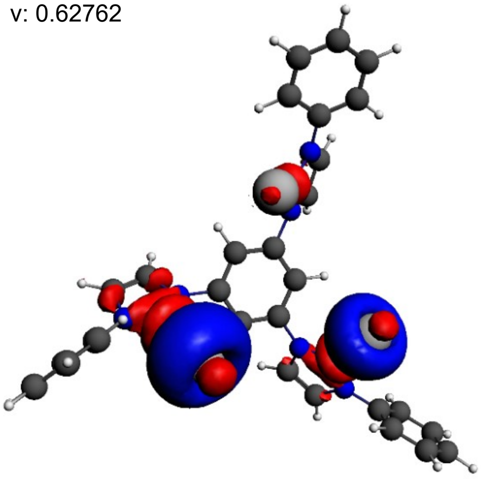

ΔE =-28.75 Kcal/mol
v: 0.47313

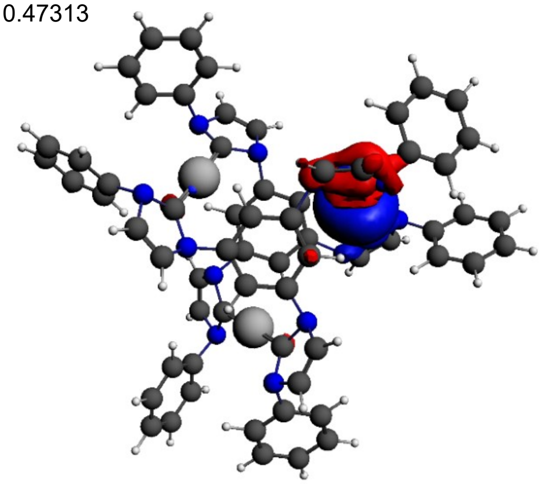

Δρ3 ΔE =-34.22 Kcal/mol
v: 0.62703

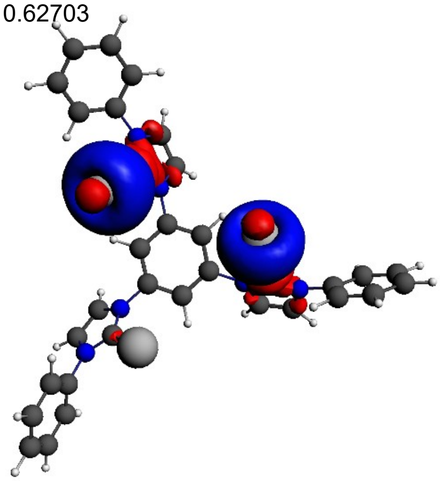

ΔE =-28.47 Kcal/mol
v: 0.46283

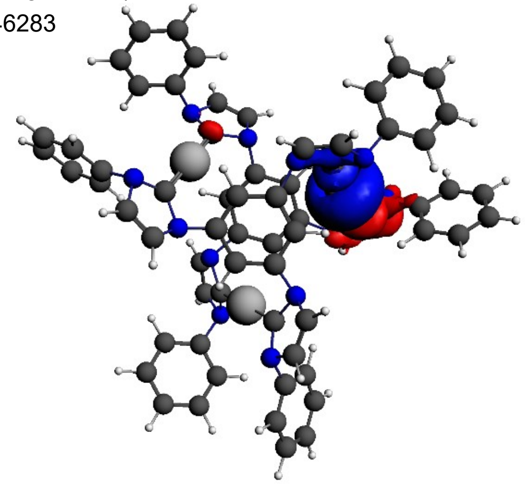

| | Component A-B of $[L_2(Ph)_6 \rightarrow Ag_3]^{3+}$ | Component A-BA′ of $[L_2(Ph)_6 \rightarrow Ag_3]^{3+}$ |
|---|---|---|
| Δρ1 | ΔE =-67.24 Kcal/mol<br>v: 0.94825<br>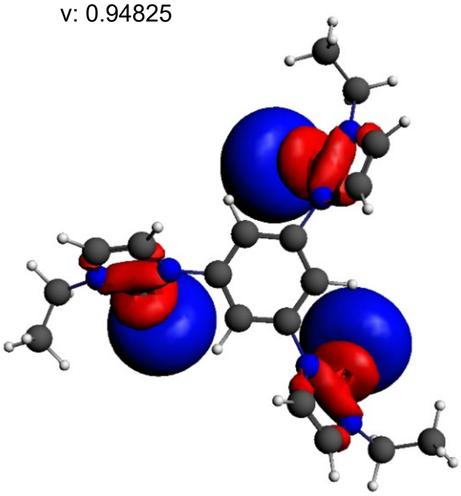 | ΔE = -44.99 Kcal/mol<br>v: 0.56493<br>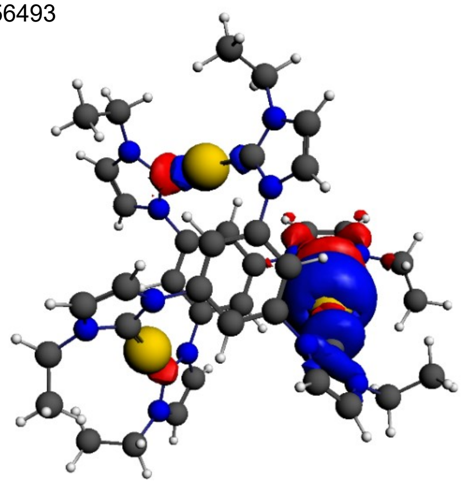 |
| Δρ2 | ΔE =-66.91 Kcal/mol<br>v: 0.94245<br>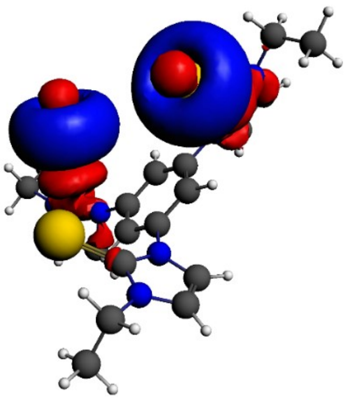 | ΔE = -44.89 Kcal/mol<br>v: 0.56476<br>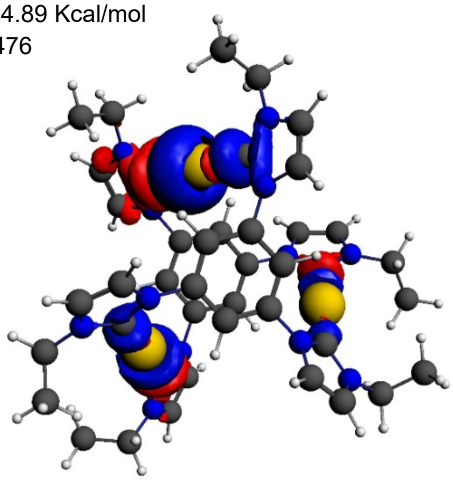 |

Figure S4: The density of shape change between the Fragments $M^{3+}$ and $L_2(R)_6$, along with the significant energy results of $[L_2(R)_6 \rightarrow Ag_3]^{3+}$; R=$C_2H_5$, $CH_3$, H, F, Cl, Br, $SiH_3$ and Ph complexes for A-B and A-BA′ components.

Component A-B of $[L_2(C_2H_5)_6 \rightarrow Au_3]^{3+}$

Component A-BA′ of $[L_2(C_2H_5)_6 \rightarrow Au_3]^{3+}$

| | | |
|---|---|---|
| $\Delta\rho3$ | $\Delta E$ =-66.86 Kcal/mol<br>v: 0.94226<br>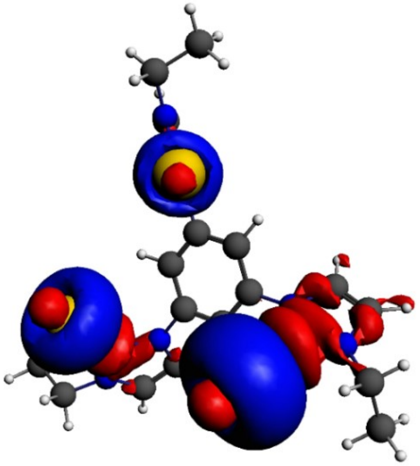 | $\Delta E$ = -44.89 Kcal/mol<br>v: 0.56473<br>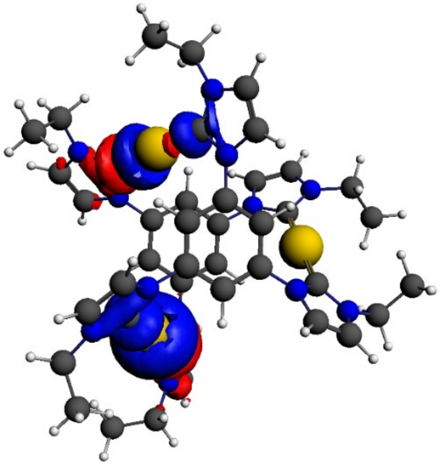 |

Component A-B of $[L_2(CH_3)_6 \rightarrow Au_3]^{3+}$

Component A-BA′ of $[L_2(CH_3)_6 \rightarrow Au_3]^{3+}$

Δρ1 ΔE =-66.68 Kcal/mol
v:0.94254

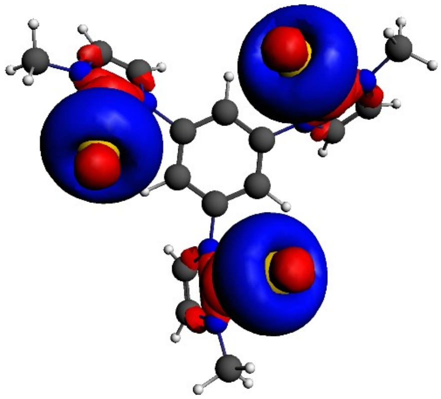

ΔE =-44.97 Kcal/mol
v: 0.56464

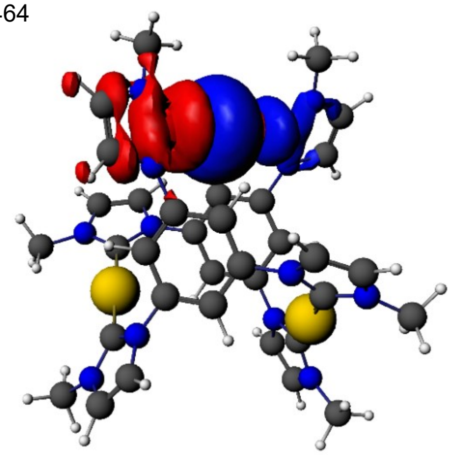

Δρ2 ΔE =-66.34 Kcal/mol
v:0.93695

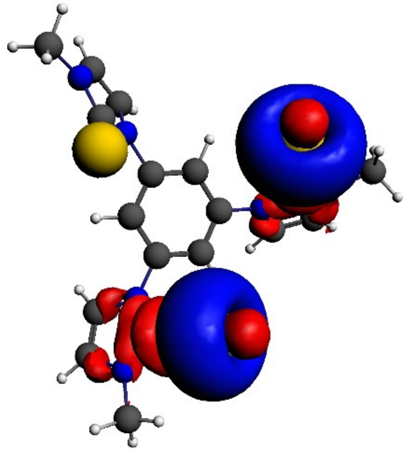

ΔE =-44.92 Kcal/mol
v: 0.56436

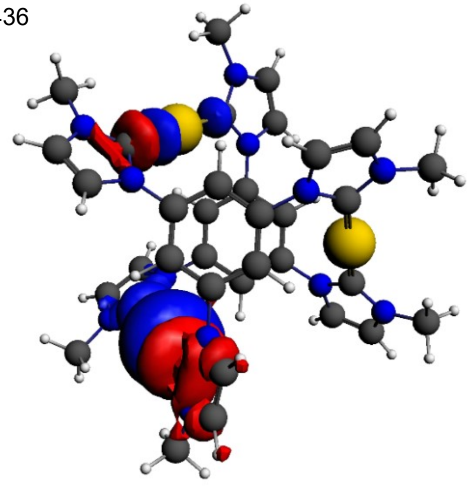

Δρ3 ΔE =-66.34 Kcal/mol
v: 0.93686

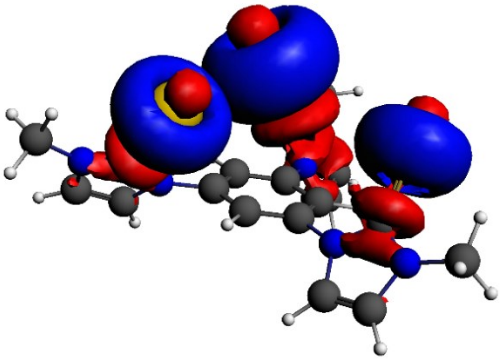

ΔE =-44.96 Kcal/mol
v: 0.56432

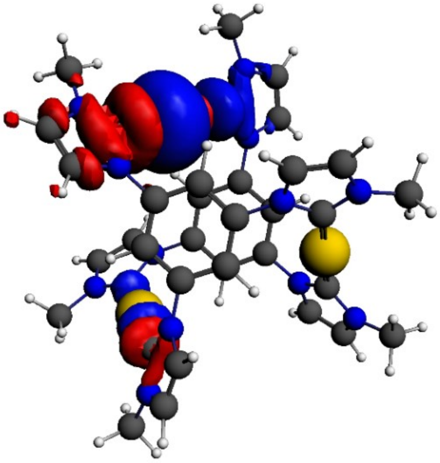

Component A-B of $[L_2(H)_6 \rightarrow Au_3]^{3+}$

Component A-BA′ of $[L_2(H)_6 \rightarrow Au_3]^{3}$

Δρ1 ΔE = -63.49 Kcal/mol
v: 0.91902

Δρ1 ΔE = -65.76 Kcal/mol
v: 0.93381

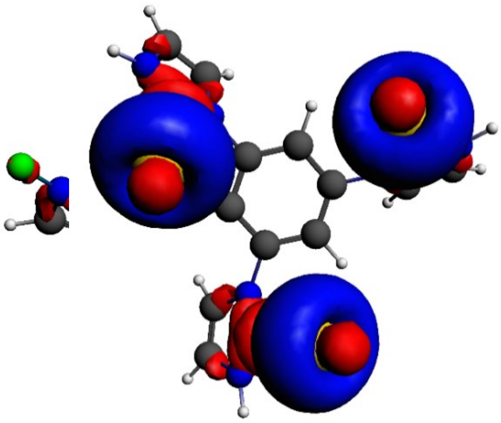

ΔE = -45.23 Kcal/mol
v: 0.57087

ΔE = -44.93 Kcal/mol
v: 0.56102

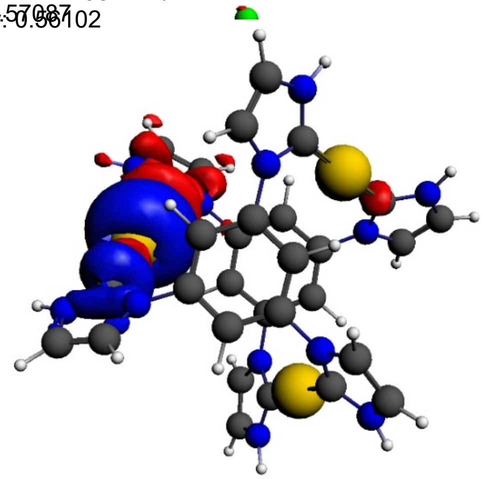

Δρ2 ΔE = -63.05 Kcal/mol
v: 0.91175

Δρ2 ΔE = -65.42 Kcal/mol
v: 0.92820

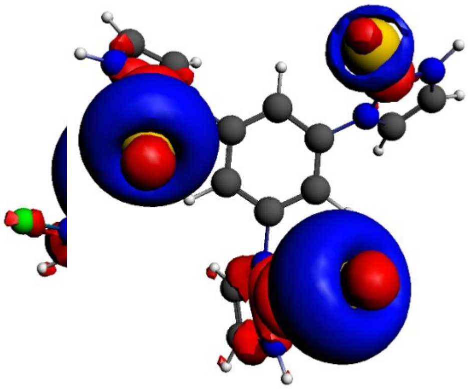

ΔE = -45.05 Kcal/mol
v: 0.56958

ΔE = -44.91 Kcal/mol
v: 0.56087

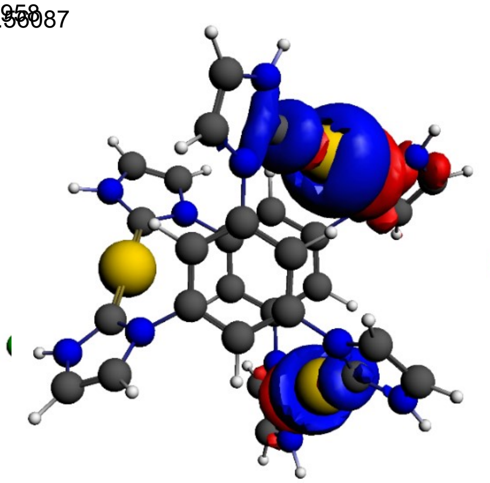

Δρ3 ΔE = -63.04 Kcal/mol
v: 0.91159

Δρ3 ΔE = -65.40 Kcal/mol
v: 0.92811

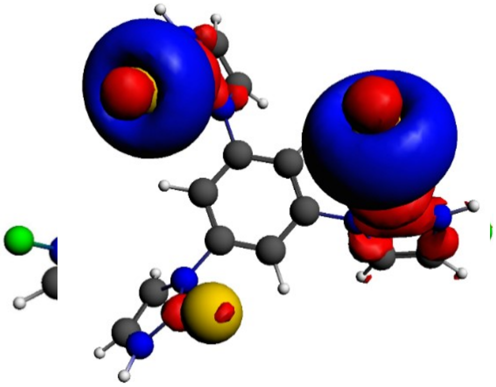

ΔE = -45.04 Kcal/mol
v: 0.56938

ΔE = -44.97 Kcal/mol
v: 0.56077

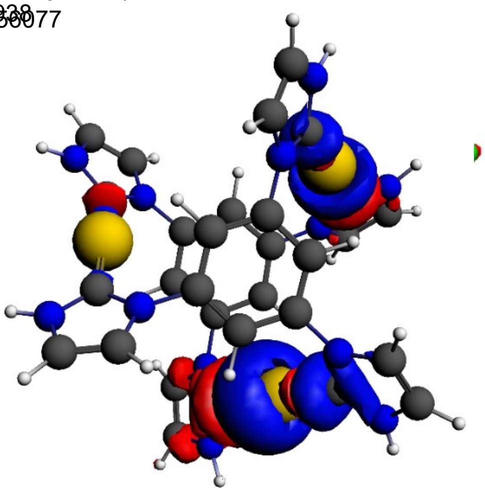

Component A-B of $[L_2(F)_6 \rightarrow Au_3]^{3+}$

Component A-BA′ of $[L_2(F)_6 \rightarrow Au_3]^{3+}$

Component A-B of $[L_2(Cl)_6{\rightarrow}Au_3]^{3+}$

Component A-BA′ of $[L_2(Cl)_6{\rightarrow}Au_3]^{3+}$

Δρ1 ΔE =-65.19 Kcal/mol
v:0.93896

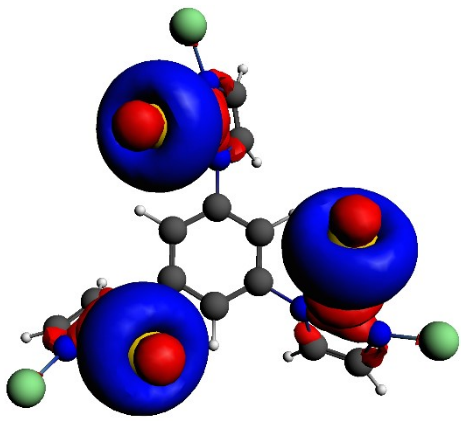

ΔE =-45.57 Kcal/mol
v:0.57349

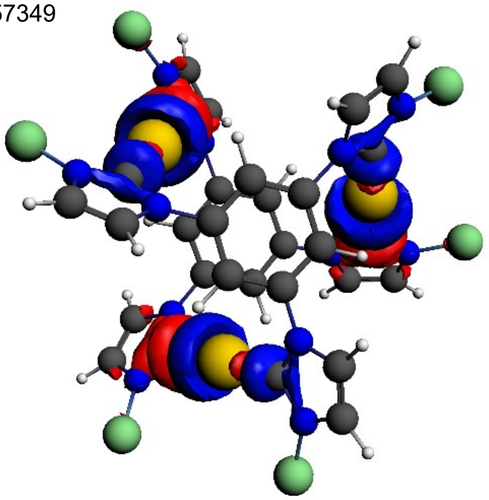

Δρ2 ΔE =-64.71 Kcal/mol
v:0.93118

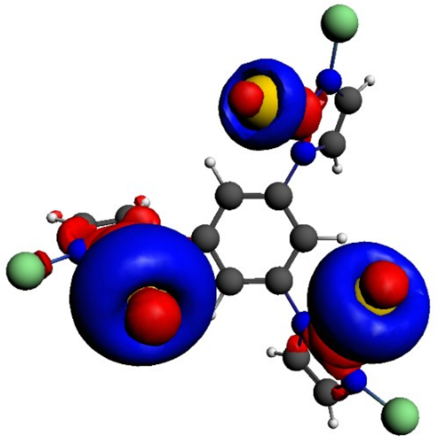

ΔE =-45.40 Kcal/mol
v:0.57224

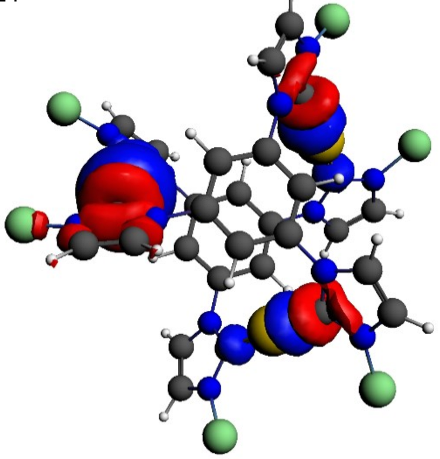

Δρ3 ΔE =-64.68 Kcal/mol
v:0.93106

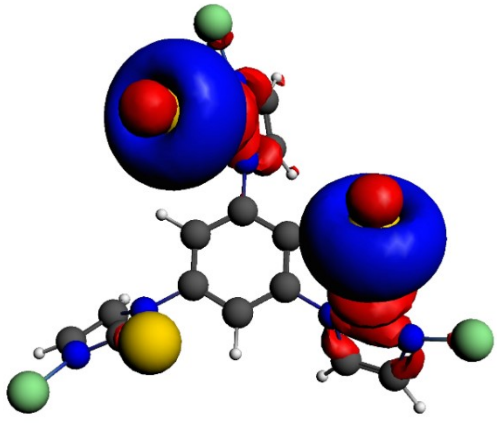

ΔE =-45.39 Kcal/mol
v:0.57219

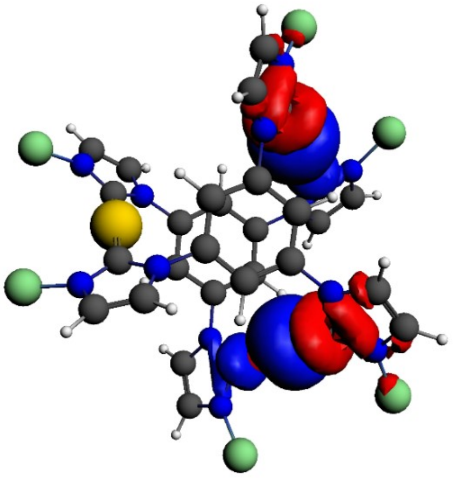

Component A-B of $[L_2(Br)_6 \rightarrow Au_3]^{3+}$

Component A-BA′ of $[L_2(Br)_6 \rightarrow Au_3]^3$

| | | |
|---|---|---|
| Δρ1 | ΔE =-66.53 Kcal/mol<br>v: 0.95963<br>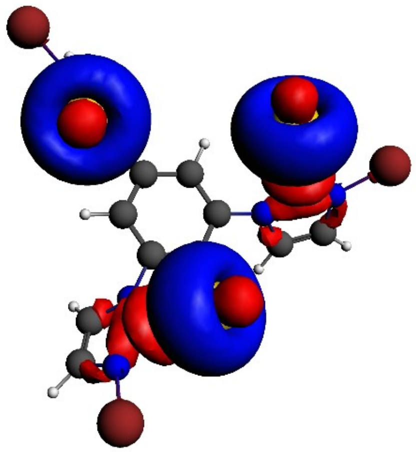 | ΔE =-45.79 Kcal/mol<br>v: 0.58982<br>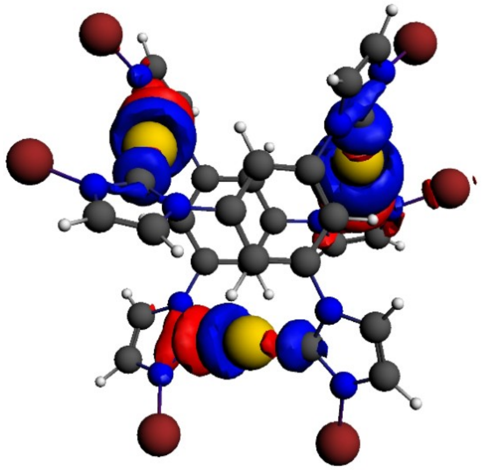 |
| Δρ2 | ΔE =-65.98 Kcal/mol<br>v: 0.95116<br>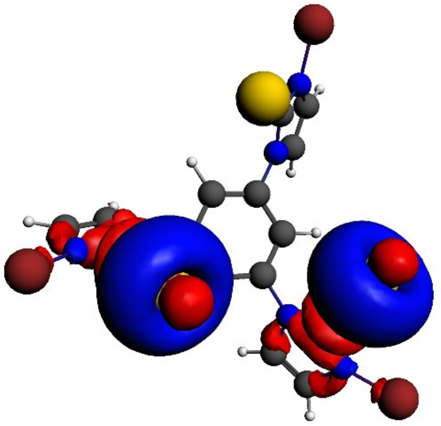 | ΔE =-45.54 Kcal/mol<br>v: 0.58754<br>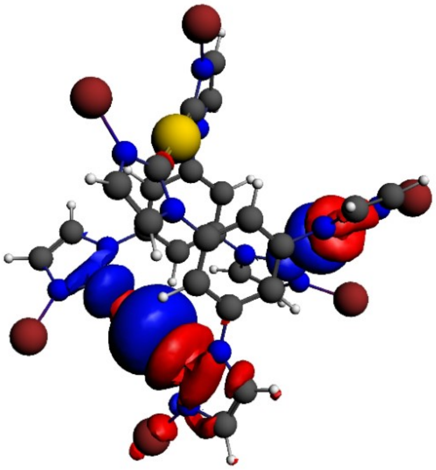 |
| Δρ3 | ΔE =-65.97 Kcal/mol<br>v: 0.95101<br>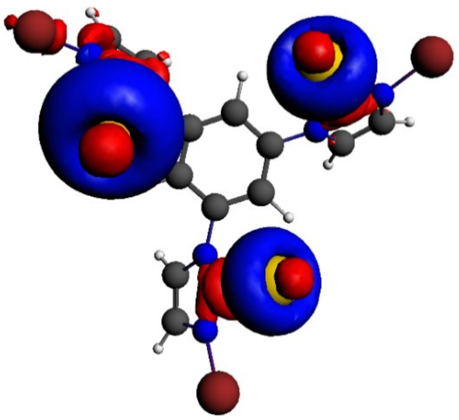 | ΔE =-45.50 Kcal/mol<br>v: 0.58706<br>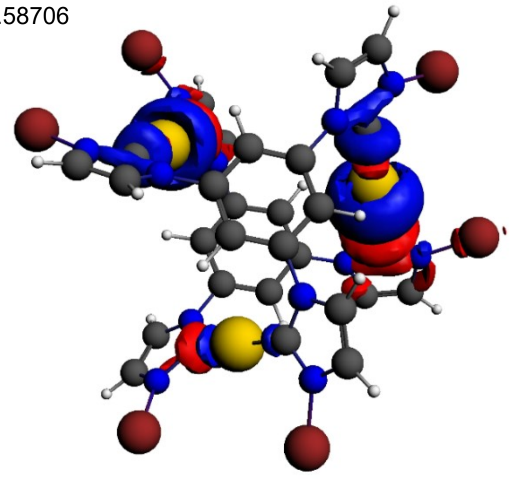 |

Component A-B of $[L_2(SiH_3)_6 \rightarrow Au_3]^{3+}$

Component A-BA′ of $[L_2(SiH_3)_6 \rightarrow Au_3]^{3}$

| | | |
|---|---|---|
| Δρ1 | ΔE =-67.38 Kcal/mol<br>v:0.94812<br>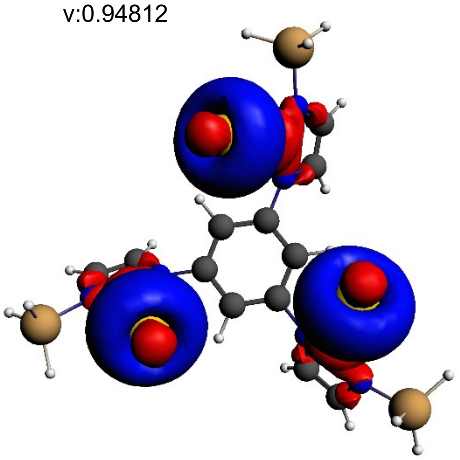 | ΔE =-45.43 Kcal/mol<br>v: 0.56858<br>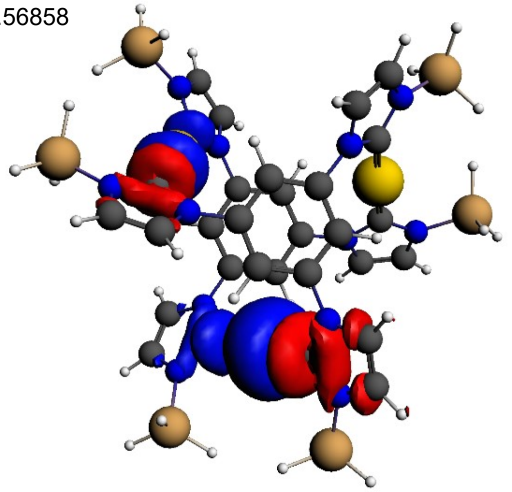 |
| Δρ2 | ΔE =-67.04 Kcal/mol<br>v:0.94229<br>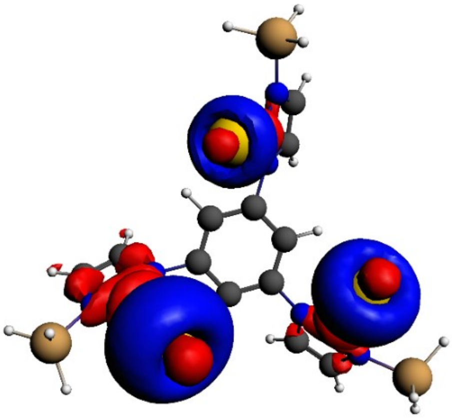 | ΔE =-45.46 Kcal/mol<br>v: 0.56856<br>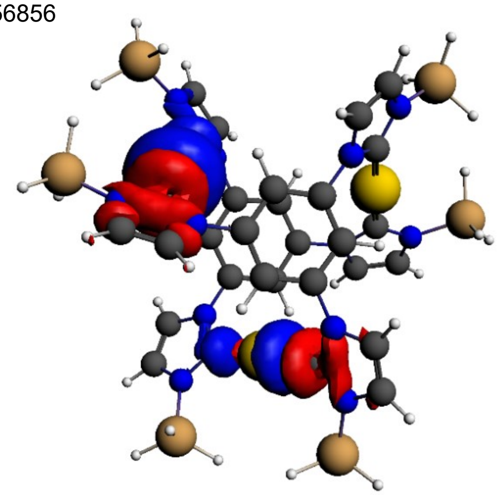 |
| Δρ3 | ΔE =-67.03 Kcal/mol<br>v:0.94219<br>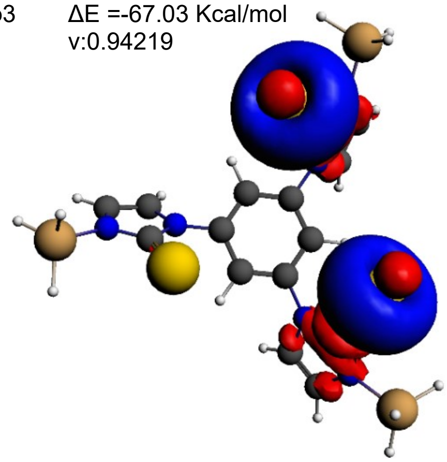 | ΔE =-45.45 Kcal/mol<br>v: 0.56850<br>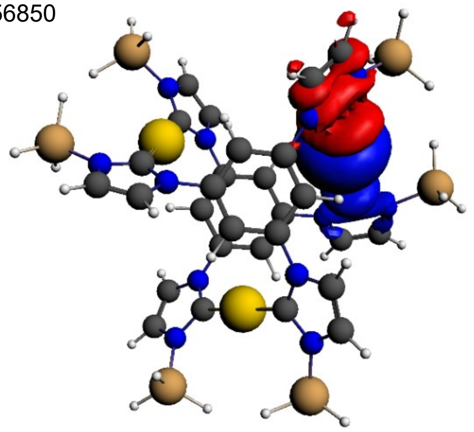 |

Component A-B of $[L_2(Ph)_6\rightarrow Au_3]^{3+}$

Component A-BA′ of $[L_2(Ph)_6\rightarrow Au_3]^{3}$

Δρ1

ΔE =-66.10 Kcal/mol
v:0.94727

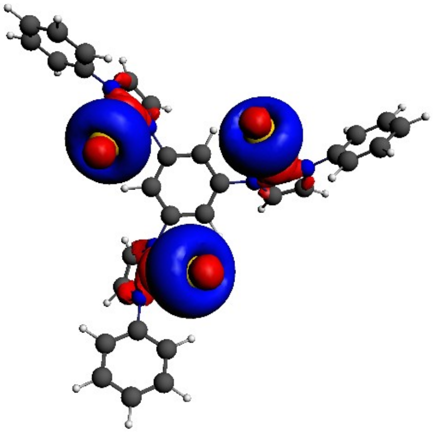

ΔE =-43.92 Kcal/mol
v:0.56808

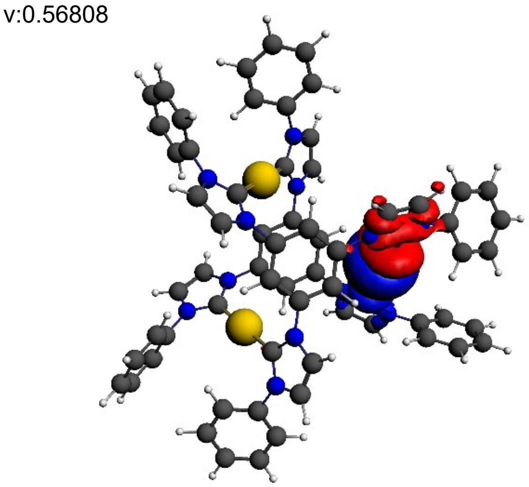

Δρ2

ΔE =-65.72 Kcal/mol
v:0.94031

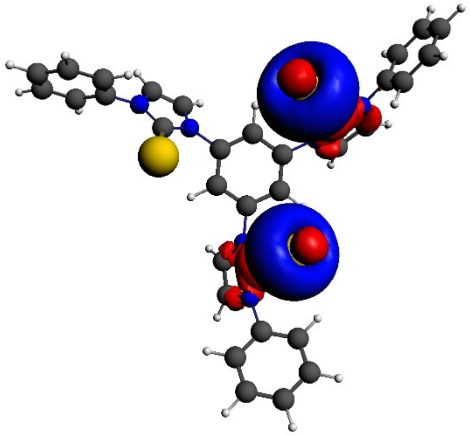

ΔE =-43.85 Kcal/mol
v: 0.56727

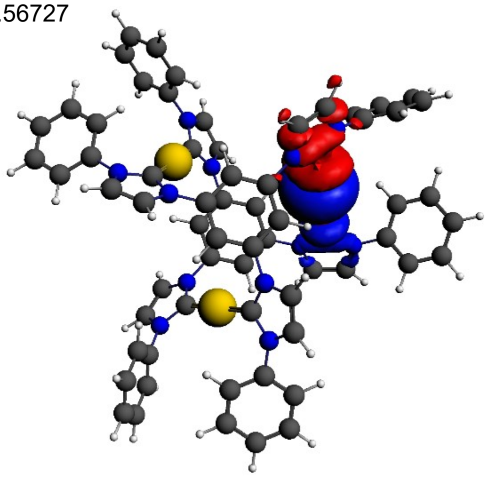

Δρ3

ΔE =-65.71 Kcal/mol
v:0.93876

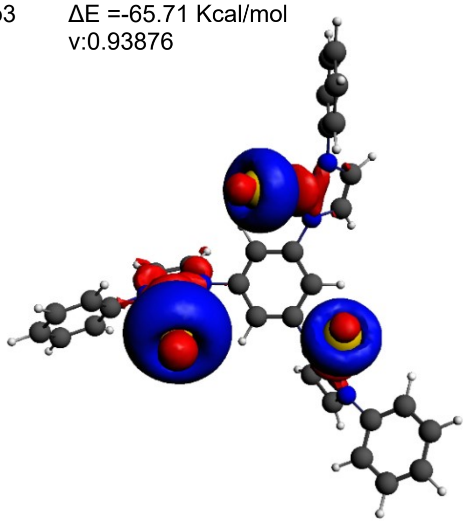

ΔE =-43.59863kcal/mol
v:0.56201

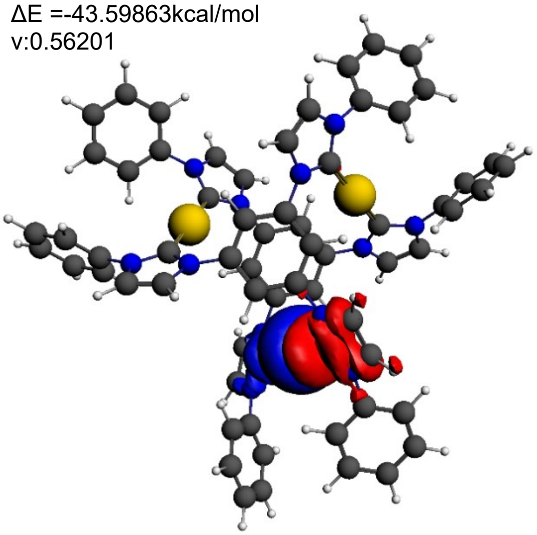

Figure S5: The density of shape change between the Fragments $M^{3+}$ and $L_2(R)_6$, along with the significant energy results of $[L_2(R)_6 \rightarrow Au_3]^{3+}$; R=$C_2H_5$, $CH_3$, H, F, Cl, Br, $SiH_3$ and Ph complexes for A-B and A-BA′ components.
